# Bio‐based and Degradable Block Polyester Pressure‐Sensitive Adhesives

**DOI:** 10.1002/anie.202006807

**Published:** 2020-10-16

**Authors:** Thomas T. D. Chen, Leticia Peña Carrodeguas, Gregory S. Sulley, Georgina L. Gregory, Charlotte K. Williams

**Affiliations:** ^1^ Department of Chemistry University of Oxford Chemistry Research Laboratory 12 Mansfield Rd Oxford OX1 3TA UK

**Keywords:** adhesives, block polyesters, degradable polymers, polymers, ring-opening polymerization

## Abstract

A new class of bio‐based fully degradable block polyesters are pressure‐sensitive adhesives. Bio‐derived monomers are efficiently polymerized to make block polyesters with controlled compositions. They show moderate to high peel adhesions (4–13 N cm^−1^) and controllable storage and loss moduli, and they are removed by adhesive failure. Their properties compare favorably with commercial adhesives or bio‐based polyester formulations but without the need for tackifier or additives.

## Graphical abstract

Well‐defined bio‐based triblock polyesters were prepared using a mixture of renewable monomers and a one‐pot synthetic method with a single catalyst. These materials are pressure‐sensitive adhesives and are easily degraded under acidic conditions.

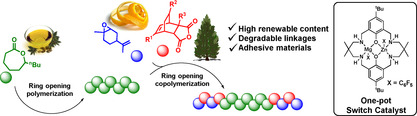

Pressure‐sensitive adhesives (PSA) are self‐adhesive materials that form strong but impermanent bonds with substrates; they are safe to use, easy to handle, removable and show increasing potential to replace conventional adhesives.^[1]^ They are used as laminates, glues, tapes and labels in packaging, automotive components, medical devices, and electronics.^[2]^ PSA undergo instantaneous surface binding with only light pressure without any chemical reaction and are released with little force. They typically comprise a viscoelastic formulation of a copolymer, for example, polyacrylate, natural rubbber or styrenic block polymer, with a tackifier and various additives.[^1^, ^2b^] Except for formulations using natural rubber, most PSA are petrochemicals and nearly all are expected to pervade long‐after use. In a circular materials economy products are re‐used and recycled; delivering this economy requires more sustainable and higher performance PSA, for example, as removable packaging labels and laminates.^[3]^ Various bio‐derived PSA are known, as are adhesives with triggered disassembly,^[4]^ but very few materials combine both approaches. Aliphatic polyesters could fulfill both functions, especially using only bio‐derived monomers and designing the PSA for stability in processing and use, but exploiting variable rates of ester hydrolysis to balance stability in use with potential for complete degradation after use.[^4c^, ^4f^, ^4h^, ^4k^, ^4l^] This communication highlights new single component PSA which are ABA‐block polyesters, where A is a rigid (high glass transition temperature, *T*_g_) block flanking a viscoelastic (low *T*_g_) block B.

The block polyester syntheses should be efficient, controllable and generalizable.^[4f]^ Previously, controlled lactone ring‐opening polymerization (ROP) was used to make polyester PSA.[^4c^, ^4f^, ^4h^, ^4i^, ^4k^, ^4l^] Hillmyer and Tolman reported ABA‐polyesters, made by sequential (−)menthide (terpene) and lactide (carbohydrate) ROP and when mixed with a tackifier these PSA delivered moderate peel forces (3.2 N cm^−1^).^[4k]^ Other copolymers were made by (−)menthide ROP and alkene controlled radical polymerization (carbohydrate), by combining them with tackifier, delivered higher peel forces (9.9 N cm^−1^).^[4c]^ Shin and team prepared block polyester PSA, by ϵ‐decalactone (DL, castor oil) and lactide ROP, which showed moderate peel force (2.6 N cm^−1^).^[4i]^ However, polylactide is a sub‐optimal hard‐block as it fails to confer sufficient tack and its low *T*_g_ (50–60 °C) limits the upper use temperature; alternative bio‐derived hard‐blocks are needed but hard to make by cyclic ester ROP. Epoxide/anhydride ring opening copolymerizations (ROCOP) are highly controlled, easily generalized and produce rigid polyesters in high yields;^[5]^ the resulting alternating polyesters have not yet been investigated as PSA. For example, cyclohexene oxide (CHO)/phthalic anhydride (PA) ROCOP gives a polyester showing 90 °C<*T*_g_<140 °C, but uses petrochemicals.^[6]^ Coates and co‐workers reported high *T*_g_ polyesters, from bio‐derived tricyclic anhydride (TCA, terpene)/propylene oxide (PO, petrochemical) ROCOP (Figure 1),^[7]^ and related high *T*_g_ polyesters were also prepared from bio‐based limonene oxide (LO)/PA ROCOP.^[8]^ Limonene oxide, LO, is an important bio‐derived monomer since it is commercially available, sourced from waste citrus peel, confers naturally high rigidity, delivers an alkene useful for post‐polymerization functionalization and its LCA shows potential reductions to GHG emissions.[^3a^, ^9^] So far, there are no reports of fully bio‐based ROCOP polyesters (PE) although LO/terpene‐derived anhydride ROCOP could afford such materials (Figure 1).

**Figure 1:**
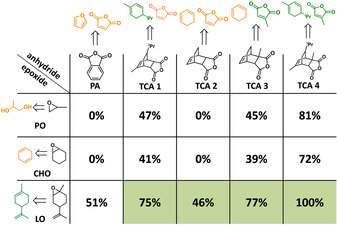
Theoretical renewable content of ROCOP PE from different epoxides/anhydrides (percentage values based on mass content of monomers). Green shading represents structures described in this work, unshaded areas indicate prior work. Orange (potentially renewable) and green (fully renewable) are used to discriminate monomer sources (Schemes S1–S8 outline the monomer syntheses and sources).

Here, new bioderived polyesters (PE) are prepared by LO/tricyclic anhydride (TCA 1–4) ROCOP. Polymerizations applied complex **1**, [LMgZn(C_6_F_5_)_2_], and 1,4‐benzene dimethanol (BDM) as the initiating system and were conducted at 140 °C (Figure 1, Table S1, Figure S1, S2). The catalyst is highly active and selective for mechanistically related epoxide/CO_2_(or anhydride) ROCOP and features non‐initiating co‐ligands which allow for control over initiation and the chain end‐groups to selectively deliver ABA‐block structures.^[10]^ First, the catalyst was tested in LO/TCA(1–4) ROCOP, in all cases reaching quantitative anhydride conversion over a few days (>99 %) (Table S2). The reactions are slower than CHO/CO_2_ ROCOP because of the steric hindrance of both LO and TCA, but the catalyst maintains constant activity.^[10a]^ Monomer selectivity is high, producing perfectly alternating PE (Figure S3). In terms of the LO stereochemistry, complete conversion was achieved using *trans*‐LO, but no conversion resulted from *cis*‐LO (Table S2). Commercial LO (*cis*/*trans*‐LO mixture) was applied in all reactions and the catalyst reacts selectively with *trans*‐LO, with *cis*‐LO serving as the solvent. Polymerizations show linear fits to molar mass (*M*_n_) vs. conversion data, indicative of good control (Figure S4). SEC analyses show monomodal distributions throughout polymerizations (*Đ*≤1.19) (Figure S5). Conversion vs. time data are linear and consistent with zeroth order anhydride kinetics (Figure S6).[^5^, ^11^]

In terms of the polymer chain end‐groups, MALDI‐ToF analysis reveals two populations, each separated by the expected repeat unit (386 *m*/*z*) (Figure S8). The major population is the desired α,ω‐hydroxyl telechelic PE and the other is cyclic polyester. The presence of cyclic species is without precedent using **1** (or related catalysts),[^6b^, ^10b^, ^12^] and surprising as these sterically hindered monomers should undergo only limited transesterification. To investigate, LO/TCA1 ROCOP was maintained at 140 °C for 7 days beyond full conversion and the PE maintains the same molar mass and monomodal distribution (*Đ*≤1.19), that is, the absolute rate of transesterification appears insignificant (Figure S9). All PE show the desired high *T*_g_ (82–102 °C) and, as expected, the least hindered anhydride (TCA2) shows the highest value (Table S2).^[13]^

With an effective route to bio‐based “hard” PE established, the “soft” block synthesis was investigated. Poly(*ϵ*‐decalactone) (PDL) is selected due to its low *T*_g_ and track record for phase‐separation in other block polyesters.[^10b^, ^14^] The catalyst **1**/BDM is highly active for DL ROP, with TOF >6800 h^−1^ (80 °C) (Table S3). The reaction yields high molar mass PDL (*M*_n_>30 kg mol^−1^) with good control (*Đ*≤1.11). As ROP kinetics are first order in DL, at higher conversions the rate decreases and transesterification occurs (Figure S10). Thus, the best conditions to make ABA‐polyesters are at 50–60 % DL conversion.

Previously switchable polymerizations were discovered whereby monomer mixtures are selectively converted into block polymers, using a single catalyst that accesses different catalytic cycles.[^9i^, ^10b^, ^10c^, ^12a^, ^15^] In these reactions epoxide/anhydride ROCOP generally occurs before lactone ROP, thus, the addition/removal of anhydride allows switching of polymerization mechanism.[^9i^, ^10b^, ^12^, ^15a^, ^15b^, ^15d^] To establish its potential with sterically hindered monomers, the catalyst was reacted with DL, using LO as solvent to selectively form PDL. At the required PDL conversion, TCA1 was added (and temperature increased to 140 °C) which “switched” the polymerization to LO/TCA ROCOP (Figure S11). This observation is rationalized by both faster TCA (vs. DL) insertion into the alkoxide intermediate and formation of a more stable carboxylate linkage (vs. alkoxide) (Figure S12).^[12b]^ The switchable polymerization efficiently yields the desired ABA‐polyesters, in one‐pot with one catalyst, and allows for control of both polyester composition and molar mass.

Switchable polymerizations were used to make seven new polyesters: one series comprising PE from LO/TCA1 with variable hard block compositions (wt %_PE_=20–50 %) and another with fixed PE composition (wt %_PE_≈40 %) but different TCAs (Table 1, Figures S13–S16). All block polyester have high renewable contents and show high molar masses (*M_n_*≈40 kg mol^−1^) in close agreement between experimental and theoretical (targeted) molar masses (by NMR and SEC). The polymerizations are well controlled, as indicated by a continual evolution in molar mass values while retaining monomodal, narrow dispersity distributions (*Đ*≤1.10). The reactions are highly selective for ABA‐polyesters, as characterized by a range of techniques. For example, P3 synthesis involves DL ROP (*M*_n_=29 kg mol^−1^) and ^31^P{^1^H} NMR end group analysis shows a PDL singlet (Figure 2 A,B). After PE formation, the block polyester *M*_n_ increases to 41 kg mol^−1^ and end‐group analysis shows complete loss of the PDL signal and two new sets of peaks identical to those of pure PE (Figures 2 A,B, S17). DOSY NMR shows a single diffusion coefficient for all resonances, whereas the corresponding polymer blend shows two coefficients (Figure 2 C, S18). COSY NMR identifies the block junction resonances (Figure 2 D, S19). Switchable polymerization was successful at different DL/LO/TCA1 loadings and using different anhydrides (TCA2‐4). For every new polymer the complete range of characterization techniques were applied (Table 1, Figures S20–S35). All polymers show *T*_*d,5 %*_ from 260–270 °C which is within the range for PSA processing and use (Figures S26–S29).

**Figure 2:**
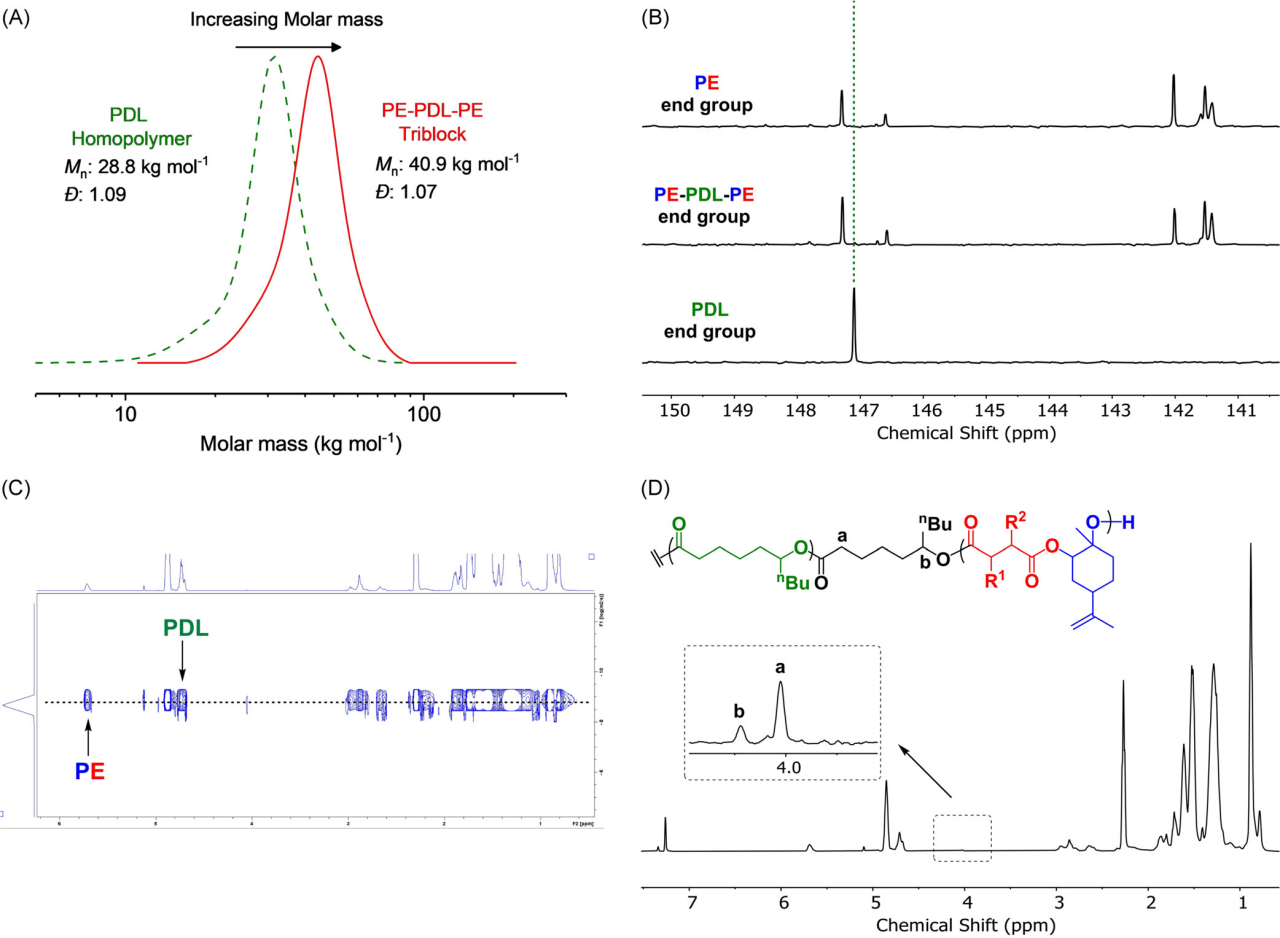
Characterization data for P3 (Table 1): A) SEC traces showing increasing molar mass but retained narrow *Đ* (in THF at 30 °C, calibrated using polystyrene standards). B) Selected region of ^31^P{^1^H} NMR spectra of polymers, showing the evolving end group resonances as polymerizations progress. C) ^1^H DOSY NMR spectrum showing a single diffusion coefficient. D) ^1^H NMR spectrum illustrating polymer chain junction units (in CDCl_3_).

**Table 1:** ABA‐Polyester Synthesis Using Switchable Polymerization Catalysis 
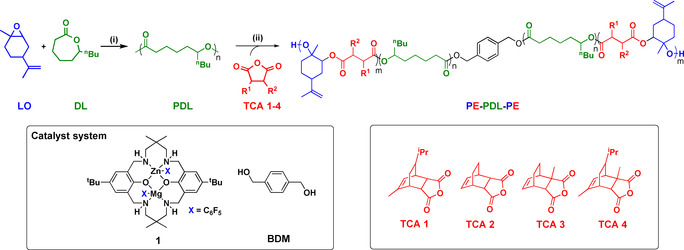

Sample	TCA used	*M*_n, NMR_ [kg mol^−1^]^[a]^			Wt%_Hard_^[b]^	*M*_n_, _SEC_ [kg mol^−1^][*Đ*]^[c]^		*T*_g_ [°C]^[d]^	Renewable content [%]^[e]^
		PDL	PE	Triblock		PDL	Triblock		
P1	TCA 1	29.3	7.7	37.0	21	34.3 [1.09]	39.1 [1.08]	−44, 72	95
P2	TCA 1	25.2	10.8	36.0	30	31.8 [1.09]	40.2 [1.07]	−40, 70	93
P3	TCA 1	22.5	15.5	37.9	41	28.8 [1.09]	40.9 [1.07]	−31, 79	90
P4	TCA 1	17.7	17.0	34.7	49	26.2 [1.08]	34.7 [1.06]	−21, 103	88
P5	TCA 2	23.8	16.5	40.4	41	34.3 [1.08]	45.3 [1.05]	−34, 79	78
P6	TCA 3	21.8	15.2	36.9	41	28.0 [1.07]	38.2 [1.05]	−31, 81	91
P7	TCA 4	21.8	14.4	36.2	40	27.7 [1.08]	38.1 [1.10]	−30, 70	100

(i): [**1**]/[BDM]/[DL]/[LO]=1/4/800–1200/2000, 60 °C, 4–7 min. (ii): [**1**]/[TCA]=1/75–225, 140 °C, 32–138 h (Table S1). [a] Obtained from ^1^H NMR spectra (Figure S13–S16). [b] PE weight %, from ^1^H NMR spectra. [c] *M*_n_ and *Đ* measured by SEC, calibrated using polystyrene standards (Figures S20–S25). [d] Glass transition temperatures by DSC (third heating cycles, lower *T*_g_) (Figures S36–S42) and maxima in tan(δ) from DMA (1 Hz, 2 °C min^−1^, upper *T*_g_) (Figure S43–S49). [e] Theoretical renewable content (Schemes S1–S8).

Using block polymers as PSA requires a careful balancing of properties. Block phase separation is desirable, with the soft phase (e.g. PDL) minimizing the adhesive surface energy and improving tack, whilst the physically networked hard phase (e.g. PE) confers mechanical strength and resists shear forces.[^4i^, ^4k^, ^16^] These polyesters are all amorphous and have phase separated microstructures, as indicated by DSC and dynamic mechanical thermal analysis (DMTA). DSC reveals lower *T*_g_ values similar to pure PDL (−50 °C) (Figures S36–S42) but the upper *T*_g_ is hard to observe or very low intensity.[^10b^, ^12a^] The upper *T*_g_ is clearly apparent as a maximum tan(δ) using DMTA and occurs from 70 to 103 °C, consistent with values for pure PE (Figures S43–S49). It is worth noting that the blocks are not miscible as the *T*_g_ does not correspond to those calculated using the Fox equation for miscible polymers (Table S4).

The adhesive properties were investigated by 180° peel adhesion tests using stainless steel plates at room temperature (ISO 29 862:2018). For P1–P4, as PE content increases (21–41 wt %) so the peel adhesion increases significantly (0.1–10.8 N cm^−1^). This is attributed to the increased physical crosslink density increasing the mechanical strength. P5, with 49 wt % PE content, shows lower peel adhesion (4.0 N cm^−1^), possibly due to reduced substrate binding. The performances of P1, 2 and 4 align with peel adhesions of “Post‐it” notes, Scotch tape and Duct tape, respectively and P3 is more than twice as strong as Duct tape (Figure 3 A). Desirable adhesive failure (i.e. without residue) is shown by samples with 40 wt %+ PE, consistent with their higher strengths (Figure S50A). P3, 5–8, prepared from different TCAs, all show high peel adhesions (8.1–13.1 N cm^−1^), undergo adhesive failure and compare favorably against literature bio‐derived polymer PSA‐tackifier blends (Figures 3 B, S51).[^4e^, ^4i^] Note that all these literature PSA are bio‐derived but not all are degradable, for example, the acrylic/ester PSA which shows the best peel adhesion values would not be fully degraded.^[4c]^ Additional comparisons with other bio‐based examples in the literature, including polyesters,^[17]^ polyhydroxyalkanoates (PHA),^[18]^ plant oil derived polymers^[19]^ and polycarbonates,^[4a]^ are included in Table S5.

**Figure 3:**
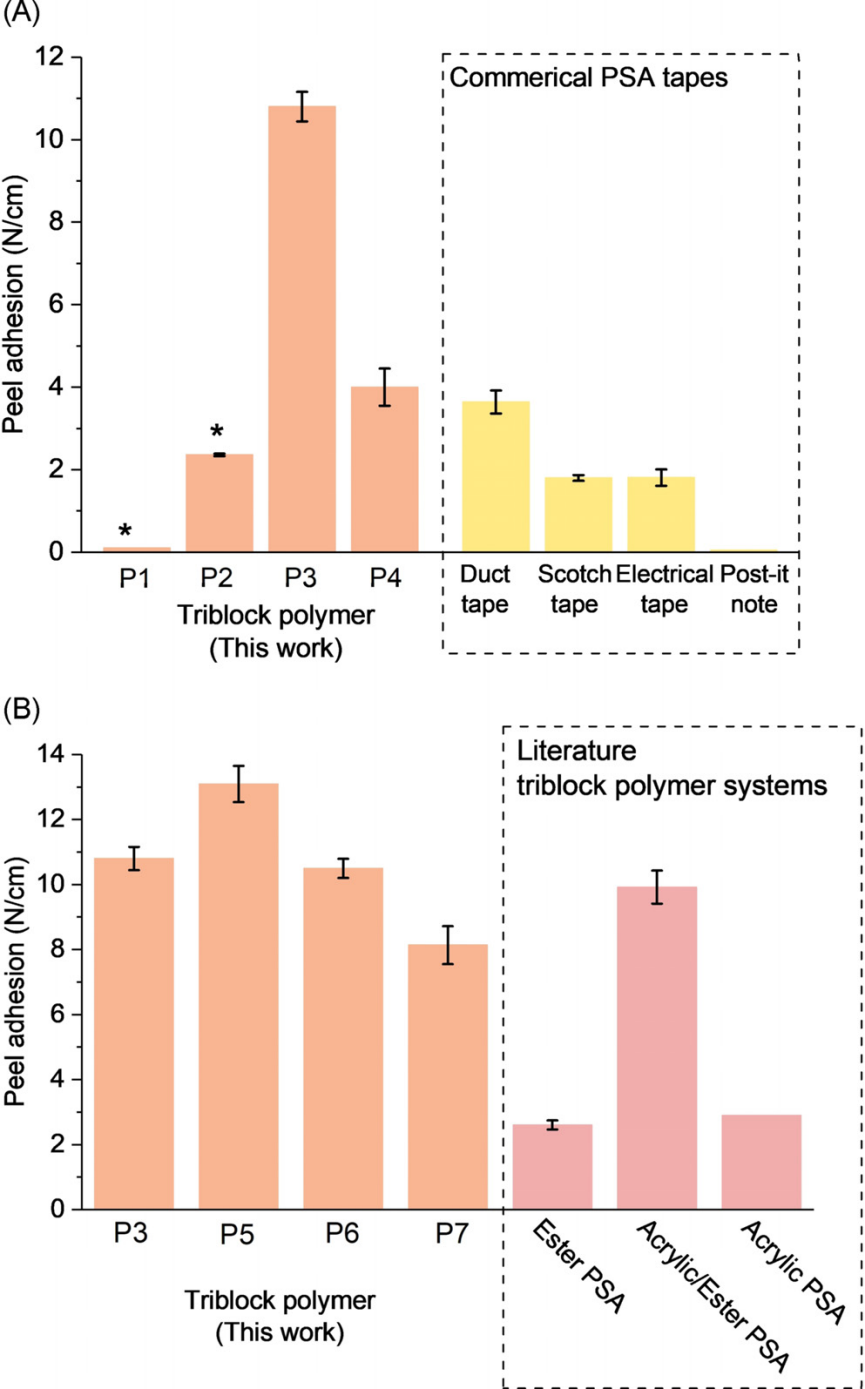
Peel adhesion results. A) Compares P1–P4 with commerical PSAs. B) Compares P3, P5–P7 with literature bioderived polyester PSAs (Figure S51 for polymer structures).[^4c^, ^4e^, ^4i^]

For adhesives the bonding and debonding frequencies are an important measure of materials response to shear forces both during application and removal.^[2b]^ The adhesion and peel behaviors correlate with the bonding frequency (at ≈0.1 rad s^−1^) and debonding frequency (at ≈100 rad s^−1^), respectively. Frequency sweep rheology outputs, from 0.01 (0.0628 rad s^−1^) to 30 Hz (188 rad s^−1^) at room temperature, were plotted against the shear modulus, *G* (Figures 4 A, S52–S57).[^16^, ^20^] At the bonding frequency, *G*′ needs to fall below the Dahlquist criterion (*G*′≤3×10^5^ Pa) so as to promote substrate wetting.[^4i^, ^4k^, ^20^] PDL alone shows a sufficiently low *G*′ to fulfill this criterion but it is not a PSA as it lacks resistance to shear, which can be delivered in block polymers by the hard block. However, in many block polymer PSA, the hard block increases the storage modulus too much and tackifers are needed to reduce *G*′.[^2b^, ^4e^] In this work, all the block polyesters show shear storage moduli below the Dahlquist criterion at the bonding frequency, that is, they should all be effective PSA without needing additives. PSA application potentials were analyzed according to the quadrant method (Figure S58).^[20]^ For each sample, a viscoelastic window is superimposed onto the *G*′ vs. *G*′′ plot and the quadrant provides a qualitative assessment of application.^[21]^ For instance, P2‐3 are general purpose PSA, and P3 shows high shear, whilst P2 should be an easily removable PSA (Figure 4 B). P2 underwent cohesive failure which would be a disadvantage for a removable application, but the failure mechanism could be addressed by increasing *M*_n_ or by modifying the soft block.

**Figure 4:**
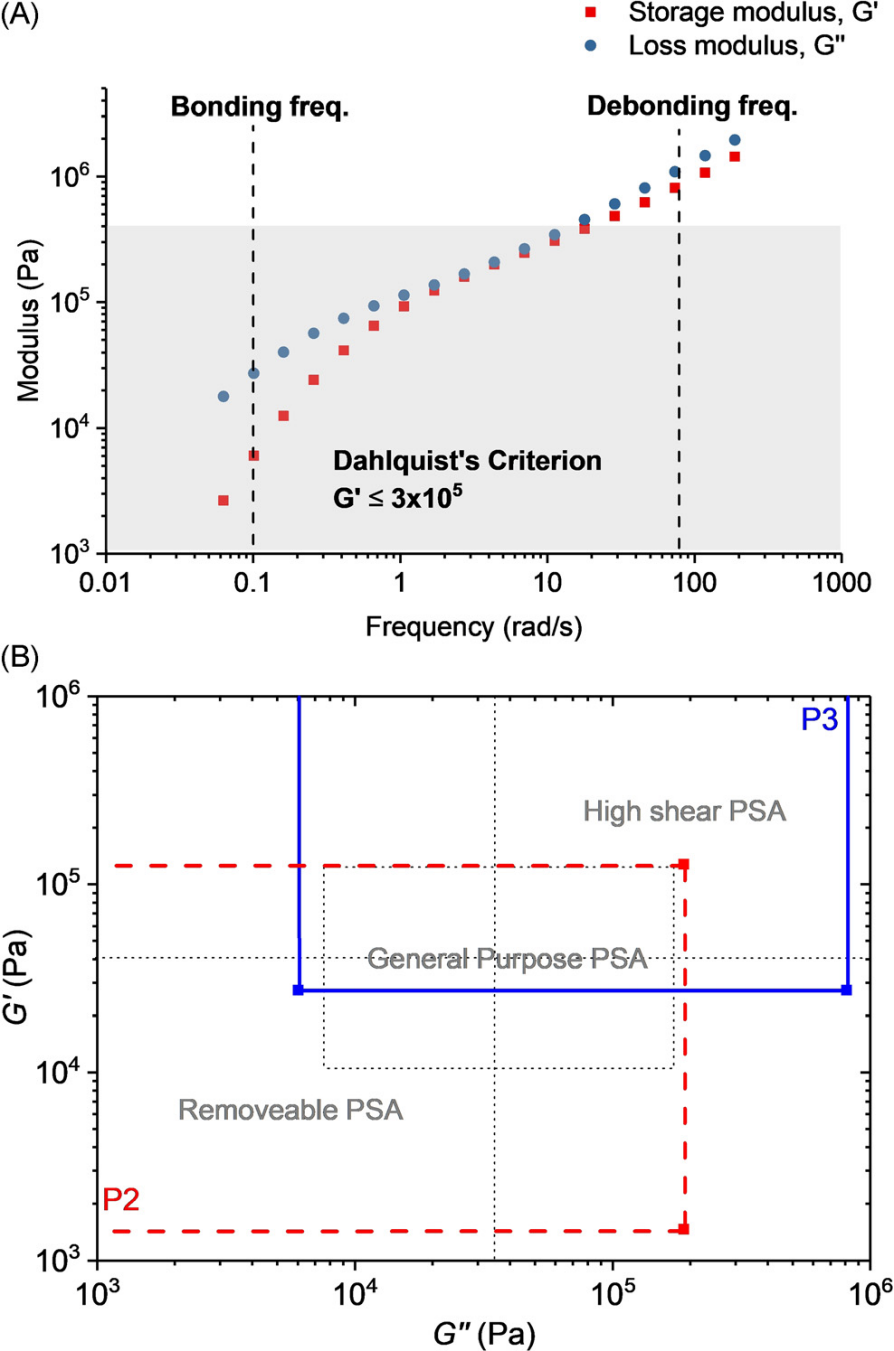
A) Storage and loss moduli vs. frequency plots for P3. B) PSA performance windows for P2 and P3.

One disadvantage of most commercial or literature block polymer PSA is that their structures pervade long after use. Such stability is, of course, advantageous in application but excessive longevity is undesirable and PSA that completely degrade are important targets. Accordingly solutions of these polyesters undergo rapid degradation when reacted with an organic acid (*p*‐toluenesulfonic acid, *p*‐TSA, 1 M) at 60 °C. Under these conditions, polymer molar mass rapidly decreases (>98 % mass loss) within 5 h (Figures 5, S59, S60). Because these block polyesters can be applied without additives, they are also expected to be amenable to re‐processing recycling options, and future investigations into closed loop recycling are warranted. Additionally, further enhancements to the adhesive performances may be achieved by exploiting side‐chain functionalization to furnish the polymer with chemical groups that introduce non‐covalent interactions (e.g. hydrogen bonding) as these have previously shown great promise in other polymeric systems.^[21]^

**Figure 5:**
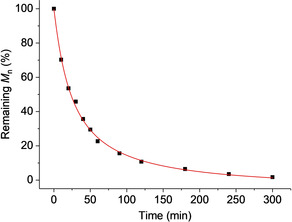
Complete degradation of P3. Reaction conducted in THF and with *p*‐toluene sulfonic acid (1 M) at 60 °C.

A new class of bioderived and fully degradable block polyester pressure sensitive adhesives were easily prepared. Seven ABA‐polyesters were synthesized by the selective polymerization of bioderived limonene oxide, tricyclic anhydrides and ϵ‐decalactone, exploiting a one‐pot process with a single catalyst. Polymerizations were highly controlled and selective, enabling tuning of the polyesters’ molar masses and compositions. The polyesters were effective single component PSA obviating tackifier resin and additive usage. Their mechanical and rheological properties align with existing commercial materials but with the benefit of being easily triggered to completely degrade. These findings should stimulate performance and application development studies focused on sectors where closed loop recycling and PSA disassembly is required. The straightforward synthetic methods should be extended to other commercial and bio‐based monomers and by varying composition these polyesters may address other application sectors, for example as degradable elastomers, ductile plastics or medical materials.

Please note: Minor changes have been made to this manuscript (structure of LO corrected in Figure 1) after its appearance in Angewandte Chemie Early View. The Editor.

## Conflict of interest

The authors declare no conflict of interest.

## Supporting information

As a service to our authors and readers, this journal provides supporting information supplied by the authors. Such materials are peer reviewed and may be re‐organized for online delivery, but are not copy‐edited or typeset. Technical support issues arising from supporting information (other than missing files) should be addressed to the authors.

Supplementary
