# Supplementary material for: Bio‐based and Degradable Block Polyester Pressure‐Sensitive Adhesives
Source: Angew Chem Int Ed Engl. 2020 Oct 16;59(52):23450–5. doi: 10.1002/anie.202006807 (PMC7756385; doi:10.1002/anie.202006807)
Supplement: Supplementary file 1 — Supplementary [file ANIE-59-23450-s001.pdf]

Angewandte Chemie
Eine Zeitschrift der Gesellschaft Deutscher Chemiker

Supporting Information

Bio-based and Degradable Block Polyester Pressure-Sensitive Adhesives

*Thomas T. D. Chen, Leticia Peña Carrodeguas, Gregory S. Sulley, Georgina L. Gregory, and Charlotte K. Williams**

anie_202006807_sm_miscellaneous_information.pdf

Contents

1. Experimental 4
   1.1. General Procedure 4
   1.2. Procedures 4
      Synthesis of [LMgZn(C_6F_5)$_2$] (1) 4
      End group analysis by $^{31}P\{^1H\}$ NMR spectroscopy 5
      Ring-opening polymerization of DL 5
      Exemplar ring-opening copolymerization of LO and TCA 5
      Exemplar terpolymerization of DL, LO and TCA 5
      Degradation experiments 6
   1.3. Supporting schemes, figure and tables 7
      Scheme S1. Industrial and potential renewable routes to phthalic anhydride 7
      Scheme S2. Industrial and potential renewable routes to maleic anhydride 7
      Scheme S3. Industrial and potential renewable routes to 1,3-cyclohexadiene 8
      Scheme S4. Renewable routes to α-phellandrene 8
      Scheme S5. Renewable routes to citraconic anhydride 9
      Scheme S6. Industrial and potential renewable routes to propylene oxide 9
      Scheme S7. Industrial and potential renewable routes to cyclohexene oxide 10
      Scheme S8. Renewable routes to limonene oxide 10
      Scheme S9. In situ activation of the catalyst from reaction between 1 and 1,4-Benzenedimethanol (BDM) 11
      Figure S1. ^1H NMR spectrum of [LMgZn(C_6F_5)$_2$], 1, in $CDCl_3$ at 298 K. 11
      Figure S2. $^{13}C\{^1H\}$ NMR spectrum of [LMgZn(C_6F_5)$_2$], 1, in $CDCl_3$ at 298 K. 12
      Table S2. ROCOP of LO and 4 different TCA 13
      Figure S3. ^1H NMR spectrum of the polyester from the ROCOP of LO/TCA 1 in $CDCl_3$. 14
      Figure S4. M_n and $Đ$ vs. conversion for the ROCOP of LO/TCA 1 14
      Figure S5. SEC traces of aliquots obtained in thee ROCOP of LO/TCA 1 15
      Figure S6. Conversion vs. time for the ROCOP of LO/TCA 1 15
      Figure S7. HSQC NMR spectrum of the polyester from the ROCOP of LO/TCA 1 in $CDCl_3$. 16
      Figure S8. Representative MALDI-ToF of the polyester from the ROCOP of LO/TCA1. 17
      Figure S9. SEC trace for the ROCOP of LO/TCA 1 after deliberate heating at 140 °C for 7 days (after full conversion). 17
      Table S3. ROP of DL 18
      Figure S10 Illustration of the DL ROP and transesterification mechanisms. 18

Figure S11. Conversion vs. time plot for the selectivity study between ROP of DL and ROCOP of LO/TCA 1. 19

Figure S12. Proposed ‘switching’ mechanism for ROCOP of LO/TCA and ROP of DL using a single catalyst 19

Figure S13. ^{1}H NMR spectrum of P3 (in $CDCl_3$). 20

Figure S14. ^{1}H NMR spectrum of P5 (in $CDCl_3$). 20

Figure S15. ^{1}H NMR spectrum of P6 (in $CDCl_3$). 21

Figure S16. ^{1}H NMR spectrum of P7 in ($CDCl_3$). 21

Figure S18. Homopolymer blend with similar molar masses to the respective blocks in P3 showing two different coefficients in $CDCl_3$. 23

Figure S19. COSY NMR spectrum of P3 in $CDCl_3$. 24

Figure S21 Stacked SEC traces of P2 illustrating an increase molar mass with retention of very narrow *Đ* (in THF at 30 °C, calibrated using polystyrene standards). 25

Figure S23. Stacked SEC traces of P5 illustrating an increase molar mass with retention of very narrow *Đ* (in THF at 30 °C, calibrated using polystyrene standards). 26

Figure S24. Stacked SEC traces of P6 illustrating an increase molar mass with retention of very narrow *Đ* (in THF at 30 °C, calibrated using polystyrene standards). 27

Figure S25. Stacked SEC traces of P7 illustrating an increase molar mass with retention of very narrow *Đ* (in THF at 30 °C, calibrated using polystyrene standards). 27

Figure S30. Selected region of $^{31}P\{^1H\}$ NMR spectra of P5 hydroxyl polymers showing a complete change in end group resonance (in $CDCl_3$). 30

Figure S31. ^{1}H DOSY NMR spectrum showing a single diffusion coefficient for P5 (in $CDCl_3$). 30

Figure S32. Selected region of $^{31}P\{^1H\}$ NMR spectra of P6 hydroxyl polymers showing a complete change in end group resonance (in $CDCl_3$). 31

Figure S33. ^{1}H DOSY NMR spectrum showing a single diffusion coefficient for P6 (in $CDCl_3$). 31

Figure S34. Selected region of $^{31}P\{^1H\}$ NMR spectra of P7 hydroxyl polymers showing a complete change in end group resonance (in $CDCl_3$). 32

Figure S35. ^{1}H DOSY NMR spectrum showing a single diffusion coefficient for P7 (in $CDCl_3$). 32

Figure S36. DSC thermography for P1 33

Figure S37. DSC thermography for P2 33

Figure S38. DSC thermography for P3 34

Figure S39. DSC thermography for P4 34

Figure S40. DSC thermography for P5 35

Figure S41. DSC thermography for P6 35
Figure S42. DSC thermography for P7 36
Figure S43. Storage & loss modulus and tan(δ) vs. temperature plot for P1 36
Figure S44. Storage & loss modulus and tan(δ) vs. temperature plot for P2 37
Figure S45. Storage & loss modulus and tan(δ) vs. temperature plot for P3 37
Figure S46. Storage & loss modulus and tan(δ) vs. temperature plot for P4 38
Figure S47. Storage & loss modulus and tan(δ) vs. temperature plot for P5. 38
Figure S48. Storage & loss modulus and tan(δ) vs. temperature plot for P6 39
Figure S52. Storage & loss modulus vs. frequency plot for P1. 41
Figure S53. Storage & loss modulus vs. frequency plot for P2 41
Figure S54. Storage & loss modulus vs. frequency plot for P4 42
Figure S55. Storage & loss modulus vs. frequency plot for P5 42
Figure S56. Storage & loss modulus vs. frequency plot for P6 43
Figure S57. Storage & loss modulus vs. frequency plot for P7. 43
Figure S59.Plot of Ln(Mn_0/M_n) vs. Time for P3 under acid-catalyzed degradation (THF, *p*TSA, 60 °C) 44
Figure S60. Stacked SEC traces for the acid degradation test on P3 (1M in THF, 60 °C). 45
Figure S61. ^{1}H NMR of the polyester from the ROCOP of LO/TCA 2 in $CDCl_3$ 45
Figure S62. ^{1}H NMR of the polyester from the ROCOP of LO/TCA 3 in $CDCl_3$ 46
Figure S63. ^{1}H NMR of the polyester from the ROCOP of LO/TCA 4 in $CDCl_3$ 46
Table S4. Theoretical T_g for fully miscible blocks 47
Table S5. Adhesive performance comparison against selected examples of bio-based polymers in the literature 48
2. References 49

1. Experimental

1.1. General Procedure

All solvents and reagents were purchased and used as obtained from commercial sources (Sigma Aldrich) unless stated otherwise. The synthesis of [H_2L] macrocyclic ligand, was carried out in air and performed according to literature procedures.[1] The synthesis of the catalyst, [$LMgZn(C_6F_5)_2$], monomer purification and subsequent polymerizations were carried out under inert conditions using standard Schlenk line techniques and a nitrogen-filled glovebox.

Limonene oxide (LO) was purified by first stirring over NaH followed by addition of MeI and fractional distillation at 40 °C (1 mbar). This procedure was repeated twice more (three distillations in total) then kept under a nitrogen atmosphere. ε-decalactone (DL) had been dried over CaH_2, distilled under reduced pressure twice and kept under a nitrogen atmosphere. 1,4-Benzenedimethanol (BDM) had been recrystallised from toluene three times and kept under nitrogen. The tricyclic anhydrides (TCAs) were synthesised and purified based on modified literature procedures.[2] Sublimation of the TCAs (1-3) was performed at least twice and column chromatography of TCA 4 was performed four times in order to enhance the purity of the TCA monomers.

NMR spectra were obtained using a Bruker AV 400 instrument. SEC data was obtained using a Shimadzu LC-20AD instrument with HPLC grade THF as the eluent flowing at 1.0 mL/min at 30 °C and monodisperse polystyrene standard was used for calibration. Elemental analysis was performed by Mr. Stephen Boyer at London Metropolitan University, North Campus, Holland Road, London, N7. The thermal properties were measured using DSC3+ (Mettler Toledo, Ltd). A sealed empty crucible was used as a reference, and the DSC was calibrated using indium. Samples were heated from 25 °C to 150 °C, at a rate of 10 °C/min, under N_2 flow (80 ml/min), and were kept at 150 °C for 2 min to erase the thermal history. Subsequently, the samples were cooled to -80 °C, at a rate of 10 ^{o}C/min, and kept at -80 °C·min^{-1} for a further 2 mins, followed by a heating procedure from -80 °C to 150 °C, at a rate of 10 °C/min. Each sample was run for two heating−cooling cycles. The glass transition temperatures (T_g) reported are taken from the third heating cycle. TGA was measured using a TGA/DSC 1 system (Mettler-Toledo Ltd). Samples were heated from 25 °C to 500 °C, at a rate of 5 °C/min, under N_2 flow (100 cm^3/min). Viscoelastic properties were measured using an Anton Paar Physica MCR 301 rheometer. Temperature sweep tests were performed at a constant shear strain of 0.5% and a frequency of 1 Hz from 20 °C to 150 °C at 2 °C/min. Frequency sweep tests were performed at a constant shear strain of 0.5% from 0.01 Hz to 30 Hz at 25 °C. The 180 ° Peel test was performed according to ASTM D3000 standard testing methods using a Shimadzu EZ-LX Universal Testing Instrument at a peeling rate of 305 mm/min (ISO 29862:2018). The adhesive material was placed on a PET sheet (0.5 inch width) and adhered to a polished stainless steel test panel with a constant pressure provided by a 4.5 lb weight. The average peel force was collected and reported from three samples.

1.2. Procedures

Synthesis of [$LMgZn(C_6F_5)_2$] (1)

Under inert conditions, the [H_2L] macrocyclic ligand (500 mg, 0.90 mmol) and magnesium bis(hexamethyldisilazide) ($Mg(N(SiMe_3)_2)_2$)(312 mg, 0.90 mmol) were stirred in anhydrous THF (10 mL) at 25 °C for 1 h. A solution of bis(pentafluorophenyl) zinc (361 mg, 0.90mmol),

in anhydrous THF (5 mL), was then added dropwise to the ligand/$MgHMDS_2$ mixture which was stirred overnight at 25 °C to afford a cloudy orange solution. The solvent was filtered and washed with pentane (2 x 20 mL) to yield a white solid (0.72 mg, 81%).

^{1}H NMR (400 MHz, $CDCl_3$, 298 K) δ(ppm): 6.81 (d, 2H, J=3 Hz, b'), 6.75 (d, 2H, J=3 Hz, b), 4.36 (t, 2H, J=13 Hz, c'), 4.30 (t, 2H, J=13 Hz, c), 3.38 (d, 2H, J=14 Hz, d), 3.24 (d, 2H, J=13 Hz, d'), 3.08 (t, 2H, J=13 Hz, f'), 3.01 (t, 2H, J=13 Hz, f) 2.77-2.62 (m, 6H, g+g'+e), 2.10-2.02 (m, 2H, e'), 1.26 (s, 3H, h), 1.20 (s, 18H, a), 1.16 (s, 3H, h'), 1.06-1.00 (m, 6H, i+i'). ^{13}C{^{1}H} (125 MHz, $CDCl_3$, 298 K) δ(ppm): 159.4 (f), 137.3 (c), 126.5 (d+d'), 124.7 (e'), 122.2.5 (e), 62.7 (h), 61.9 (h'), 58.0 (g), 56.1 (g'), 34.4 (i + i'), 33.6 (b), 31.7 (a) 28.5 – 20.3 (j+j' and k+k'). Elemental analysis: Calcd for $C_{46}H_{54}F_{10}MgN_4O_2Zn$ (%): C 56.69, H 5.58, N 5.75. Found: C 56.55, H 5.61, N 5.68.

End group analysis by ^{31}P{^{1}H} NMR spectroscopy

Literature procedure for hydroxyl end group analysis by ^{31}P {^{1}H} NMR spectroscopy was followed.[3] A mixture of stock solution (40 µL), excess 2-chloro-4,4,5,5-tetramethyl dioxaphospholane (40 µL) and polymer sample (20 mg) was added to a NMR tube and shaken. The mixture was allowed to react for 6 h before measurement. The stock solution consists of Bisphenol A (400 mg) and of $Cr(acac)_3$ (5.5 mg) in pyridine (10 mL).

Ring-opening polymerization of DL

Under anaerobic conditions, LO (3.3 mL, 20.6 mmol), **1** (10.0 mg, 0.01 mmol), BDM (5.6 mg, 0.04 mmol) and ε-DL (1.7 mL, 10.3 mmol) were placed in a vial and heated to 60 °C with stirring for 5 min (molar ratio: [**1**]/[BDM]/[ε-DL]/[LO] = 1/4/1000/2000). After the desired reaction time, the solution became viscous and the mixture was exposed to air to quench the polymerization. The crude sample was analysed by ^{1}H NMR spectroscopy. SEC analysis (THF as eluent, 1 mL/min, 30 °C) of the crude polymer was carried out following removal of excess solvent under reduced pressure. The polymer was purified by precipitation of a DCM solution (<1 g/mL) into MeOH (50 mL) at -78 °C.

Exemplar ring-opening copolymerization of LO and TCA

Under anaerobic conditions, LO (3.3 mL, 20.6 mmol), [$LMgZn_2(C_6F_5)_2$] (10.0 mg, 0.01 mmol), BDM (5.6 mg, 0.04 mmol) and chosen TCA were placed in a vial and heated to 140 °C with stirring (molar ratio: [**1**]/[BDM]/TCA/LO = 1/4/100/300). Thereafter, the mixture was exposed to air to quench the polymerization. The crude sample was analysed by ^{1}H NMR spectroscopy. SEC analysis (THF as eluent, 1 mL/min, 30 °C) of the crude polymer was carried out following removal of excess LO under reduced pressure. The polymer was purified by precipitation of a DCM solution (<1 g/mL) into MeOH (50 mL). Note: Interestingly, the methine proton in the LO unit on the backbone of the polyester was observed to be a multiplet from 4.78 ppm to 5.18 ppm which is a feature not observed in previous reports of polymers utilizing LO.[4] Nevertheless, HSQC NMR spectroscopy confirmed these peaks correlated to carbon atoms in very similar environments, thus suggesting these peaks are likely the due the same proton (Fig. S7).

Exemplar terpolymerization of DL, LO and TCA

Under anaerobic conditions, LO (3.3 mL, 20.6 mmol), **1** (10.0 mg, 0.01 mmol), BDM (5.6 mg, 0.04 mmol) and DL were added to a vial charged with a stirring bar and allowed to react at 60 °C for a desired amount of time. After an aliquot was taken, the selected TCA was added and the mixture was allowed to react further at 140 °C until complete conversion of the anhydride (see table for molar ratios). Thereafter, the mixture was exposed to air to quench the

polymerization. ^1H NMR spectroscopy and SEC analysis (THF as eluent, 1 mL/min, 30 °C) were performed at key intervals. The polymer was purified by precipitation of a DCM solution (<1 g/mL) into MeOH (50 mL) three times.

Note: $M_{n,\ NMR}$ and the $Wt\%_{Hard}$ are calculated by examining the degree of polymerization (DP) based on a comparison between the initiator signals and the peaks for the PE and PDL blocks. For instance, for the triblock copolyester prepared using TCA 1 with 41 wt% hard block, the integration of the benzylic protons of the initiator (proton 'a' (7.34 ppm) in Fig. S12) is compared with the methylene protons on the anhydride unit (proton '20' (5.74 ppm) in Fig. S12) and the methine proton on the lactone unit (proton '5' (4.85 ppm) in Fig. S12). A DP of 10 and 33 for the PE and PDL blocks are found, respectively. As each PE and PDL unit is 386.53 g mol^{-1} and 170.25 g mol^{-1}, this amounts to 37.9 kg mol^{-1} for the block copolyester. Similarly, the wt% is calculated by comparing the total molecular weight for the PE and the PDL and converting the ratio for PE into a percentage. The theoretical renewable content of the block copolymer is calculated by dividing the total mass of the renewable monomers (i.e. limonene oxide, ε-decalactone, α-phellandrene and citraconic anhydride) by the total mass of all the monomers used in the reaction.

Table S1. Block copolyester synthesis

Sample	TCA used	[DL]	[TCA]	DL conv. (%)	TCA conv. (%)	ROP time (min)	ROCOP time (h)
1	TCA 1	1200	75	65	>99	8	32
2	TCA 1	1000	125	58	>99	6	48
3	TCA 1	1000	175	52	>99	6	67
4	TCA 1	800	225	58	>99	5	88
5	TCA 2	1000	175	50	>99	6	49
6	TCA 3	1000	175	50	>99	6	108
7	TCA 4	1000	175	50	>99	6	138

Molar ratio: [cat.]/[BDM]/[LO] = 1/4/2000

Degradation experiments

A modified literature procedure was followed.[5] *p*TSA.H_2O (10 mg, 0.05 mmol) was added to a solution of P3 (100 mg) in THF (3 mL) and heated with stirring to 60 °C in a vial. Progress was monitored by SEC analysis of aliquots taken at various time points in order to track the degradation progress.

1.3. Supporting schemes, figure and tables

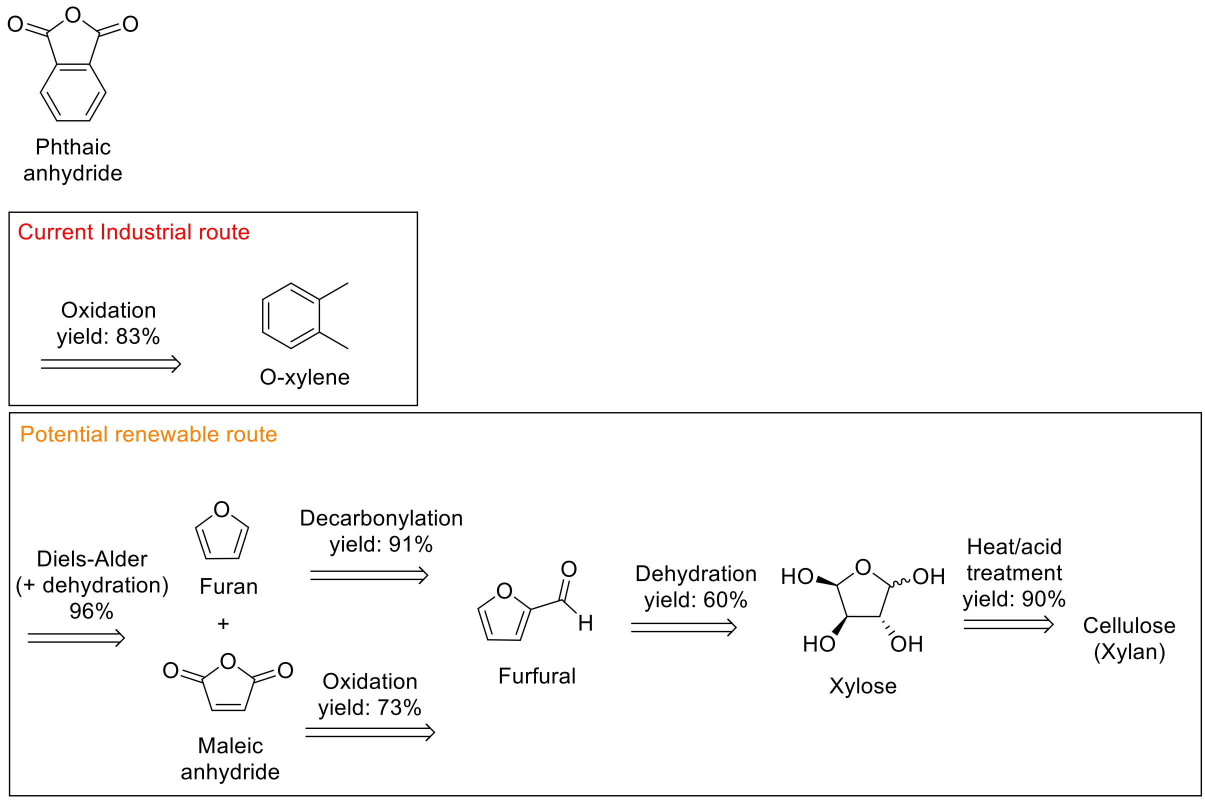

Scheme S1. Industrial and potential renewable routes to phthalic anhydride.[6]

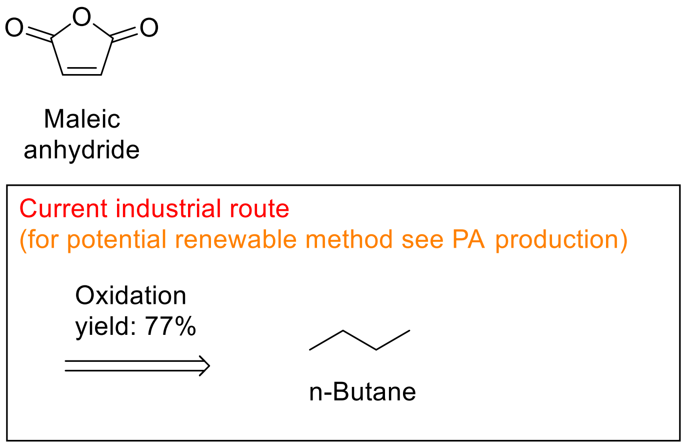

Scheme S2. Industrial and potential renewable routes to maleic anhydride.[7]

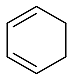

1,3-cyclohexadiene

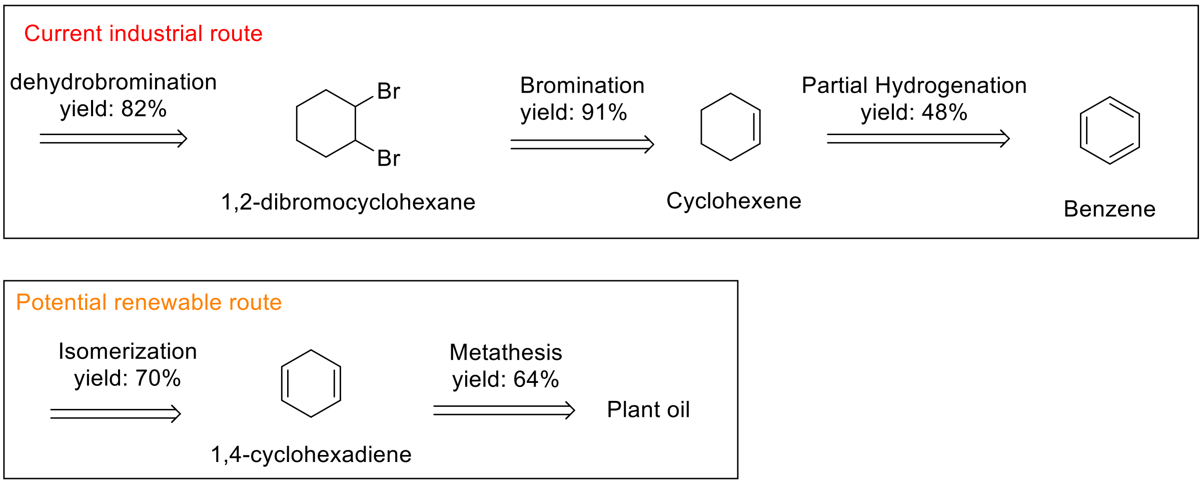

Scheme S3. Industrial and potential renewable routes to 1,3-cyclohexadiene.[8]

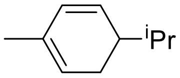

α-phellandrene

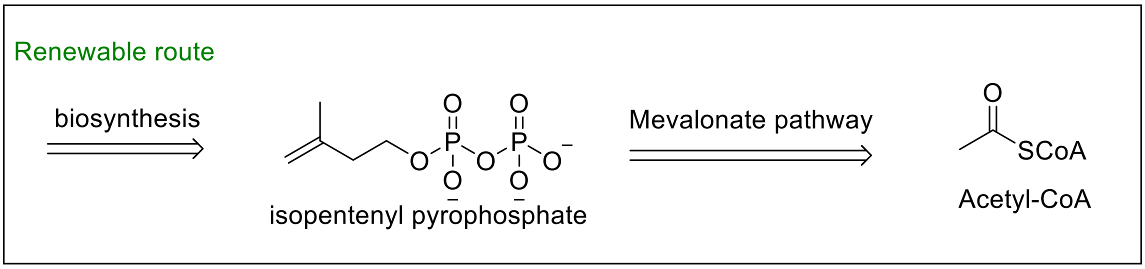

Scheme S4. Renewable routes to α-phellandrene.[2]

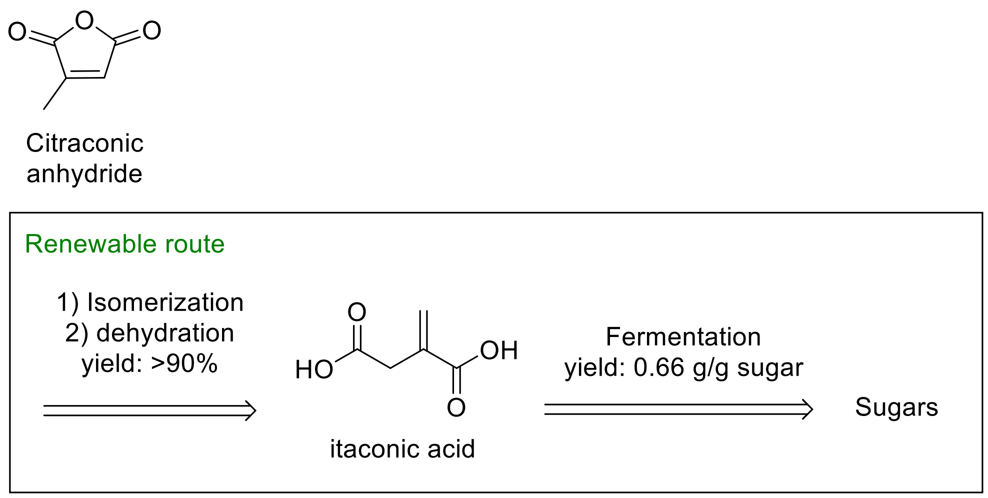

Scheme S5. Renewable routes to citraconic anhydride.[2,9]

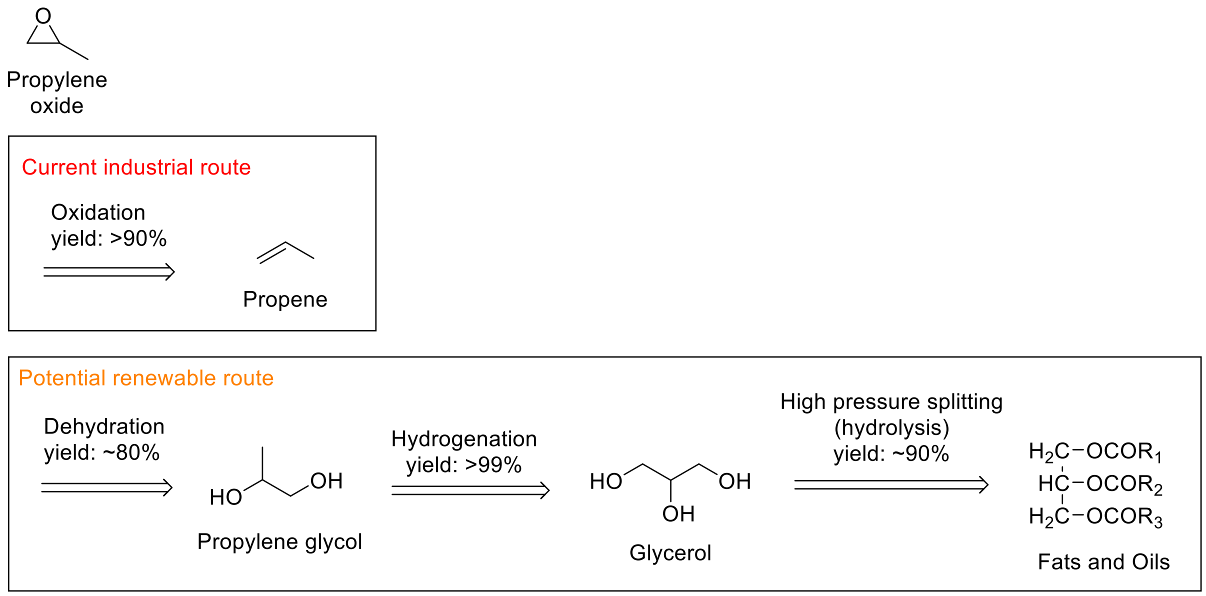

Scheme S6. Industrial and potential renewable routes to propylene oxide.[10]

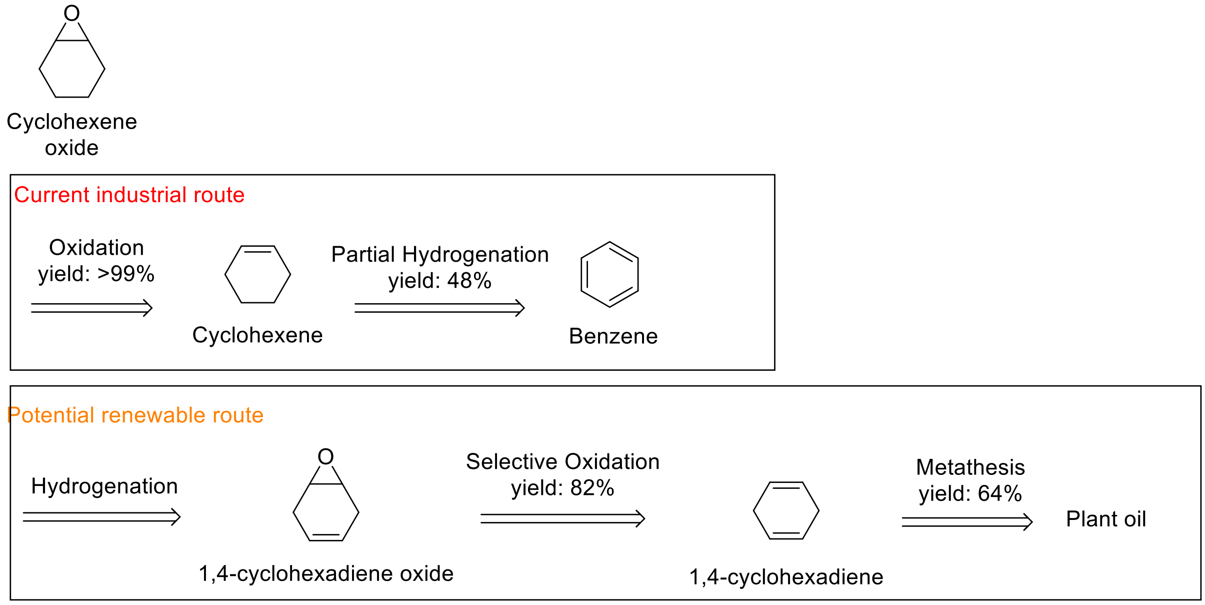

Scheme S7. Industrial and potential renewable routes to cyclohexene oxide.[11]

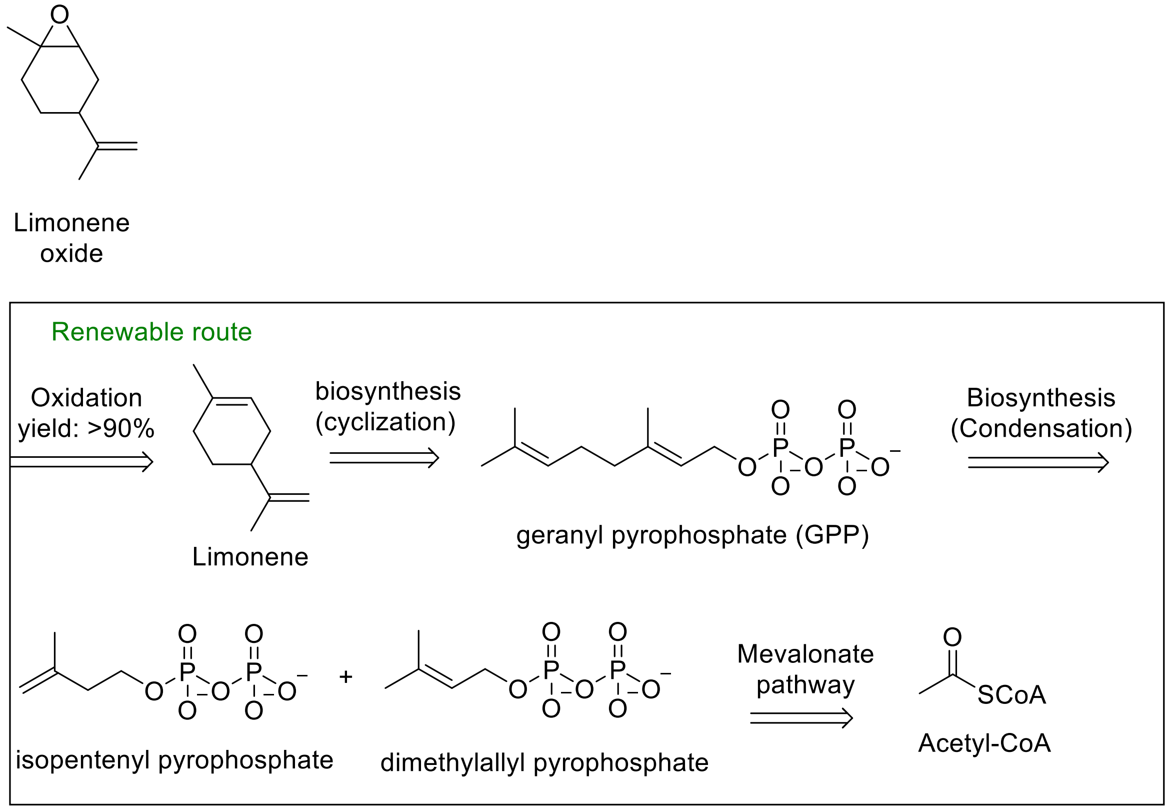

Scheme S8. Renewable routes to limonene oxide.[12]

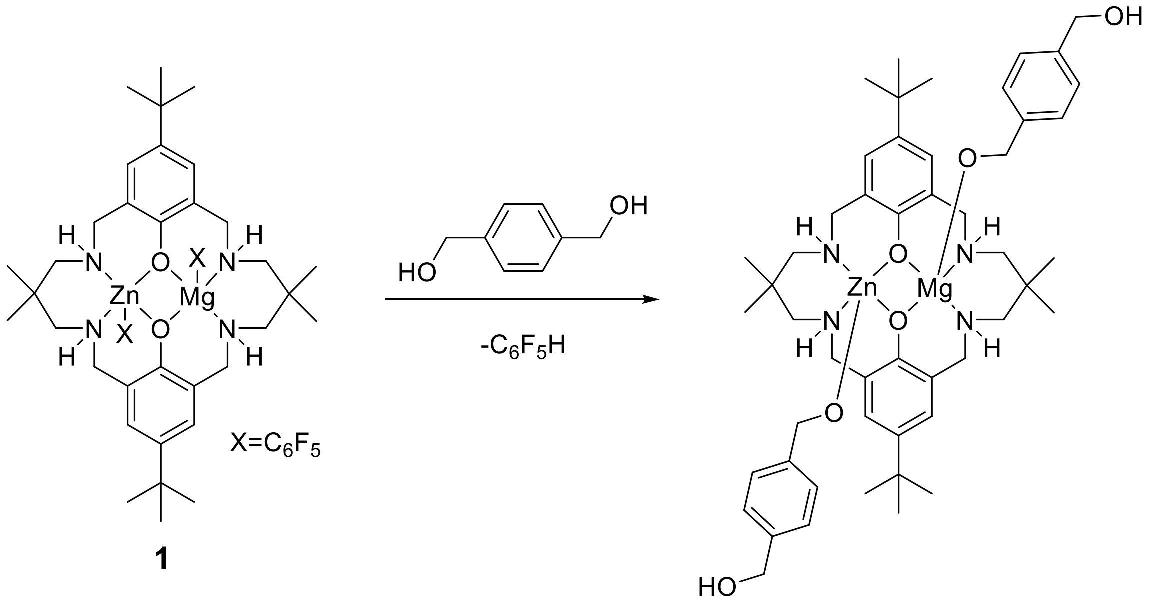

Scheme S9. In situ activation of the catalyst from reaction between 1 and 1,4-Benzenedimethanol (BDM)

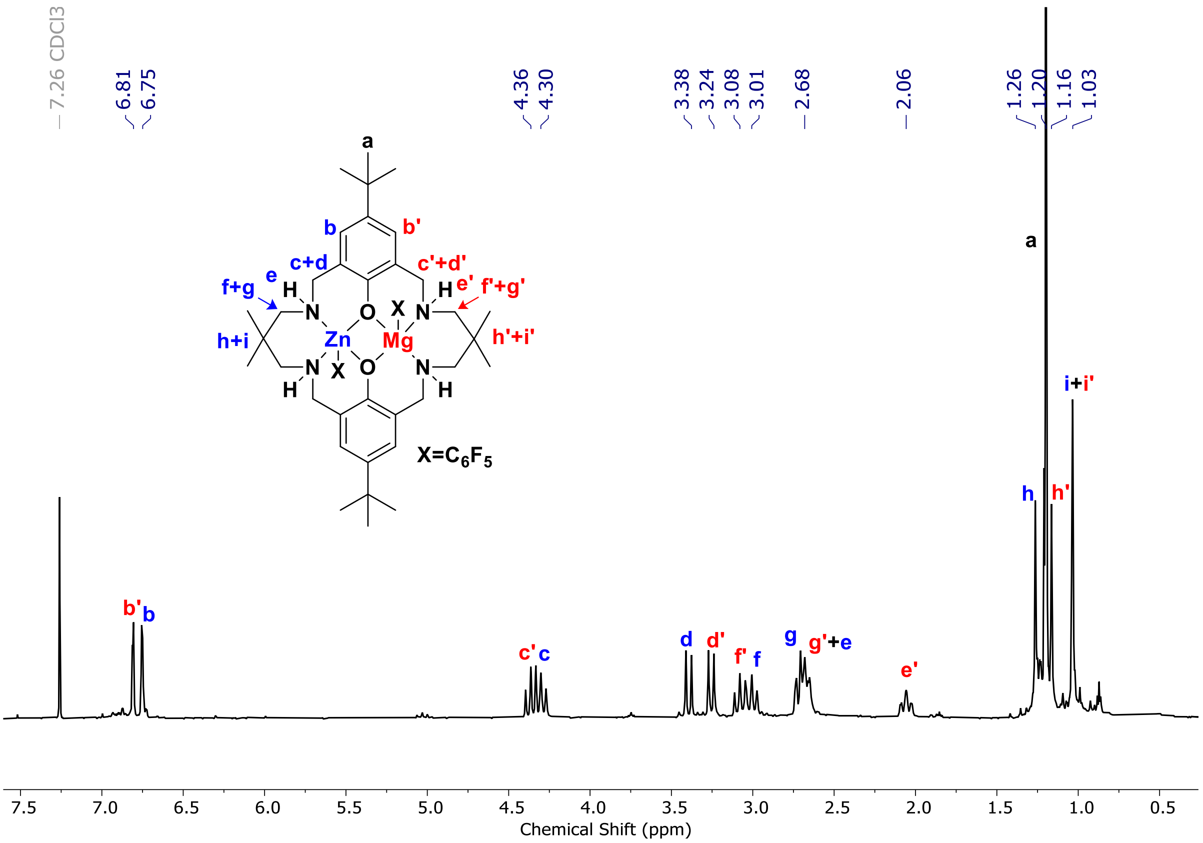

Figure S1. ^{1}H NMR spectrum of $[LMgZn(C_6F_5)_2]$, **1**, in $CDCl_3$ at 298 K.

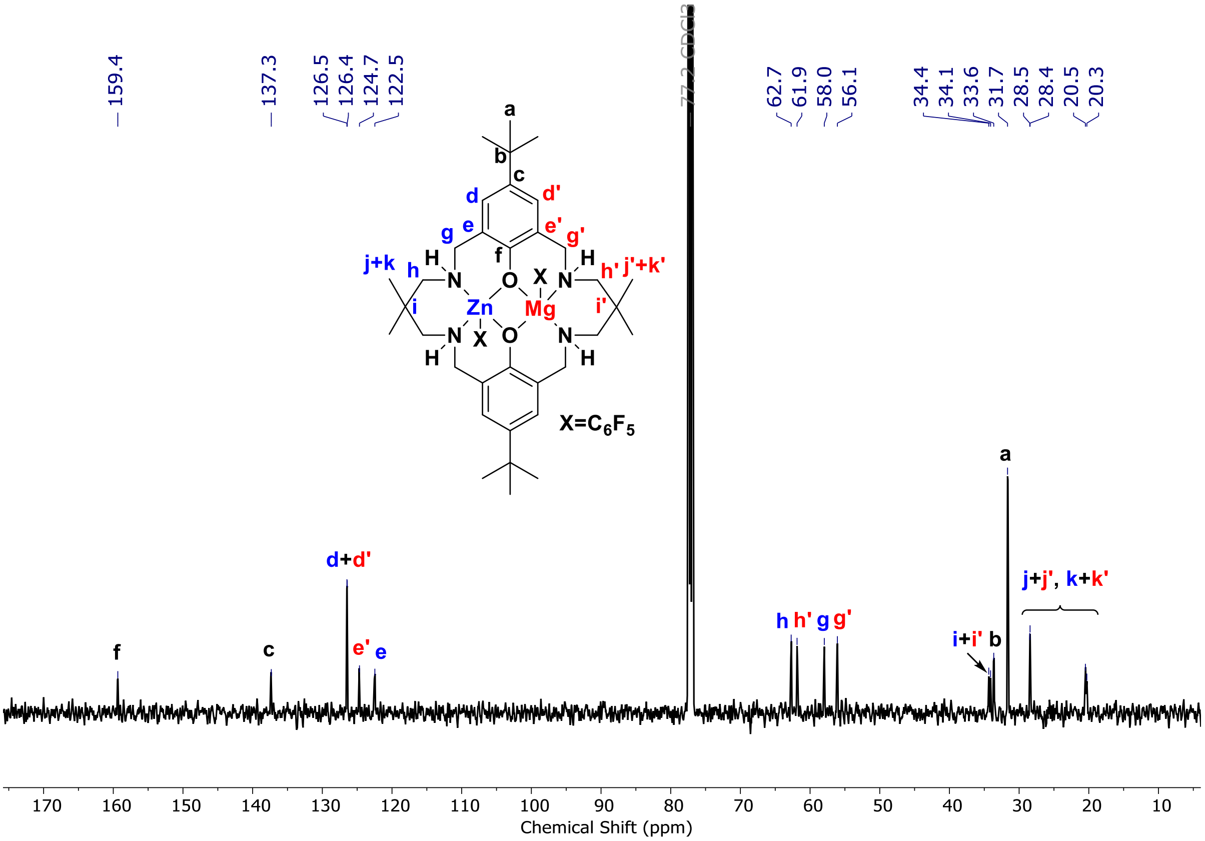

Figure S2. $^{13}C\{^{1}H\}$ NMR spectrum of [LMgZn(C_6F_5)$_2$], **1**, in $CDCl_3$ at 298 K.

Table S2. ROCOP of LO and 4 different TCA

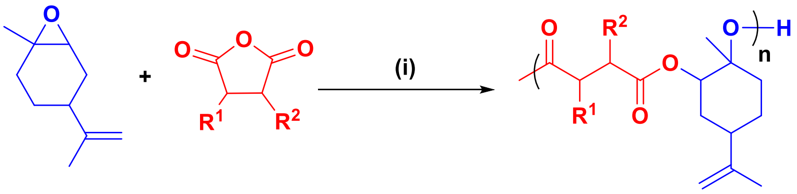

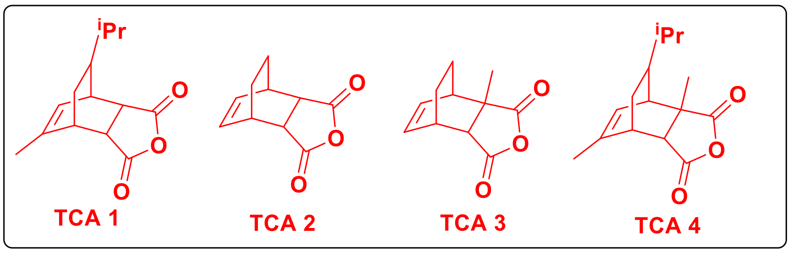

#	TCA	Time (h)	Conv. (%)[c]	Selectivity (%)[d]	M_n (kg mol^{-1})[e]	*Đ*[e]	T_g (°C)[f]	$T_{d,5}$ (°C)[g]
1	1	22	>99	>99	6.3	1.19	93	259
2[a]	1	14	>99	>99	7.0	1.18	86	258
3[b]	1	34	0	-	-	-	-	-
4	2	11	>99	>99	5.4	1.19	102	266
5	3	53	>99	>99	5.9	1.15	82	264
6	4	75	>99	>99	5.3	1.18	88	260

Reaction conditions: (i): [**1**]/[BDM]/[TCA]/[LO] = 1/4/100/300, 140 °C. [a]*trans*-limonene oxide used. [b]*cis*-limonene oxide used. [c]Conversions obtained from ^{1}H NMR spectra by comparing the monomer and polymer peaks at 5.76-5.77 and 5.68 ppm, respectively. [d]Selectivity of polyester vs. polyether, determined from ^{1}H NMR spectra by comparing main chain signals to signals in the polyether region (3.0-3.5 ppm). [e]M_n and *Đ* measured by SEC (calibrated using polystyrene standards). [f]Determined by DSC. [g]Decomposition temperature (at 5% decomposed mass) determined by TGA.

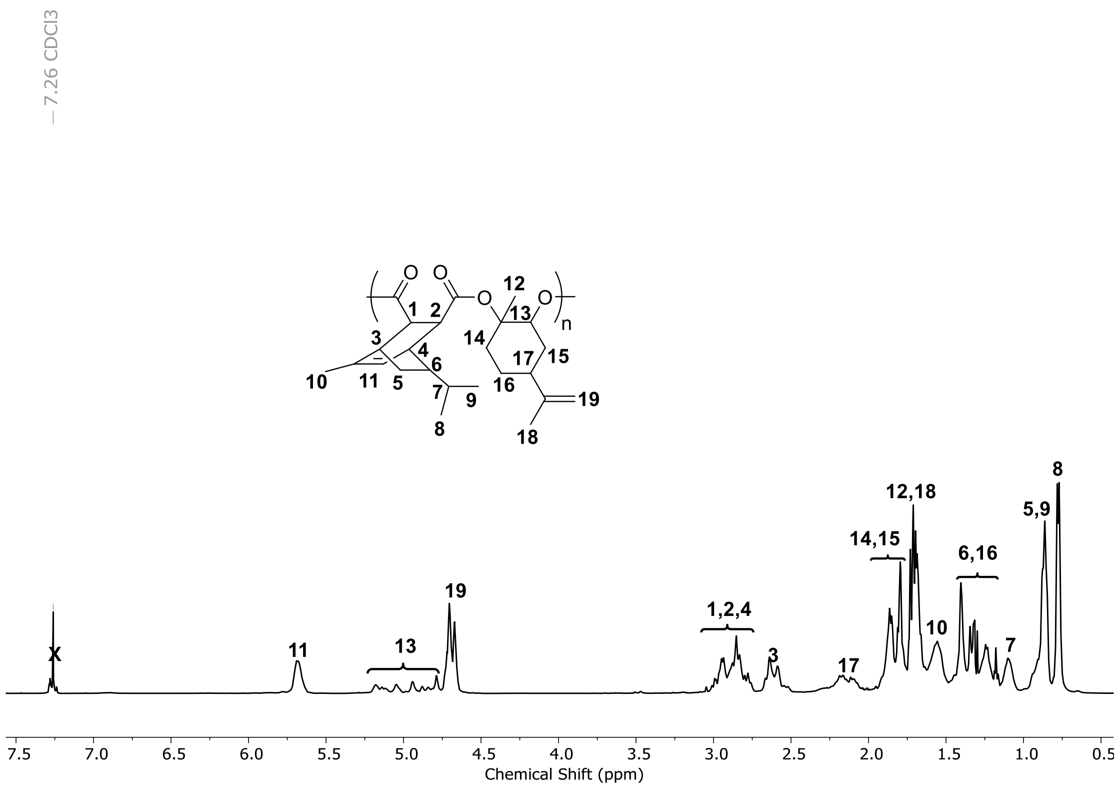

Figure S3. ^{1}H NMR spectrum of the polyester from the ROCOP of LO/TCA 1 in $CDCl_3$.
Note: The polyester LO methine proton was observed as a multiplet (4.78 - 5.18 ppm), which is a feature different to previous polymers utilizing LO. [4] The multiplet may have arisen due to a lack of regioselectivity in the ROCOP process. The HSQC NMR spectrum confirms these peaks correlate to carbon atoms in very similar environments, thus suggesting these peaks are likely the due the same proton (Fig. S7). As such, the multiplets are proposed to be due to regio-irregularity in the ROCOP process.

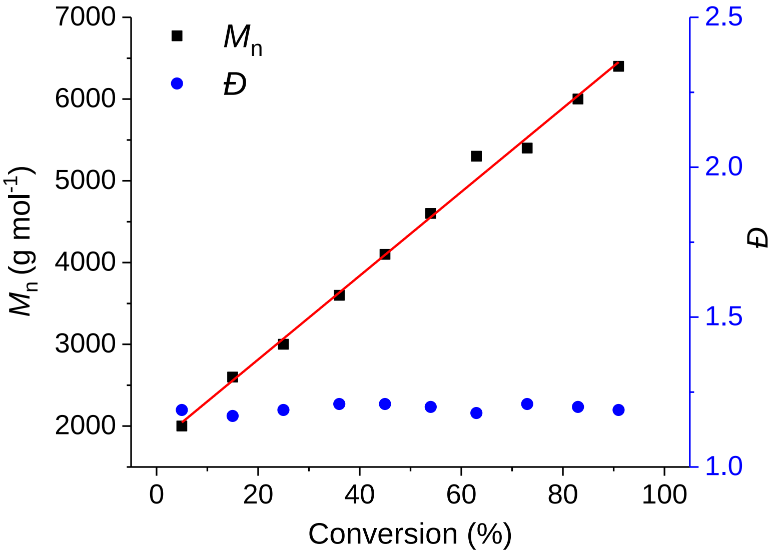

Figure S4. M_n and $Đ$ vs. conversion for the ROCOP of LO/TCA 1

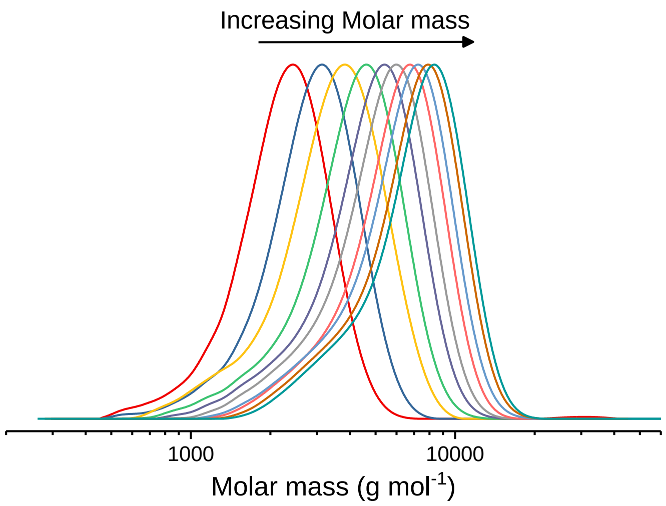

Figure S5. SEC traces of aliquots obtained in thee ROCOP of LO/TCA 1
Note: aliquots taken between 5% and 90% conv.

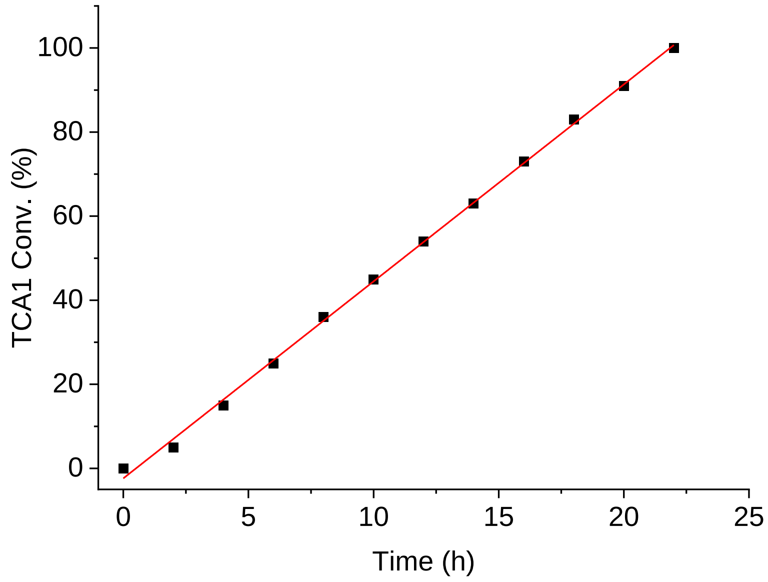

Figure S6. Conversion vs. time for the ROCOP of LO/TCA 1

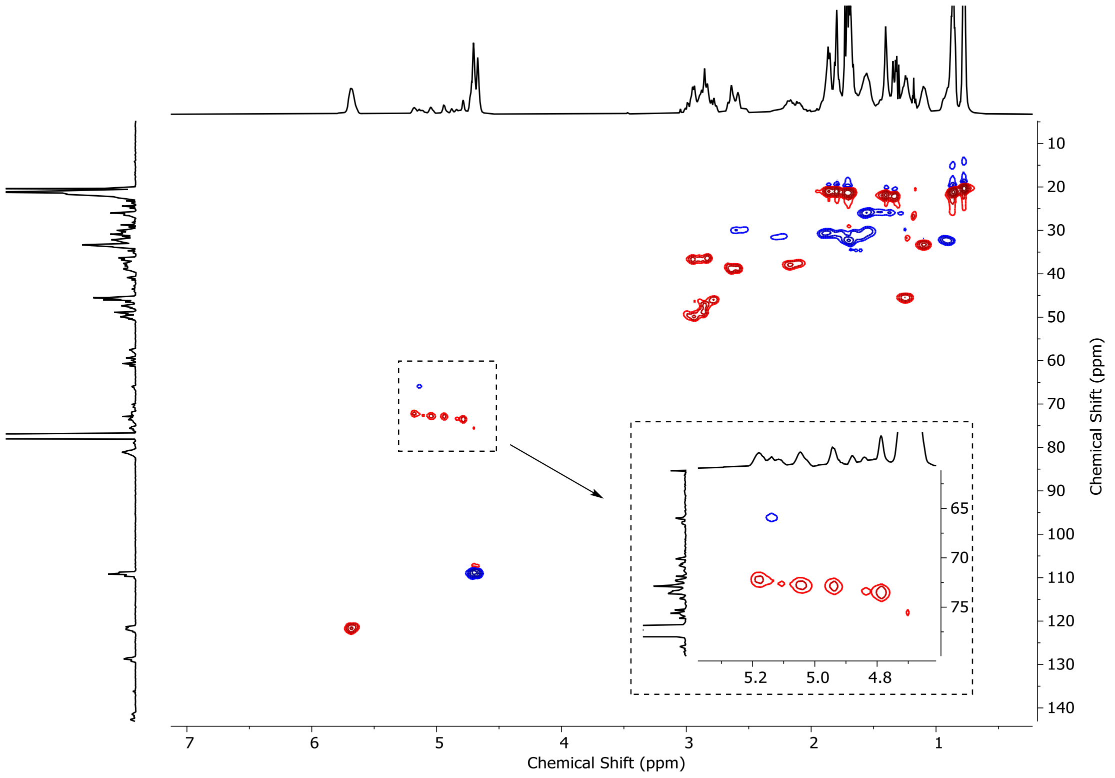

Figure S7. HSQC NMR spectrum of the polyester from the ROCOP of LO/TCA 1 in $CDCl_3$. Insert: selected expanded region illustrating multiplet splitting between 4.78-5.18 ppm correlating to very similar carbon environments.

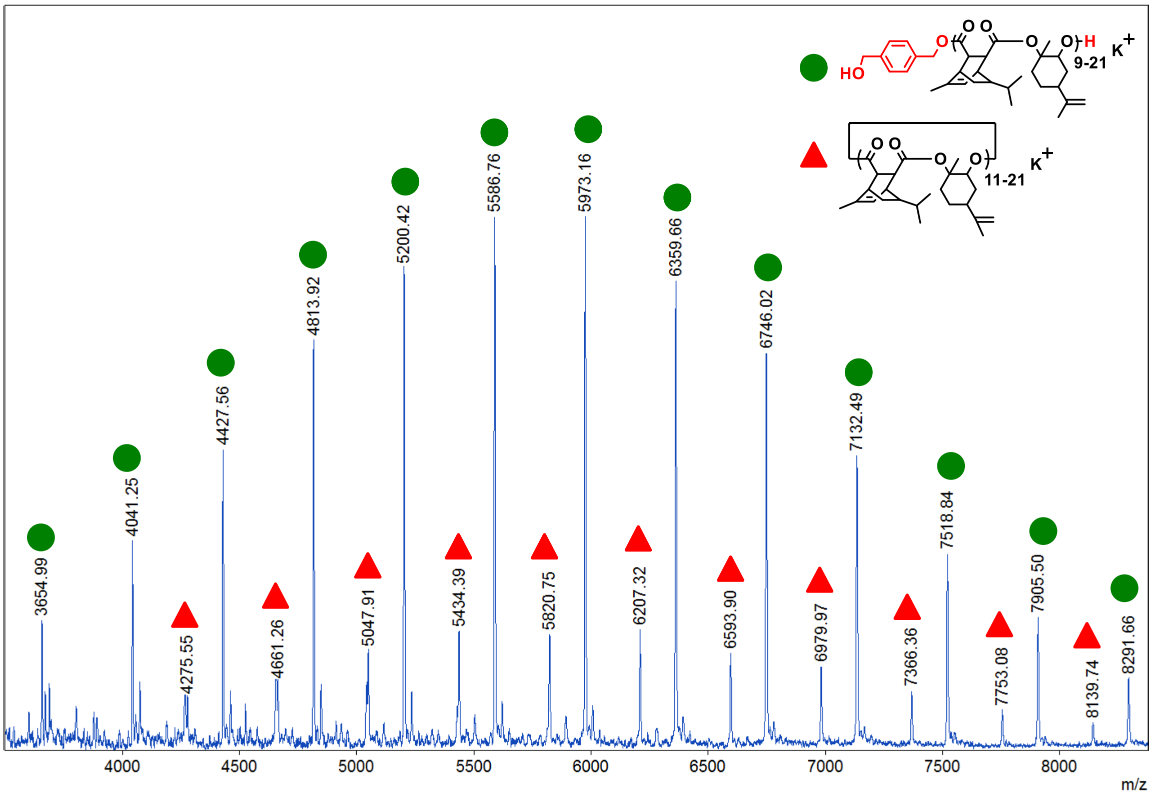

Figure S8. Representative MALDI-ToF of the polyester from the ROCOP of LO/TCA1.

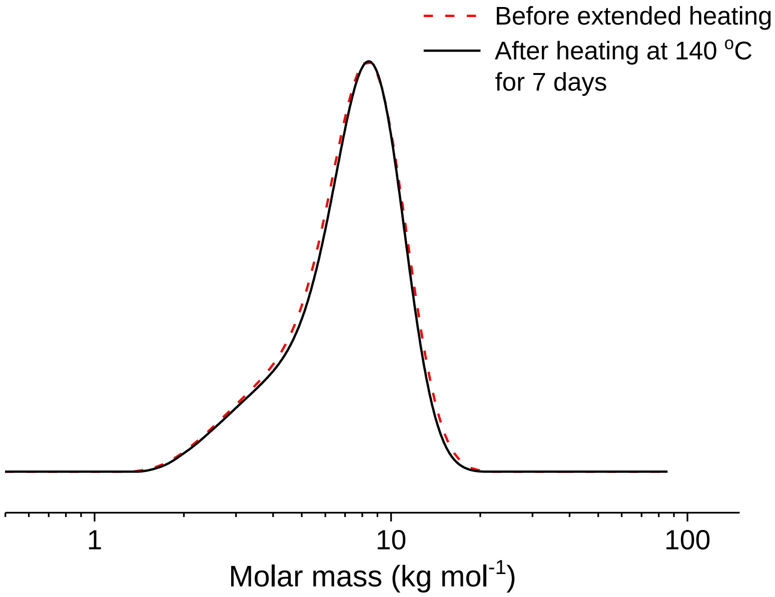

Figure S9. SEC trace for the ROCOP of LO/TCA 1 after deliberate heating at 140 °C for 7 days (after full conversion).

Note: M_n and $Đ$ remained the same after 7 additional days of heating, indicating limited transesterification.

Table S3. ROP of DL

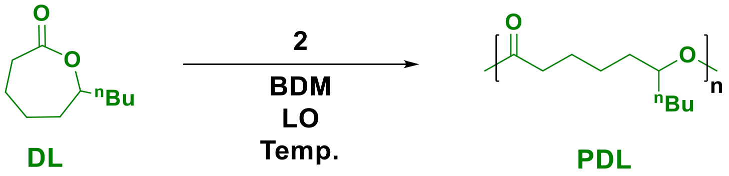

#	[2]/[BDM]/[DL]/[LO]	Temp (°C)	Time (min)	Conv. (%)[a]	TOF (h^{-1})[b]	M_n (kg mol^{-1})[c]	$Đ$[c]
1	1/4/500/500	25	15	25	500	7.5	1.08
2	1/4/500/500	40	5	25	1500	7.2	1.11
3	1/4/1000/1000	60	5	38	4560	22.9	1.08
4	1/4/1000/1000	80	5	57	6840	31.0	1.11
5	1/4/1000/1000	80	7	74	6340	37.6	1.32

[a]Conversions obtained from ^{1}H NMR spectra by comparing the monomer and polymer peaks at 4.15 ppm and 4.85 ppm, respectively. [b]Turn over frequency (TOF) is calculated based on number of mol of monomer converted/mol of catalyst/time. [c]M_n and $Đ$ measured by SEC (THF as eluent, 1 mL/min, 30 °C, calibrated using polystyrene standards).

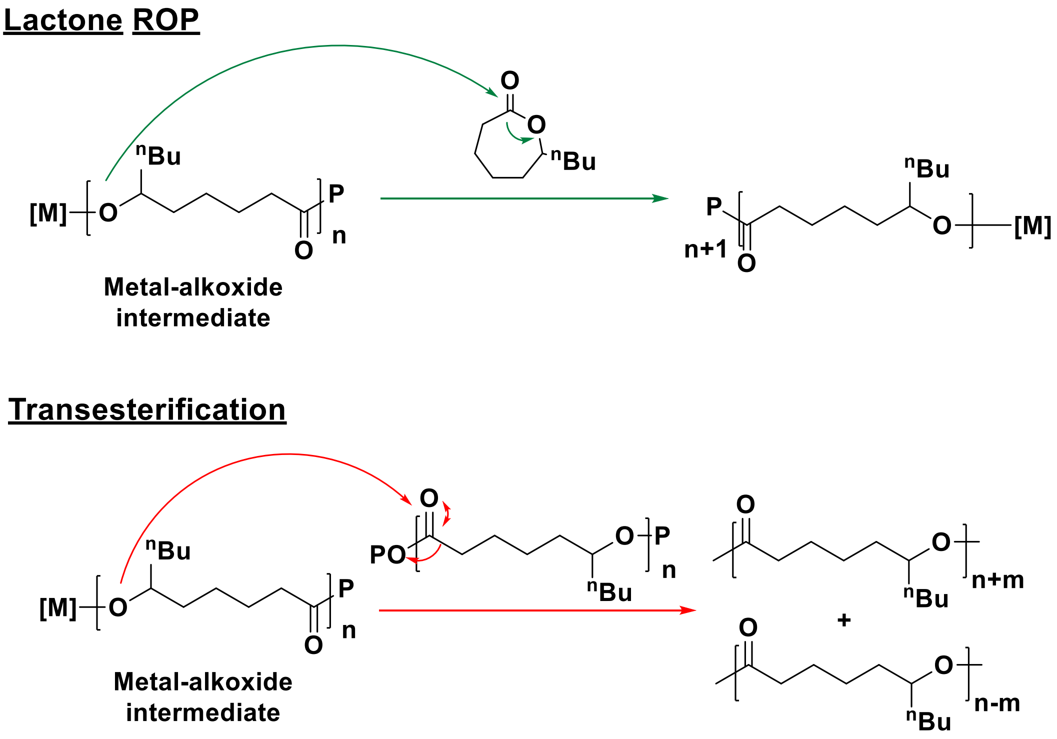

Figure S10 Illustration of the DL ROP and transesterification mechanisms.
The latter may have become competitive at high DL conversion as the monomer concentration drops (note: P = polymer chain. n and m indicate number of repeat units and n≠ m and n > m).

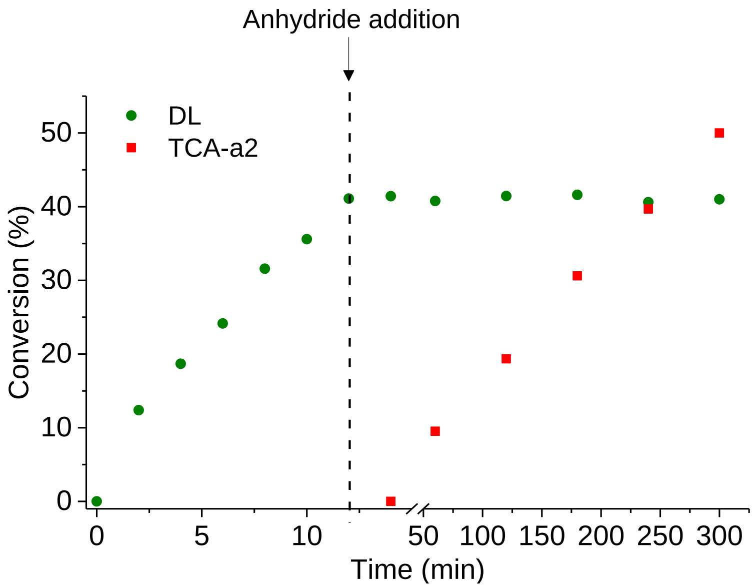

Figure S11. Conversion vs. time plot for the selectivity study between ROP of DL and ROCOP of LO/TCA 1.

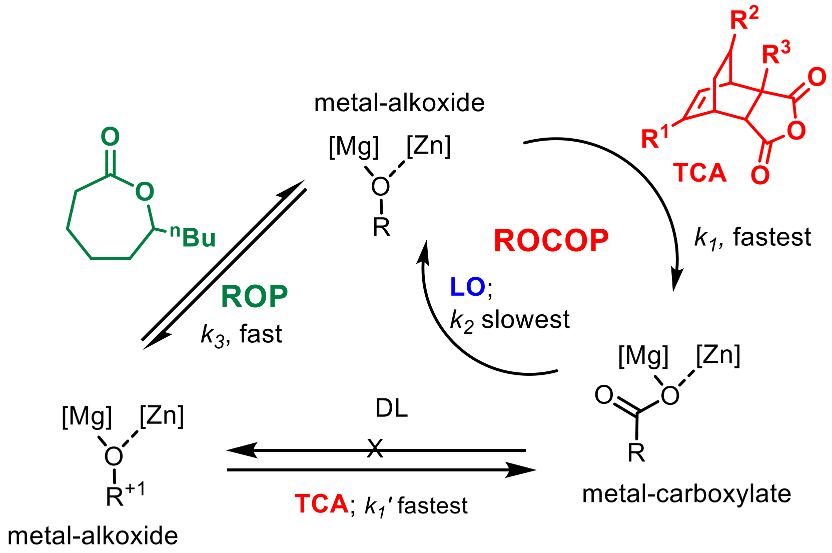

Figure S12. Proposed 'switching' mechanism for ROCOP of LO/TCA and ROP of DL using a single catalyst

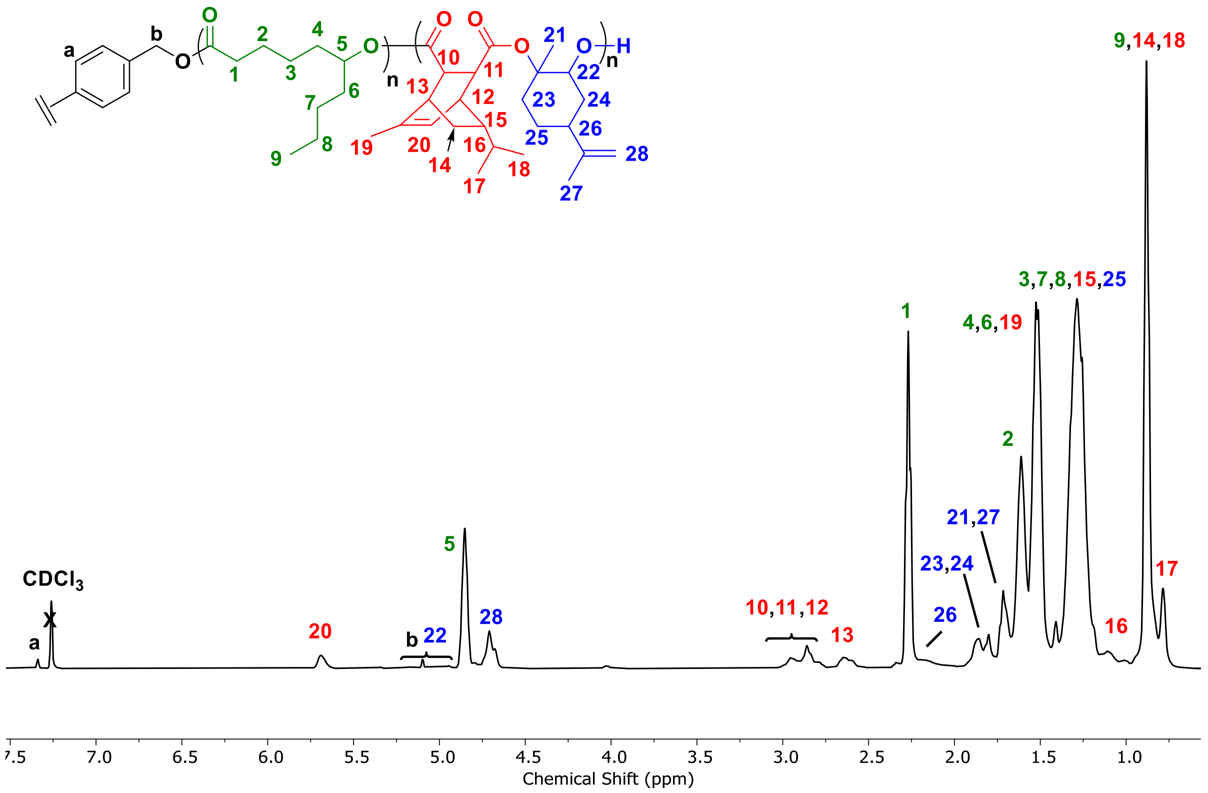

Figure S13. ^{1}H NMR spectrum of P3 (in $CDCl_3$).

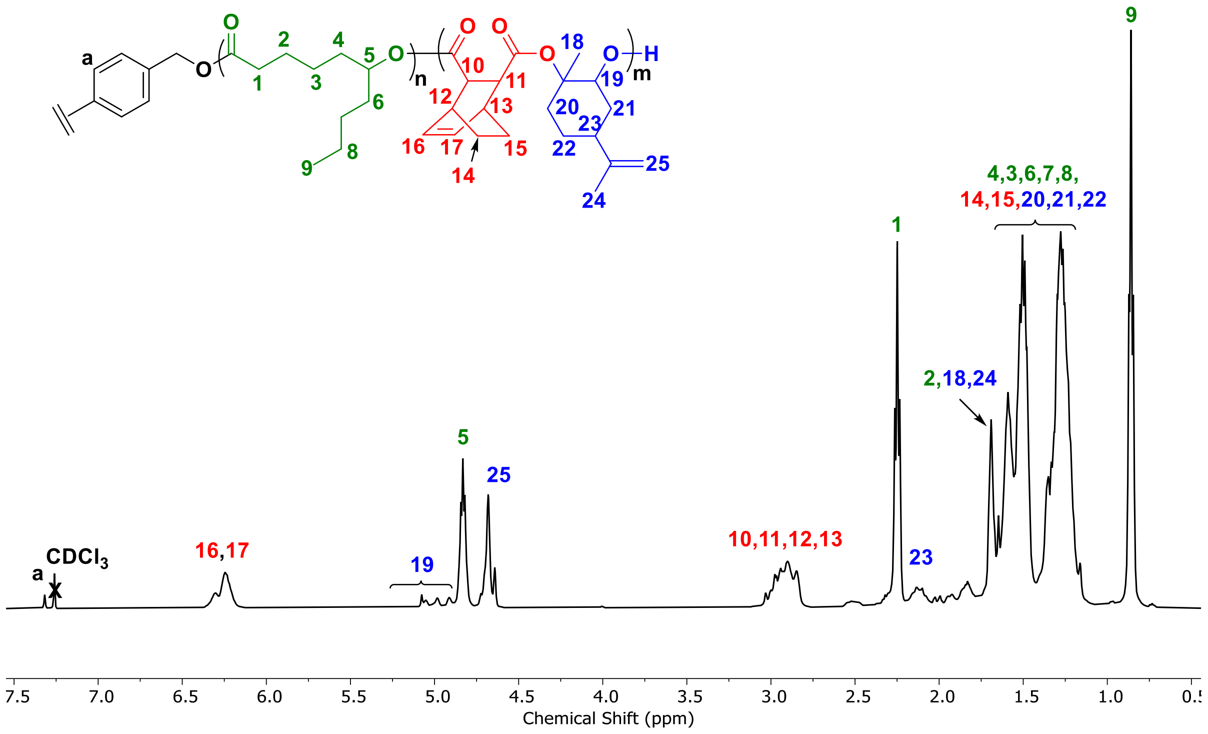

Figure S14. ^{1}H NMR spectrum of P5 (in $CDCl_3$).

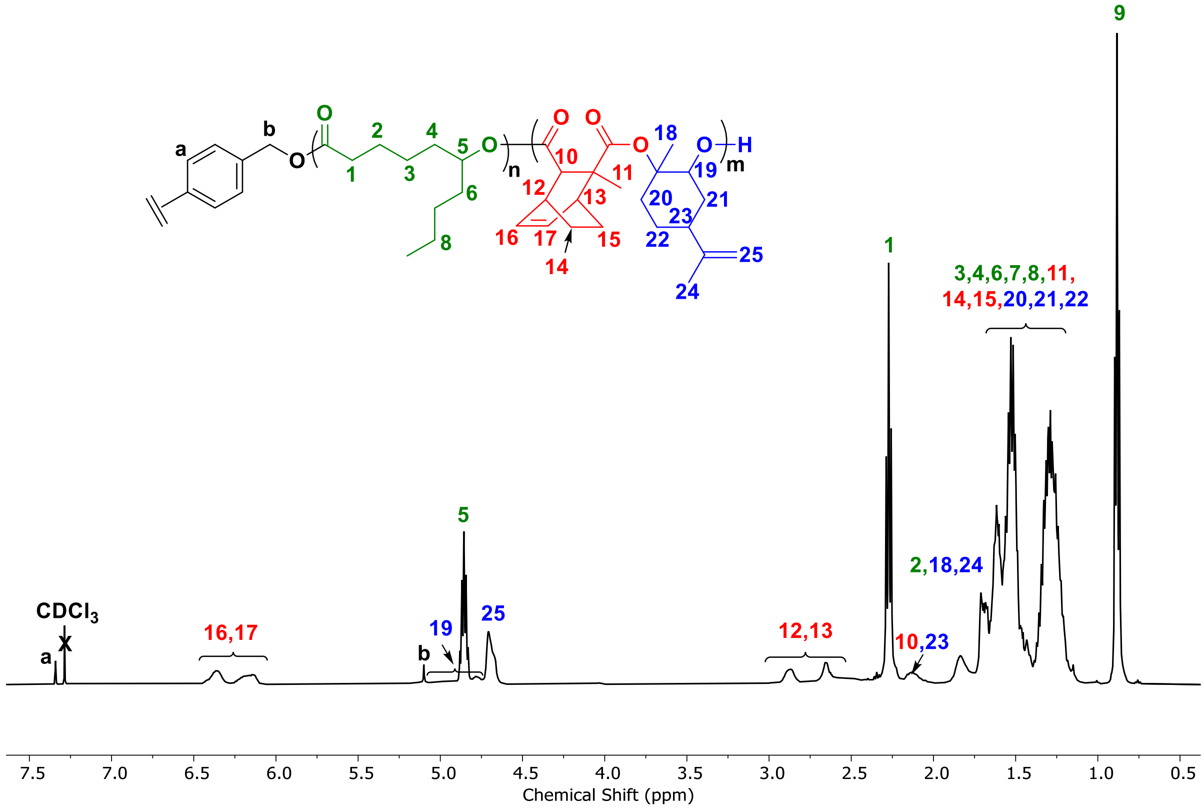

Figure S15. ^{1}H NMR spectrum of P6 (in $CDCl_3$).

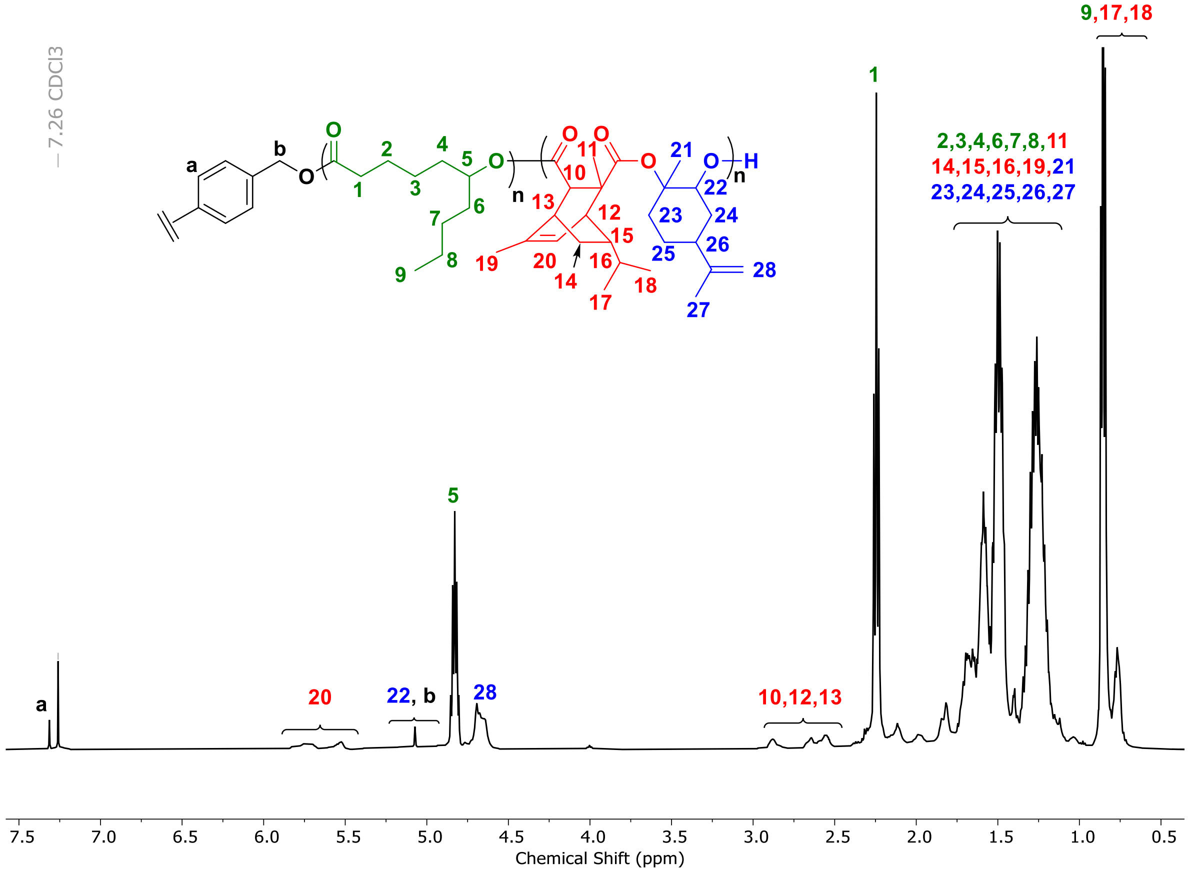

Figure S16. ^{1}H NMR spectrum of P7 in ($CDCl_3$).

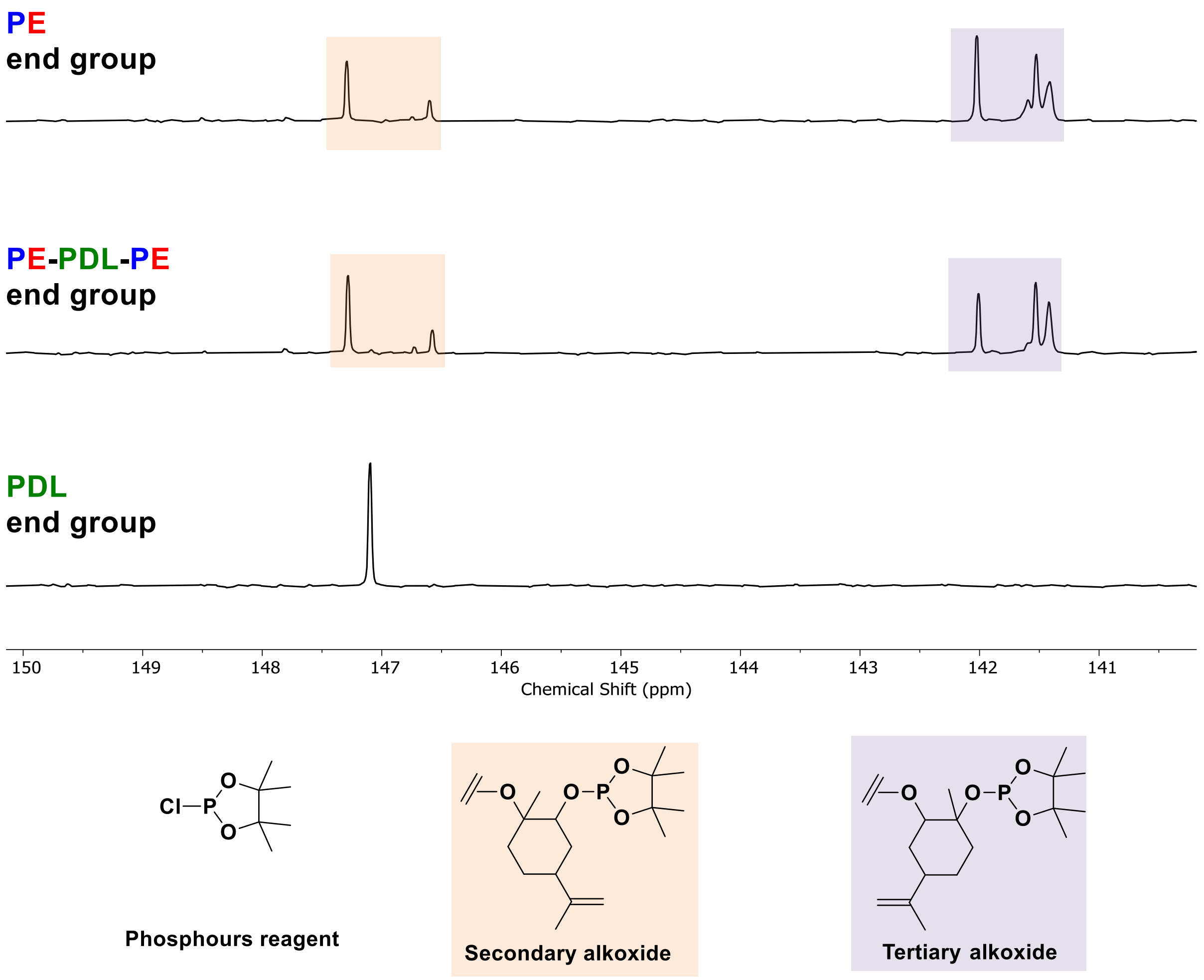

Figure S17. Selected region of $^{31}P\{^{1}H\}$ NMR spectra of P3 hydroxyl polymers showing a complete change in end group resonance (in $CDCl_3$).

Note: Two groups of peaks are observed for LO/TCA ROCOP polyesters as LO can be terminated as both secondary and tertiary hydroxyls and the polymerization is regio- and stereoirregular.[3]

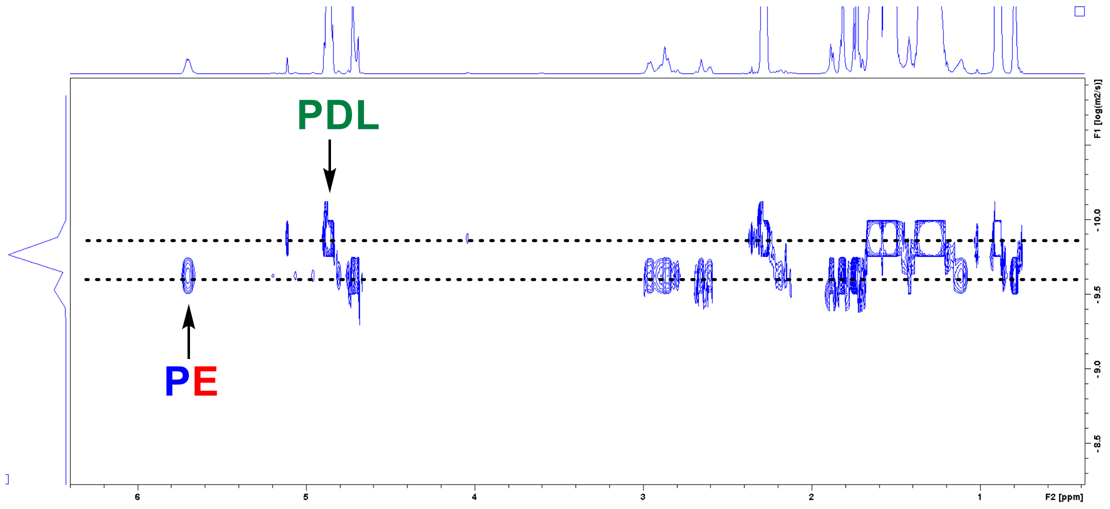

Figure S18. Homopolymer blend with similar molar masses to the respective blocks in P3 showing two different coefficients in $CDCl_3$.

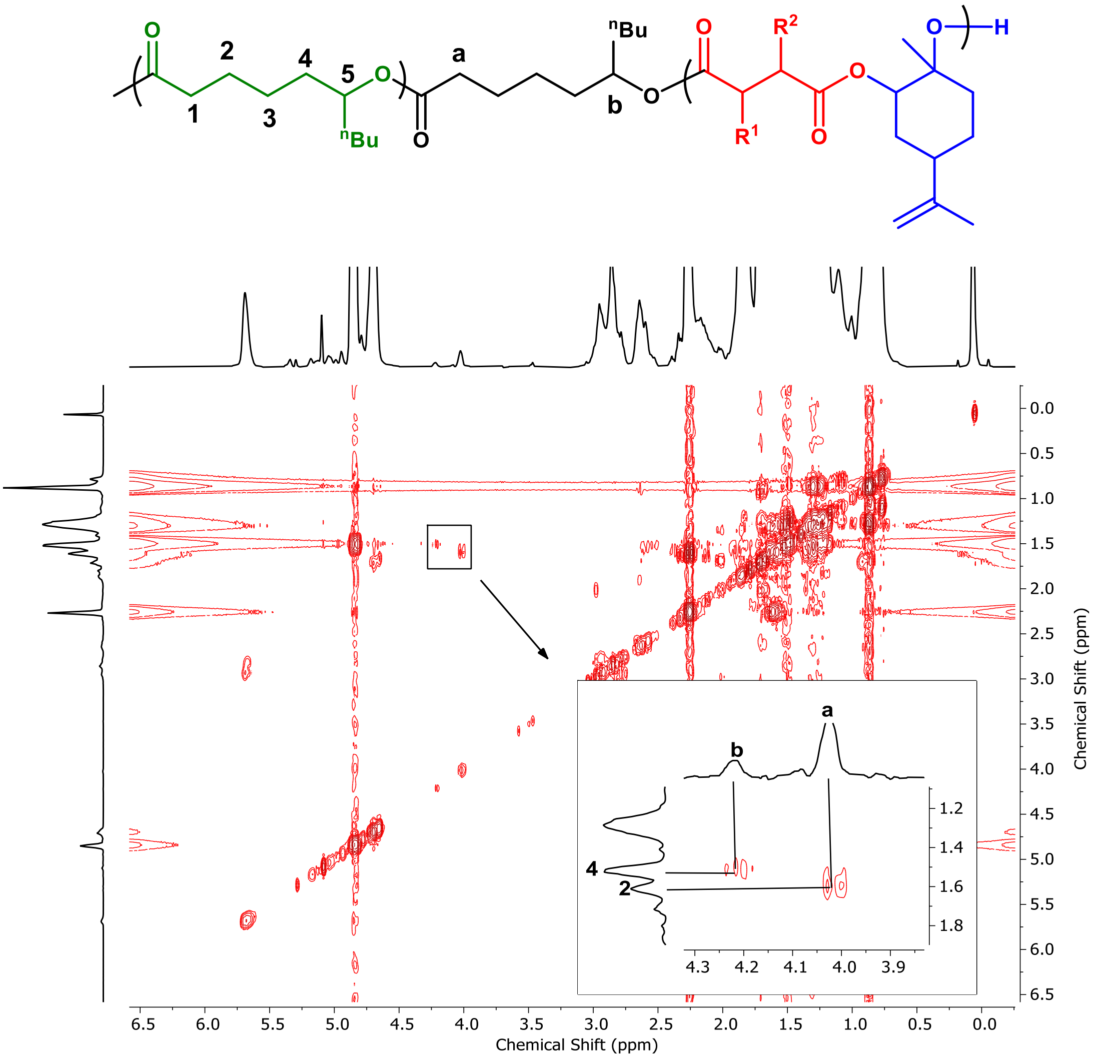

Figure S19. COSY NMR spectrum of P3 in $CDCl_3$.
Insert: Expanded region showing correlation between the junction unit resonances and the main chain unit.

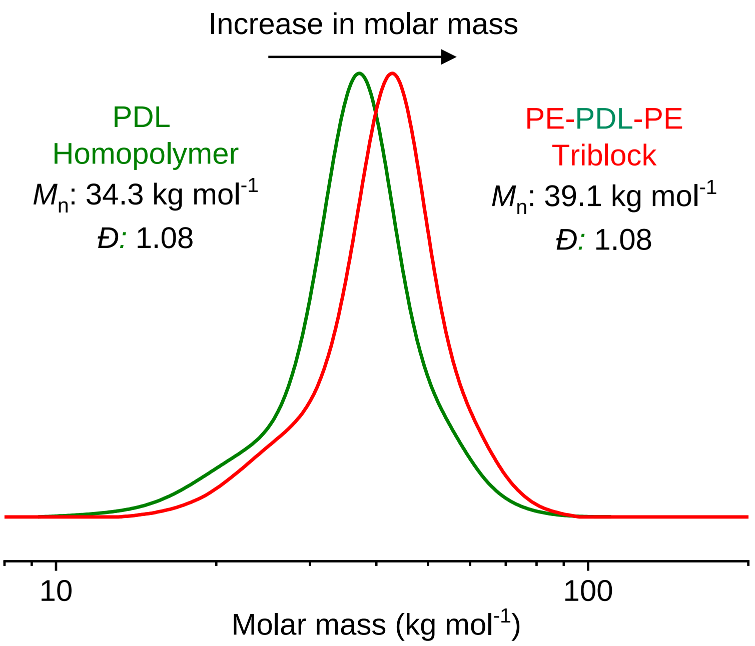

Figure S20 Stacked SEC traces of P1 illustrating an increase molar mass with retention of very narrow *Đ* (in THF at 30 °C, calibrated using polystyrene standards).

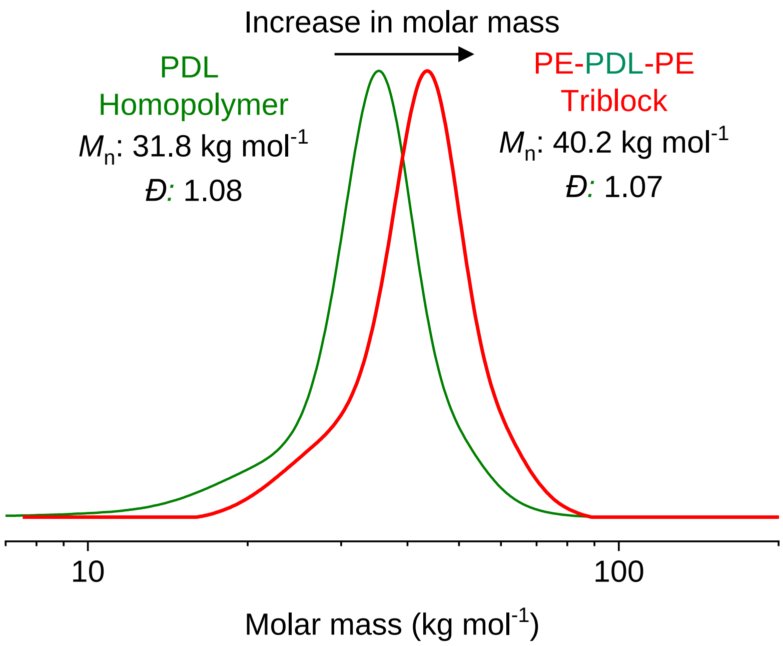

Figure S21 Stacked SEC traces of P2 illustrating an increase molar mass with retention of very narrow *Đ* (in THF at 30 °C, calibrated using polystyrene standards).

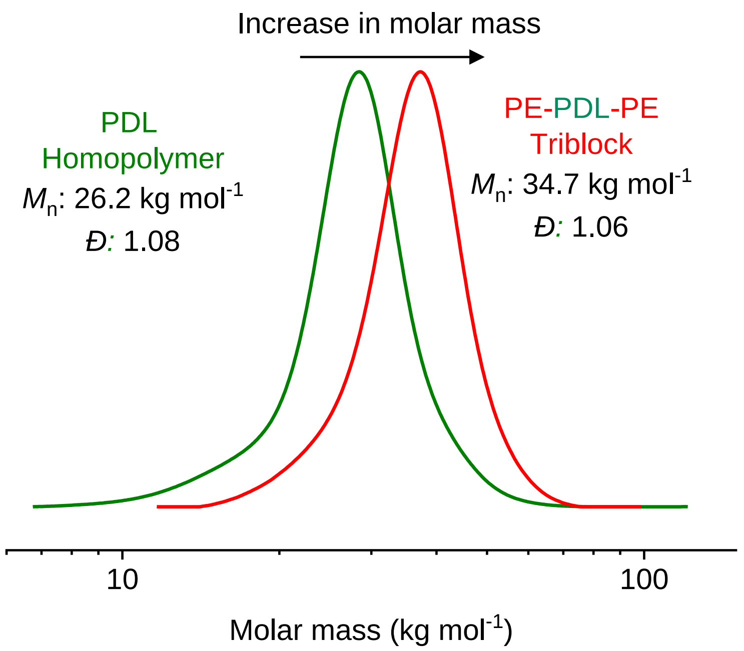

Figure S22 Stacked SEC traces of P4 illustrating an increase molar mass with retention of very narrow *Đ* (in THF at 30 °C, calibrated using polystyrene standards).

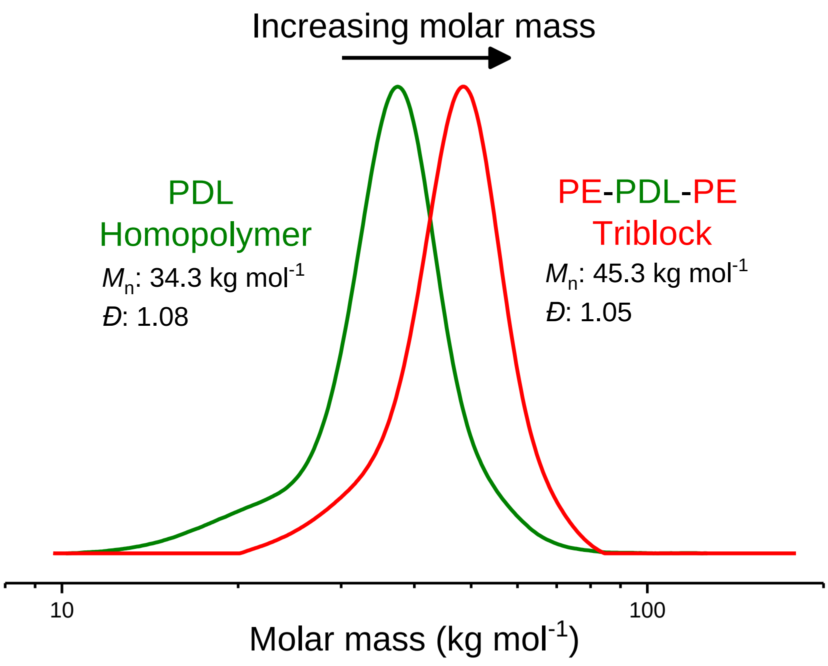

Figure S23. Stacked SEC traces of P5 illustrating an increase molar mass with retention of very narrow *Đ* (in THF at 30 °C, calibrated using polystyrene standards).

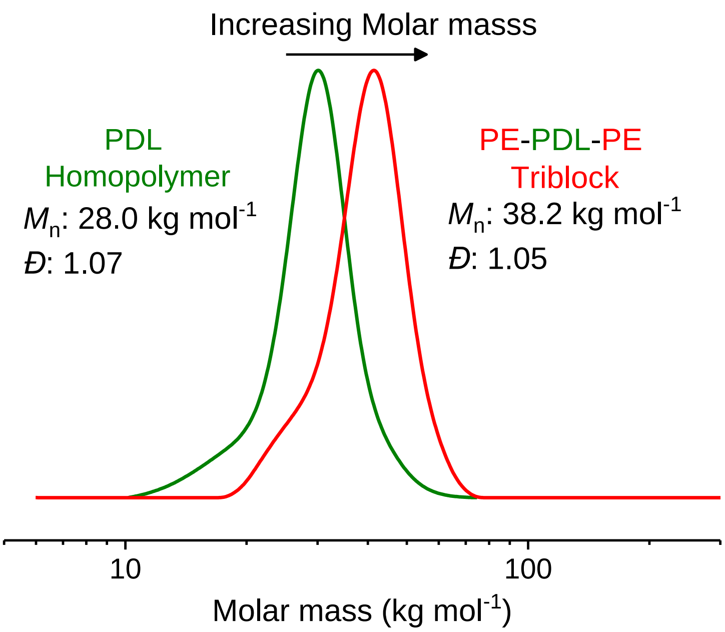

Figure S24. Stacked SEC traces of P6 illustrating an increase molar mass with retention of very narrow *Đ* (in THF at 30 °C, calibrated using polystyrene standards).

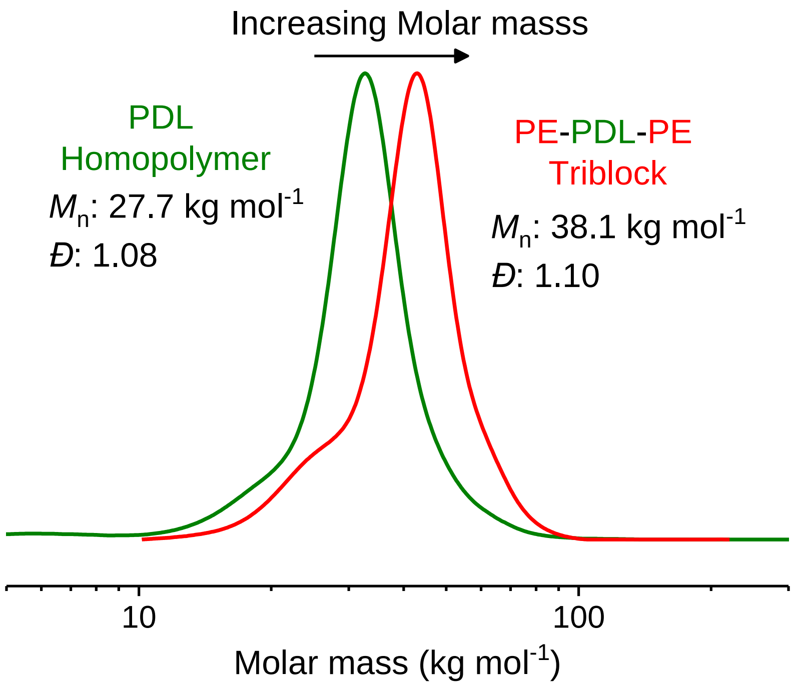

Figure S25. Stacked SEC traces of P7 illustrating an increase molar mass with retention of very narrow *Đ* (in THF at 30 °C, calibrated using polystyrene standards).

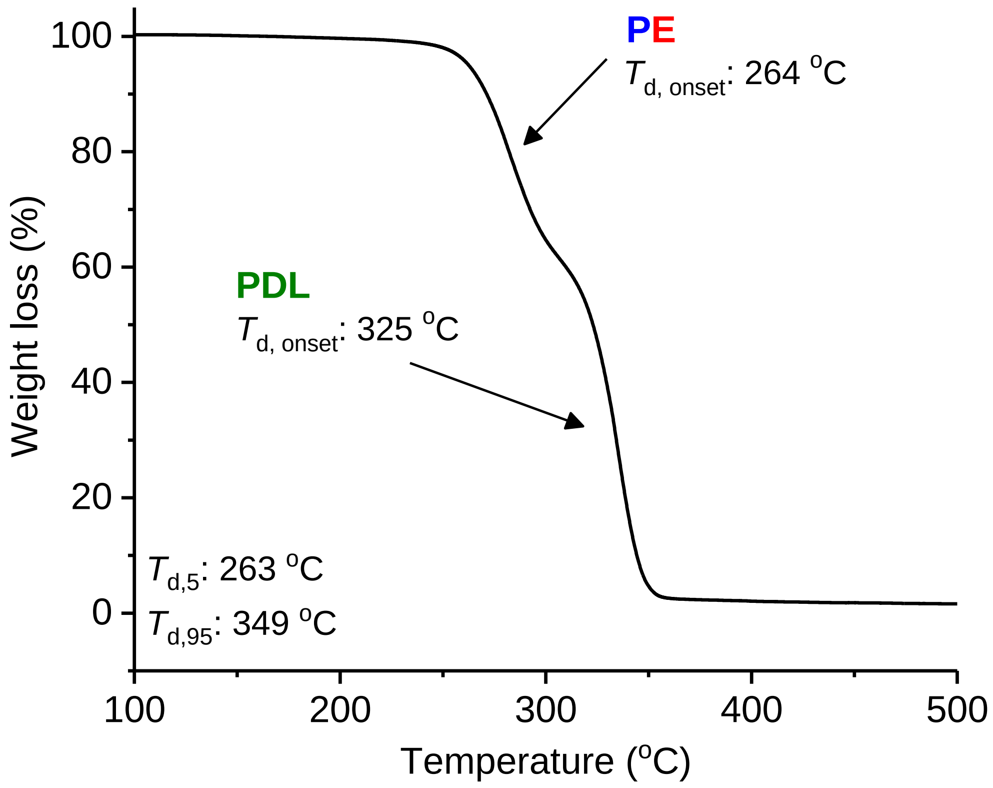

Figure S26. TGA thermograph of P3.

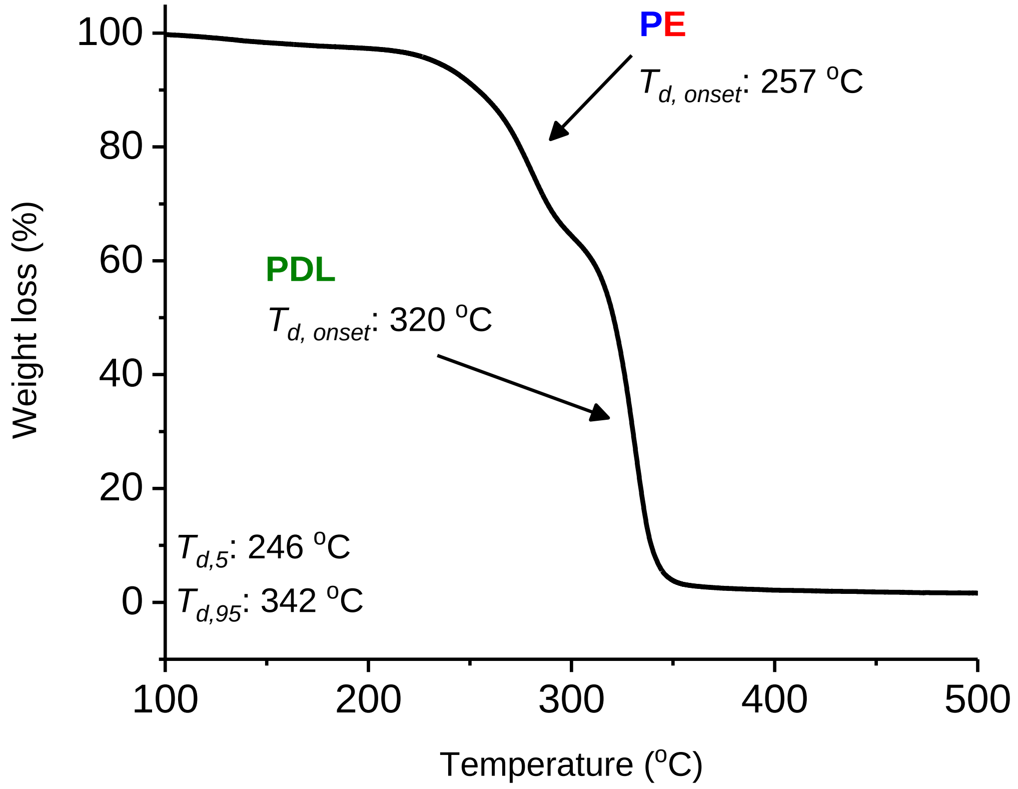

Figure S27. TGA thermograph of P5.

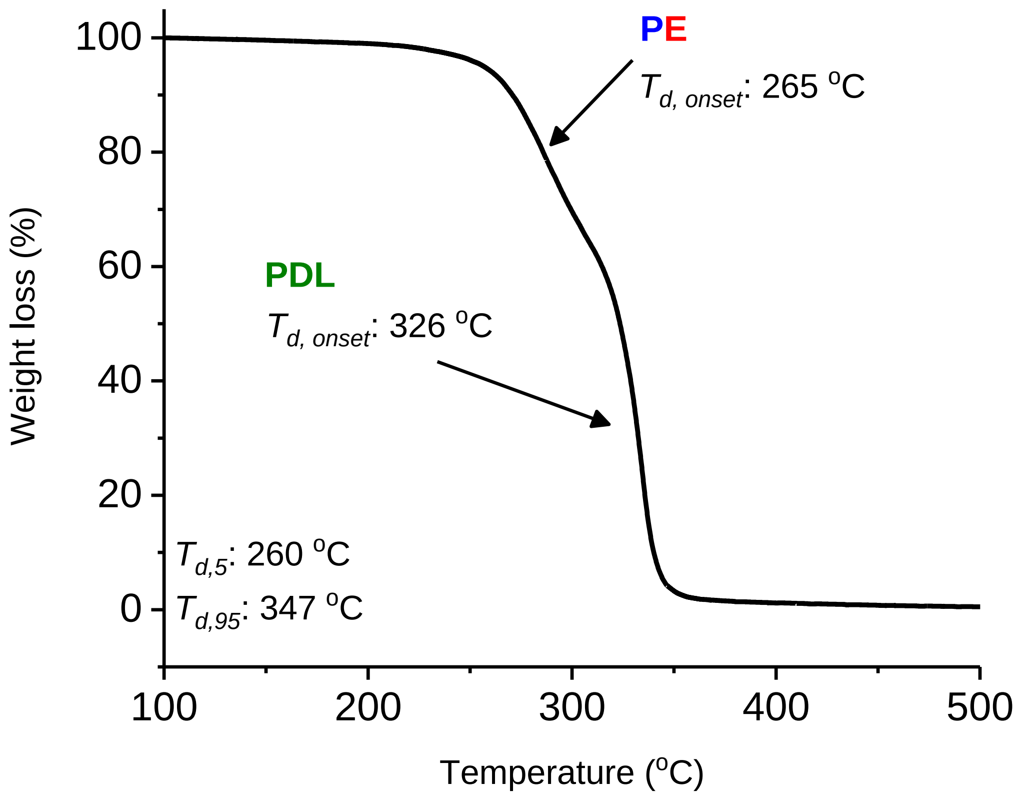

Figure S28. TGA thermograph of P6.

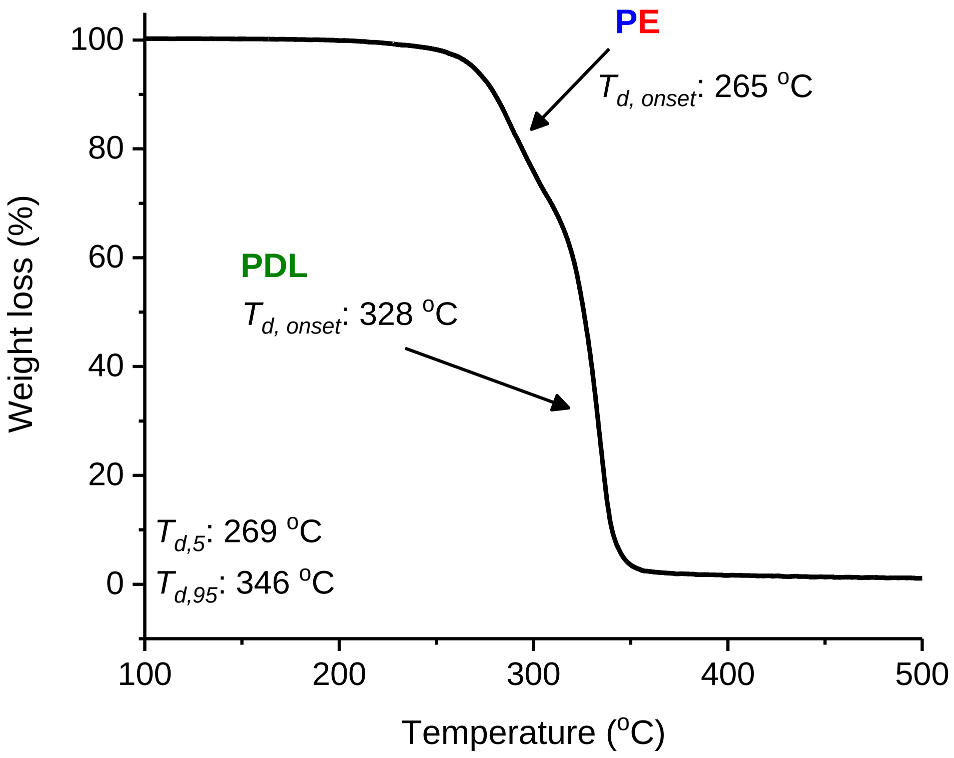

Figure S29. TGA thermograph of P7.

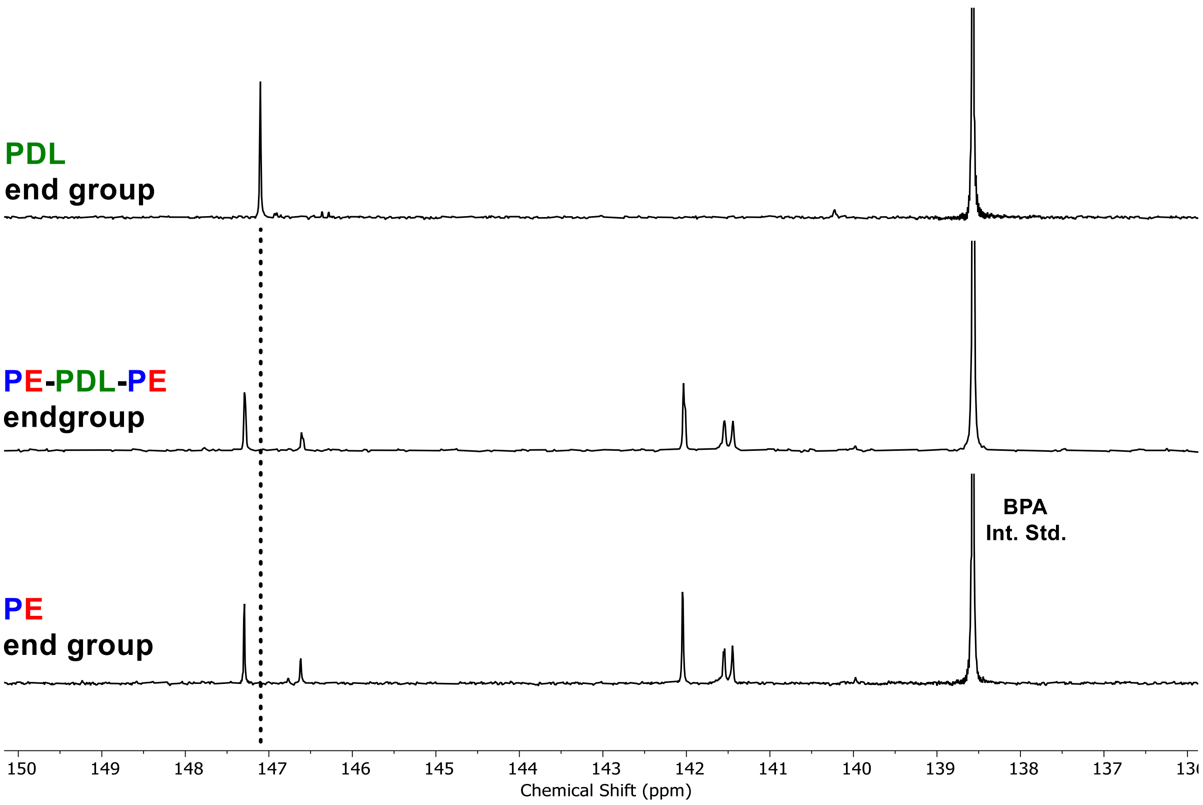

Figure S30. Selected region of $^{31}P\{^{1}H\}$ NMR spectra of P5 hydroxyl polymers showing a complete change in end group resonance (in $CDCl_3$).

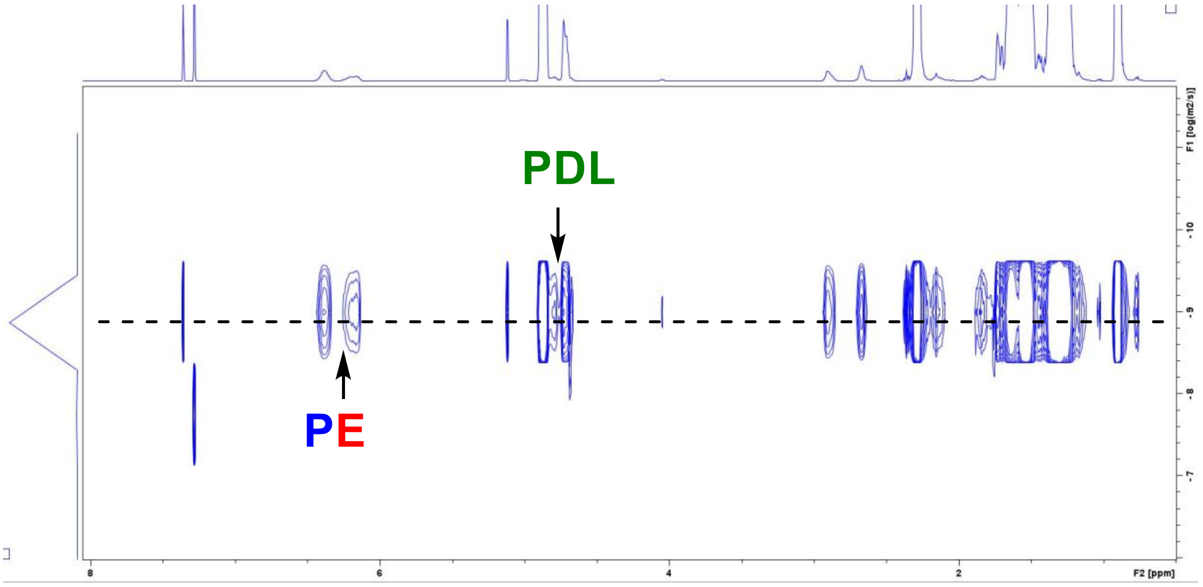

Figure S31. ^{1}H DOSY NMR spectrum showing a single diffusion coefficient for P5 (in $CDCl_3$).

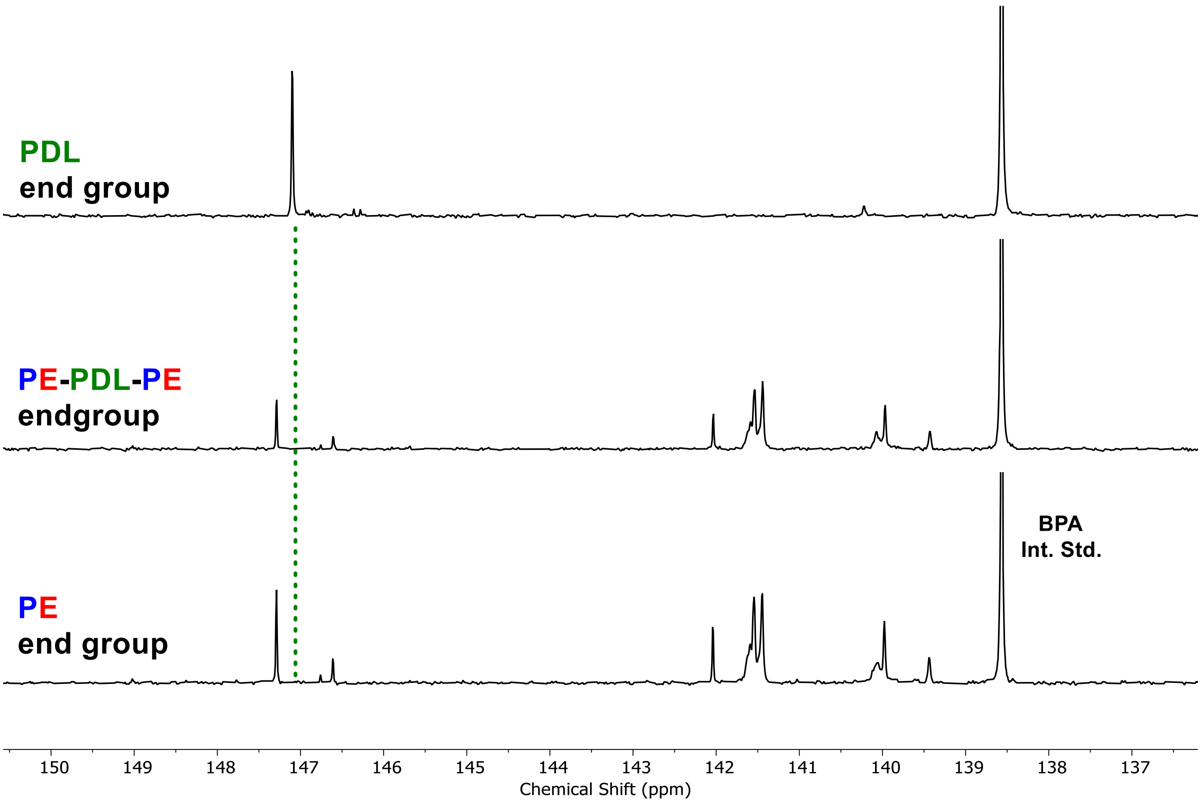

Figure S32. Selected region of $^{31}P\{^{1}H\}$ NMR spectra of P6 hydroxyl polymers showing a complete change in end group resonance (in $CDCl_3$).

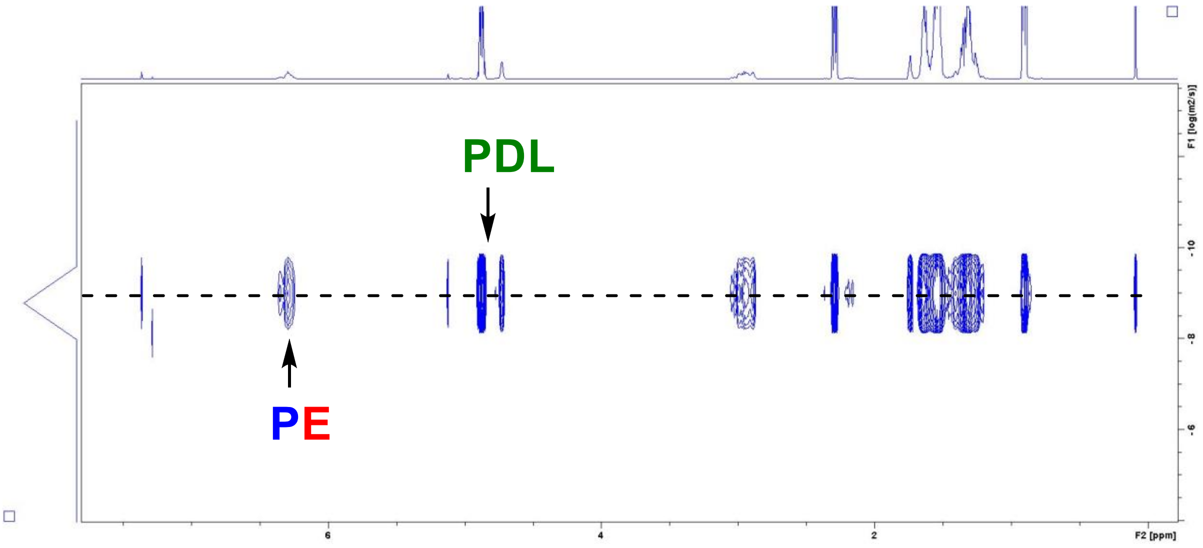

Figure S33. ^{1}H DOSY NMR spectrum showing a single diffusion coefficient for P6 (in $CDCl_3$).

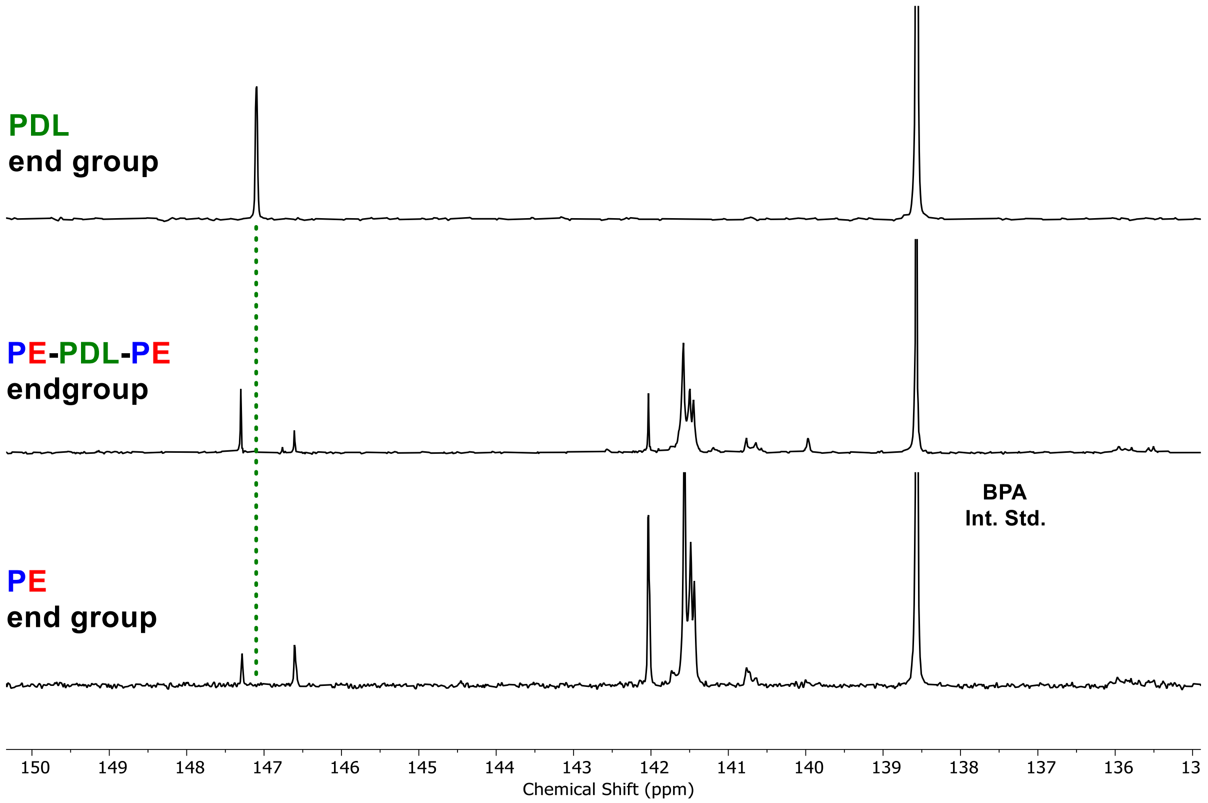

Figure S34. Selected region of $^{31}P\{^1H\}$ NMR spectra of P7 hydroxyl polymers showing a complete change in end group resonance (in $CDCl_3$).

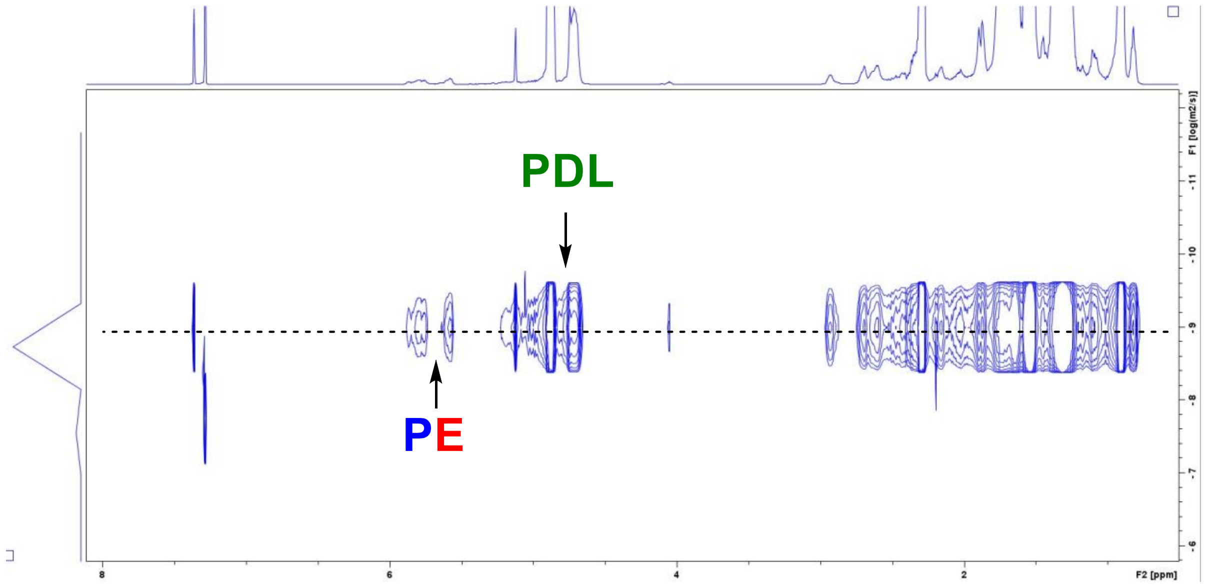

Figure S35. ^1H DOSY NMR spectrum showing a single diffusion coefficient for P7 (in $CDCl_3$).

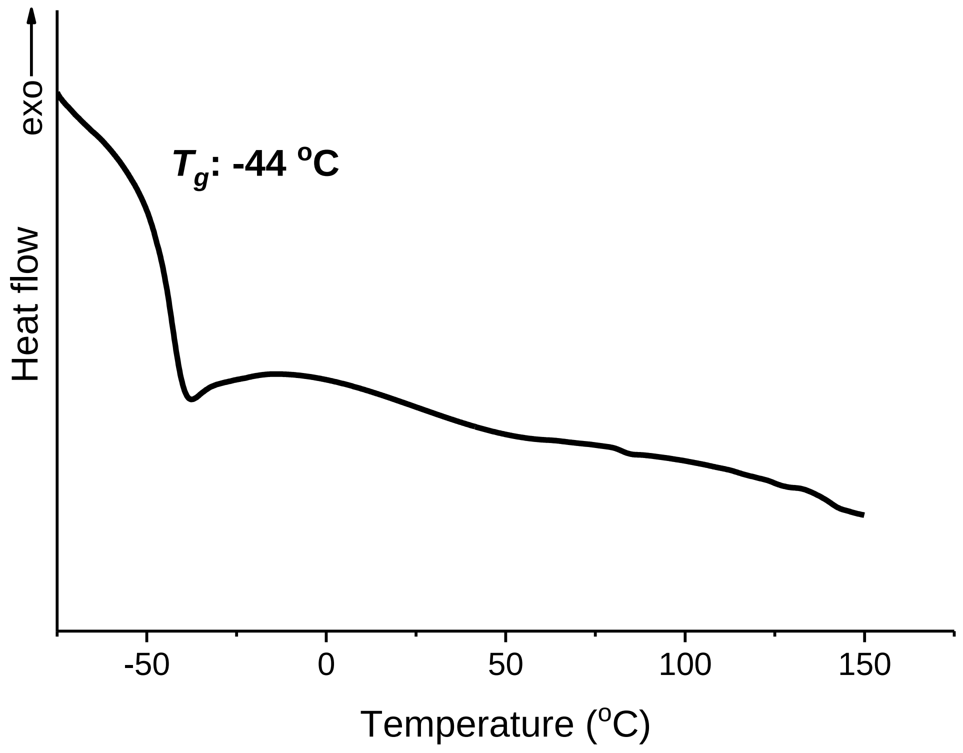

Figure S36. DSC thermography for P1.

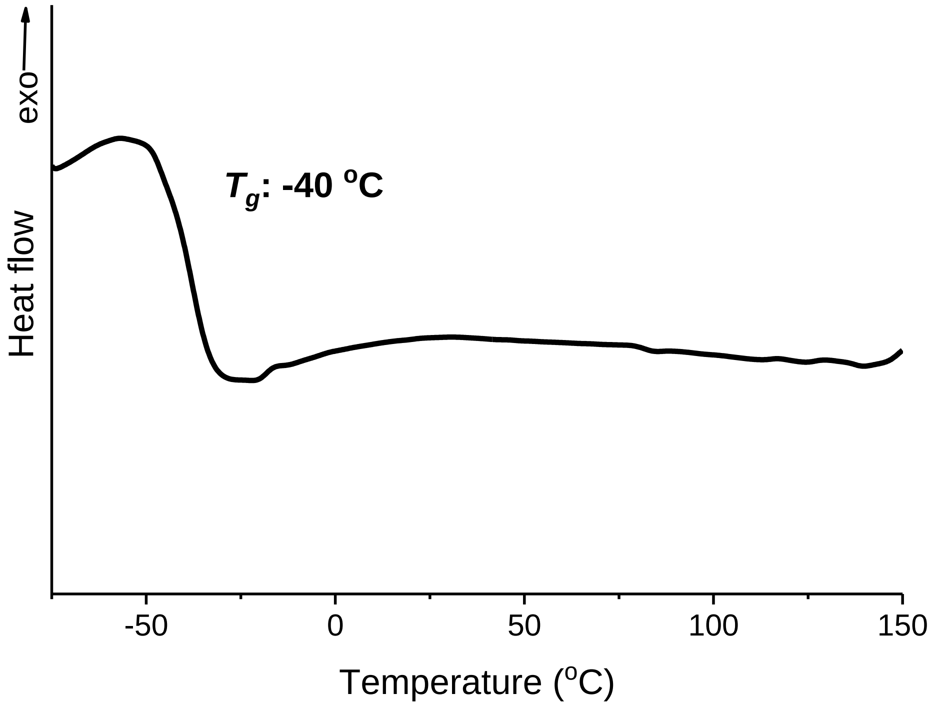

Figure S37. DSC thermography for P2

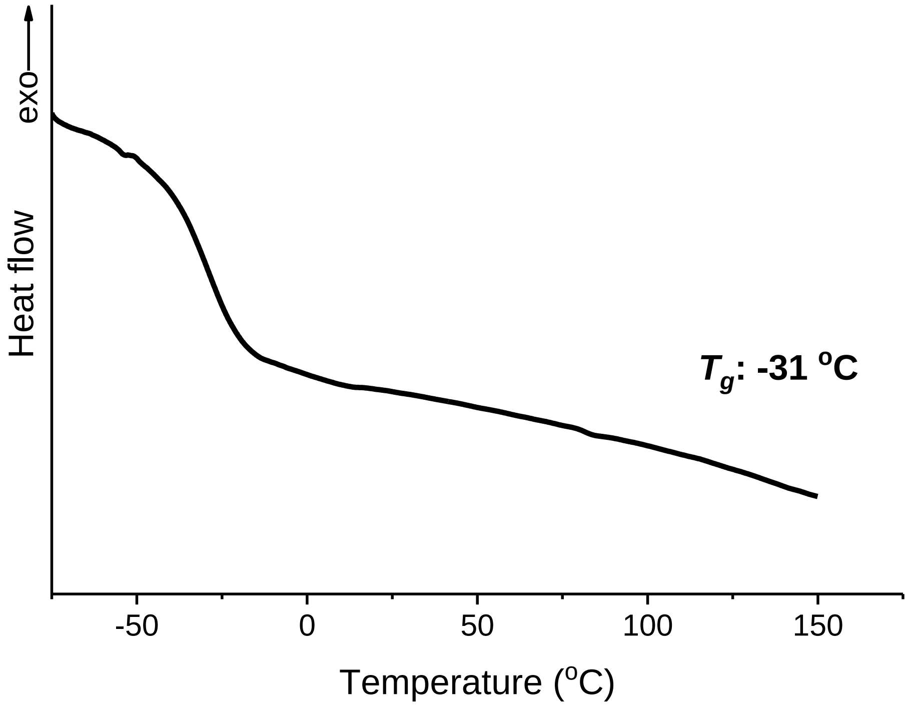

Figure S38. DSC thermography for P3

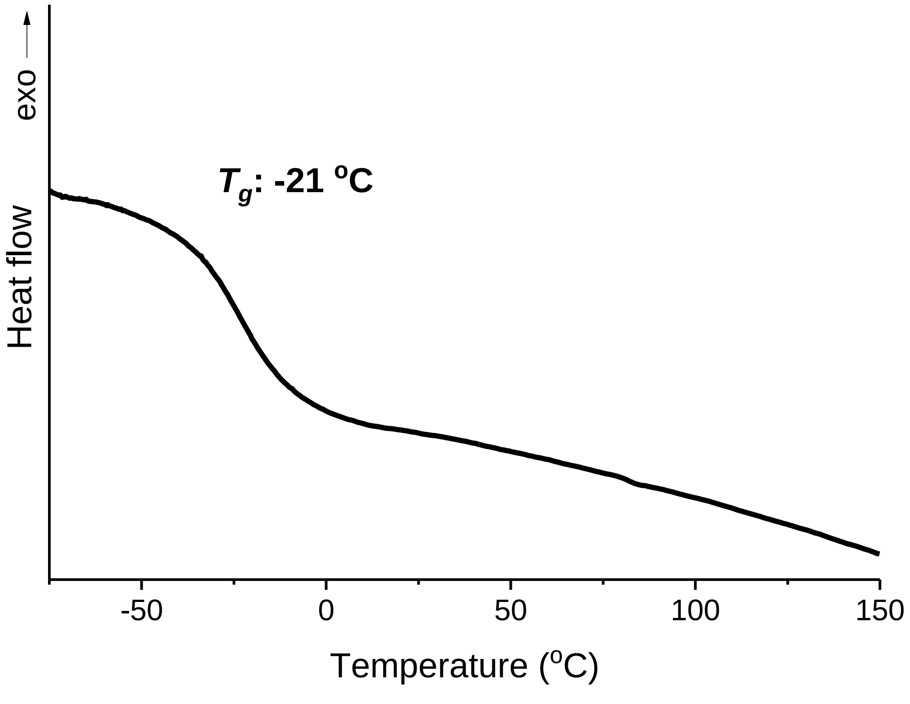

Figure S39. DSC thermography for P4

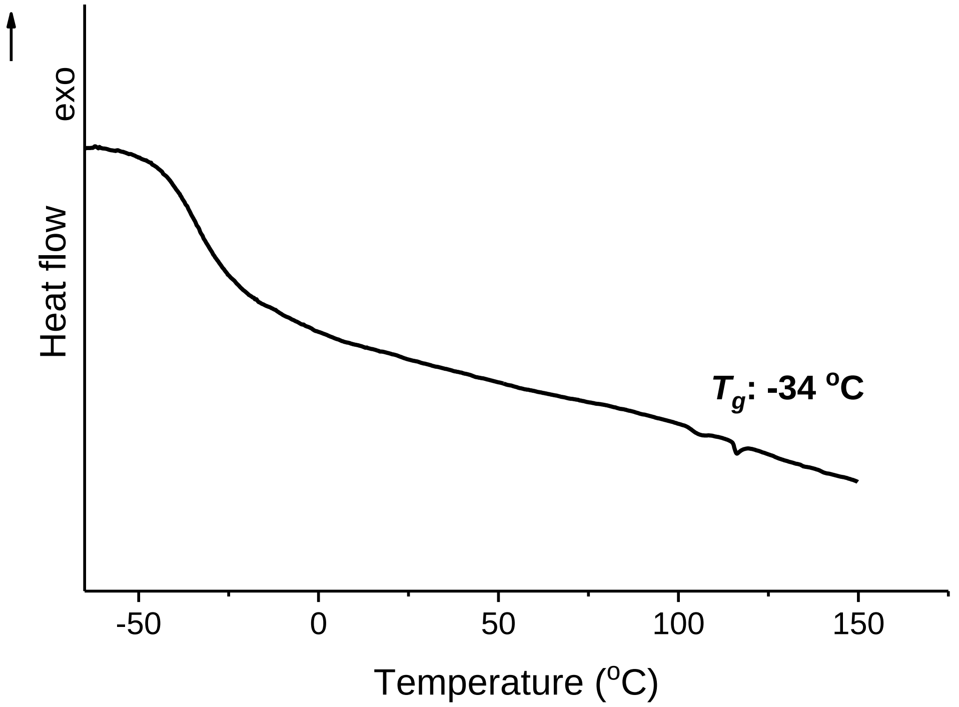

Figure S40. DSC thermography for P5

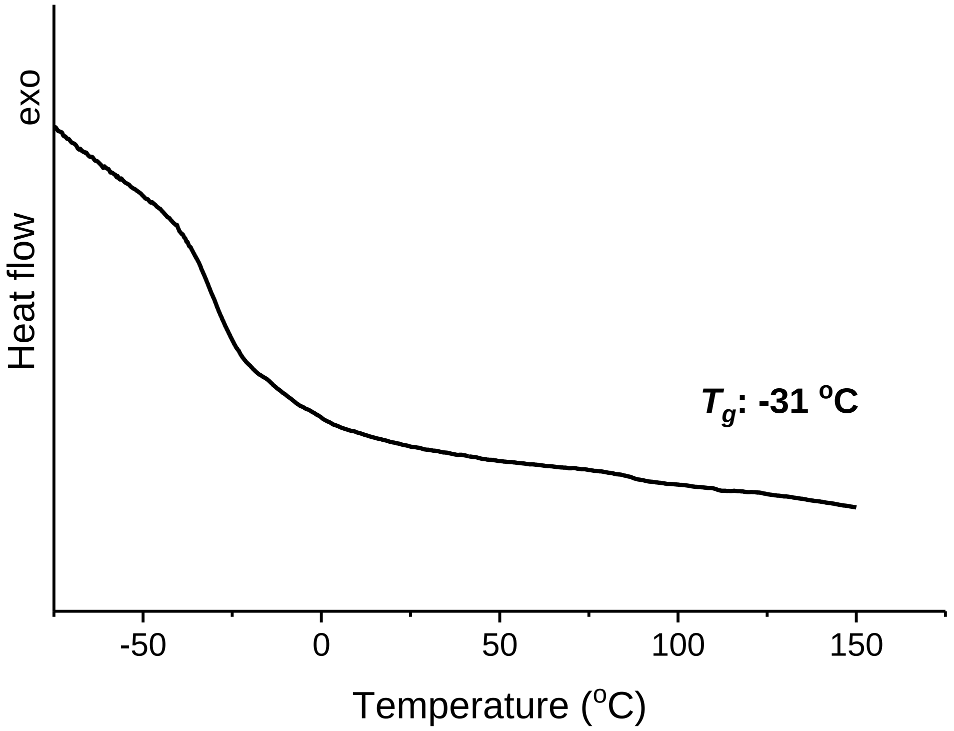

Figure S41. DSC thermography for P6

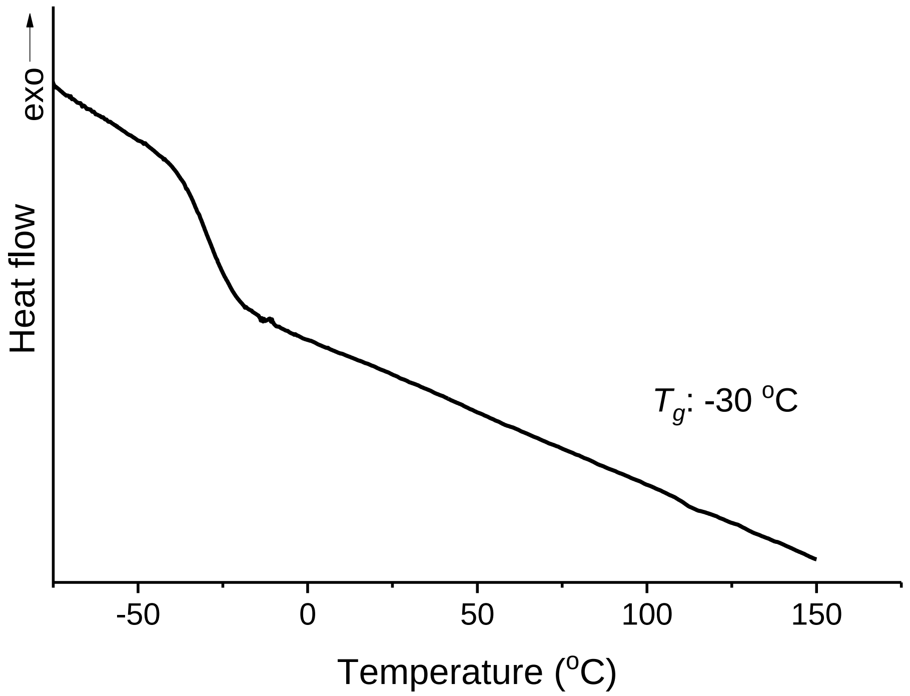

Figure S42. DSC thermography for P7

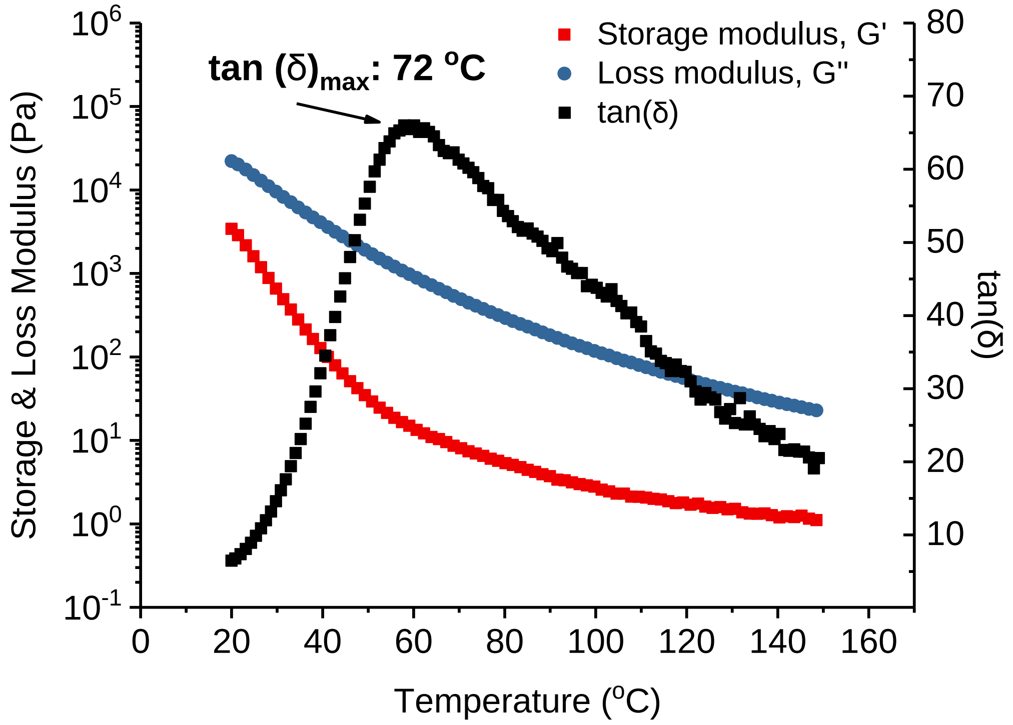

Figure S43. Storage & loss modulus and tan(δ) vs. temperature plot for P1

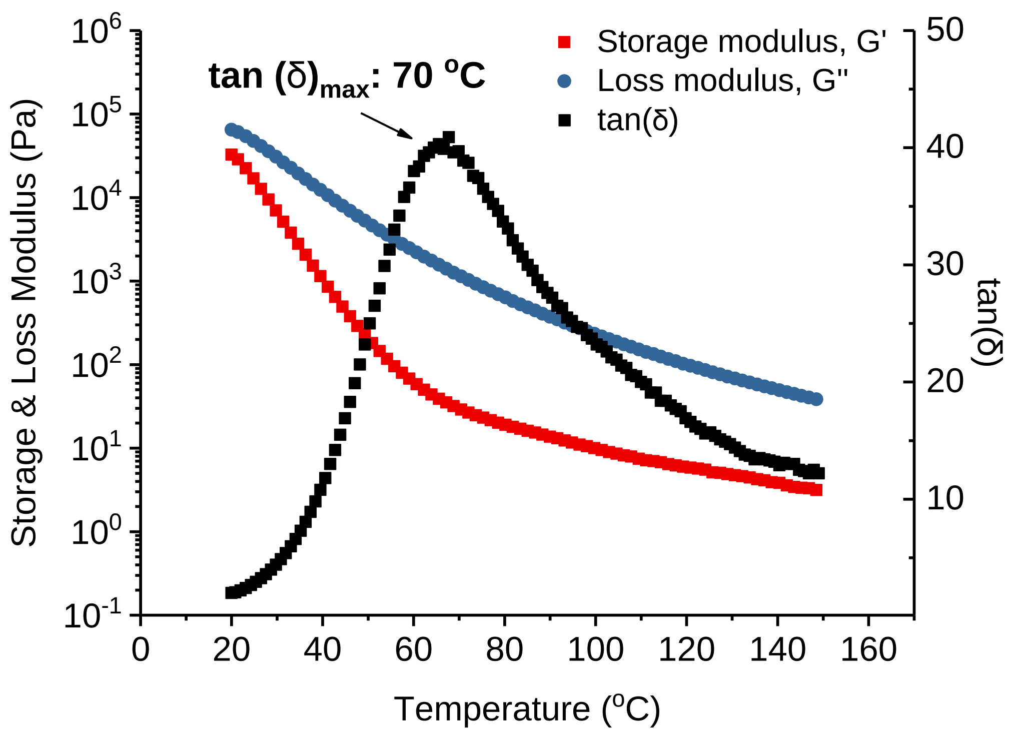

Figure S44. Storage & loss modulus and tan(δ) vs. temperature plot for P2

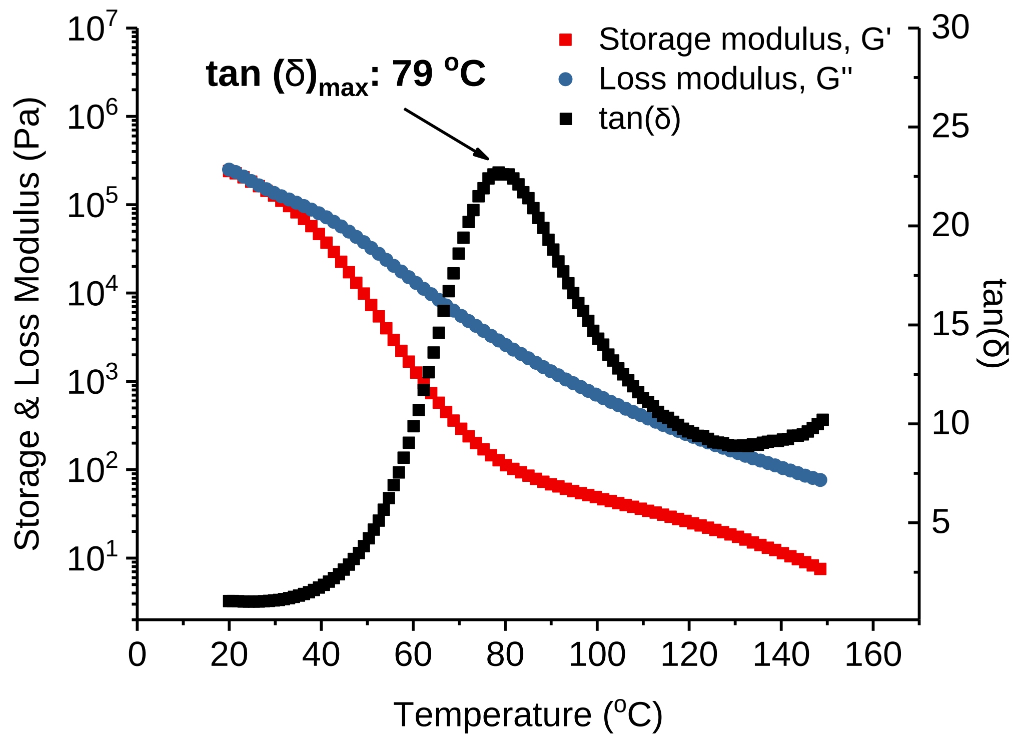

Figure S45. Storage & loss modulus and tan(δ) vs. temperature plot for P3

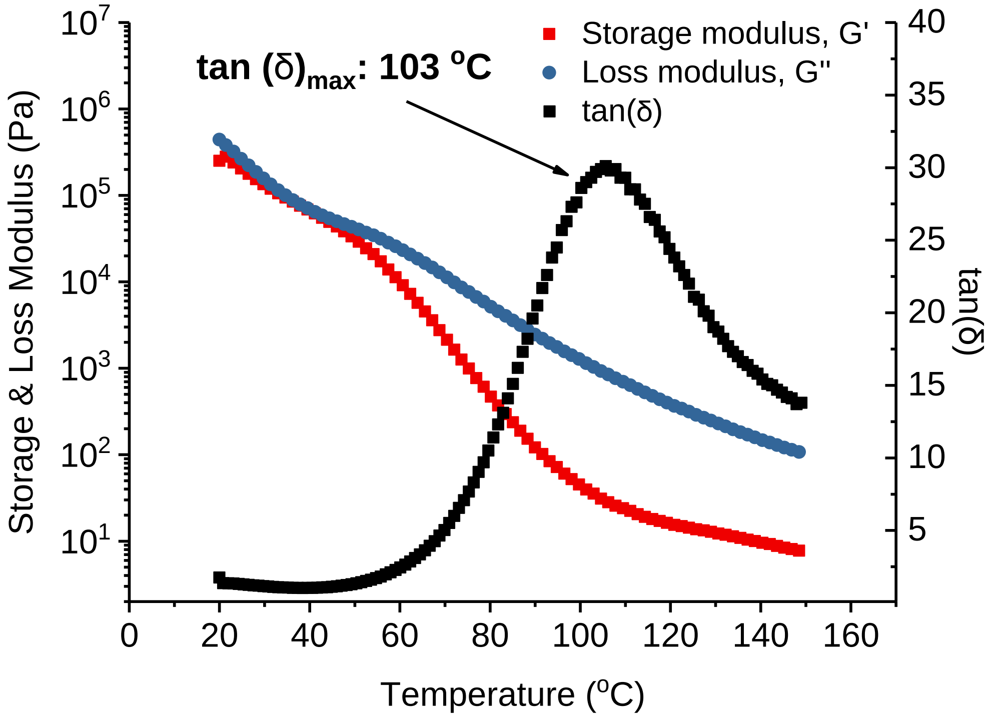

Figure S46. Storage & loss modulus and tan(δ) vs. temperature plot for P4

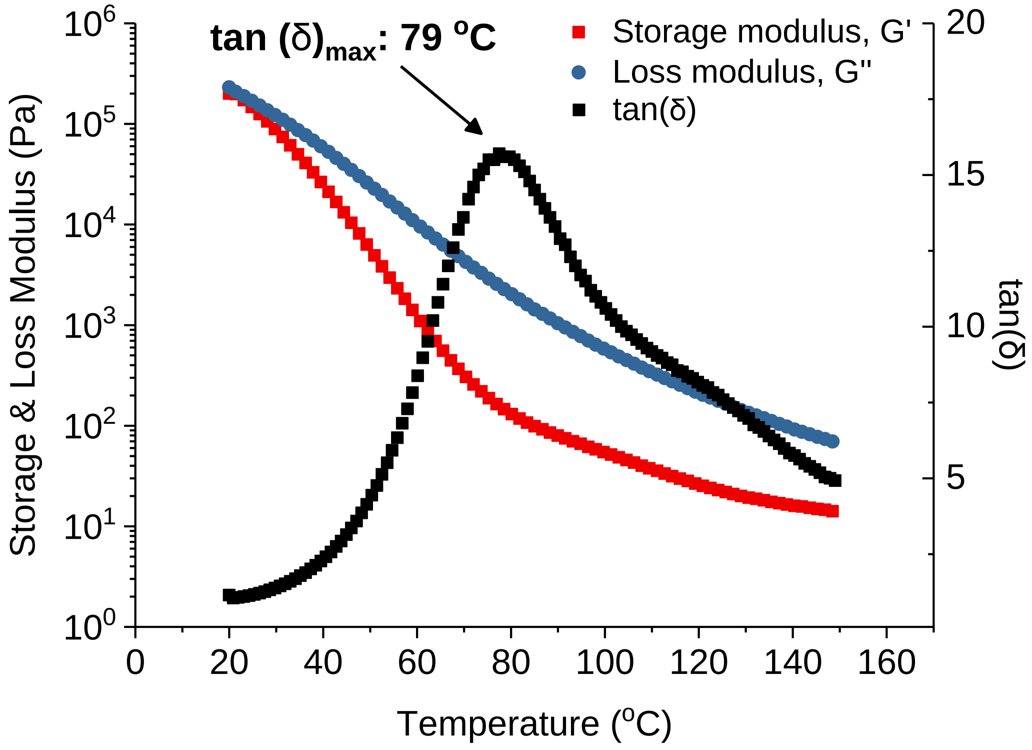

Figure S47. Storage & loss modulus and tan(δ) vs. temperature plot for P5.

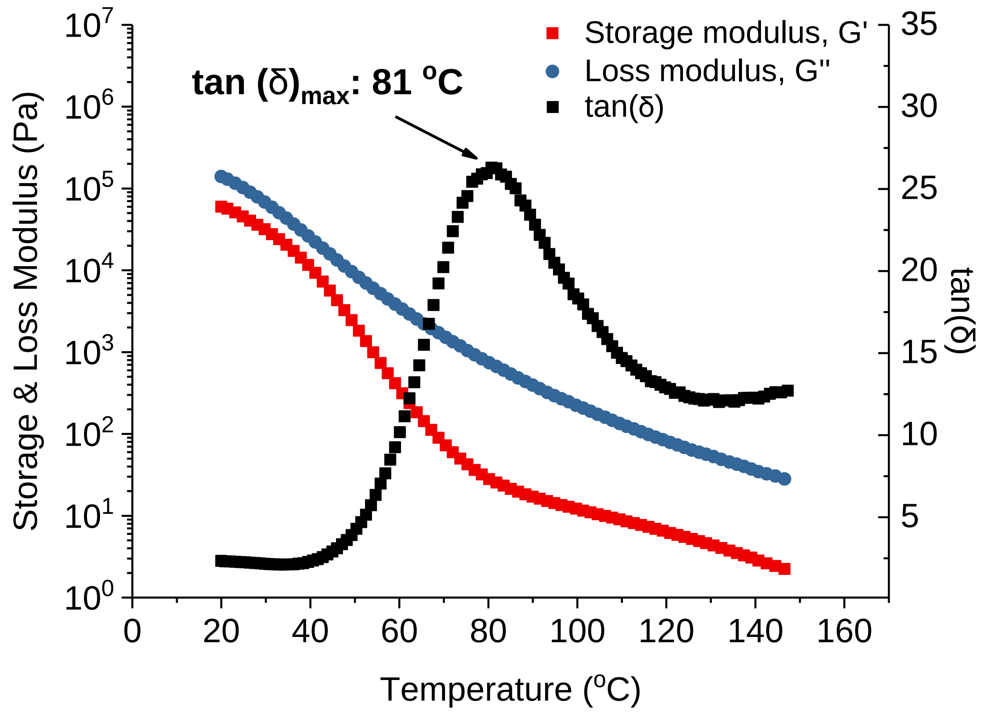

Figure S48. Storage & loss modulus and tan(δ) vs. temperature plot for P6

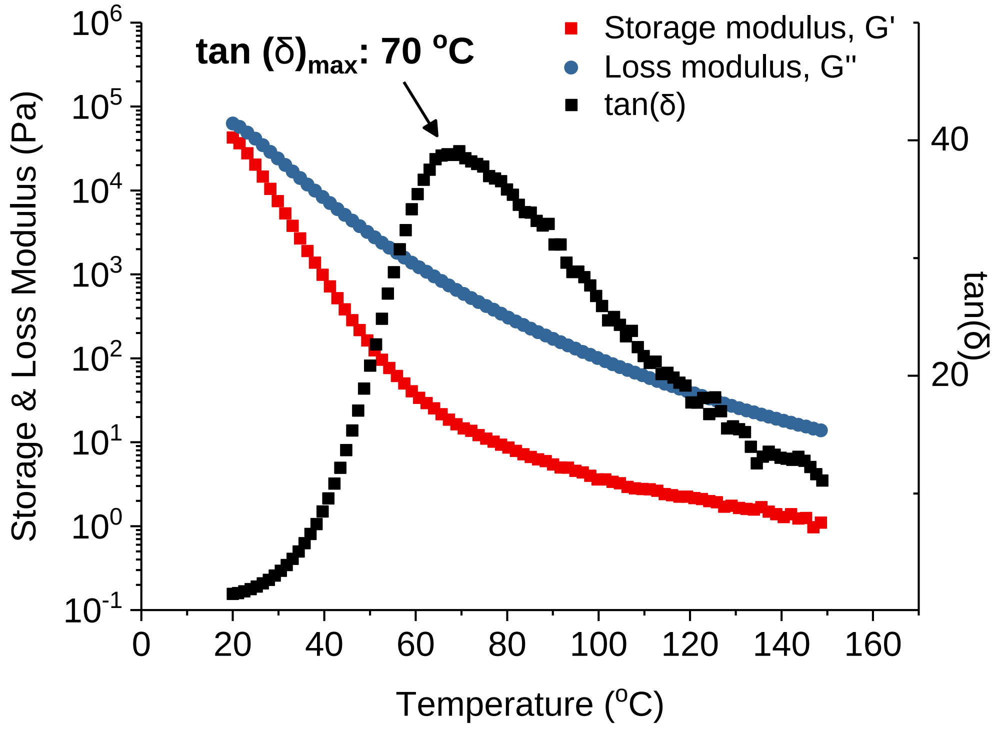

Figure S49. Storage & loss modulus and tan(δ) vs. temperature plot for P7

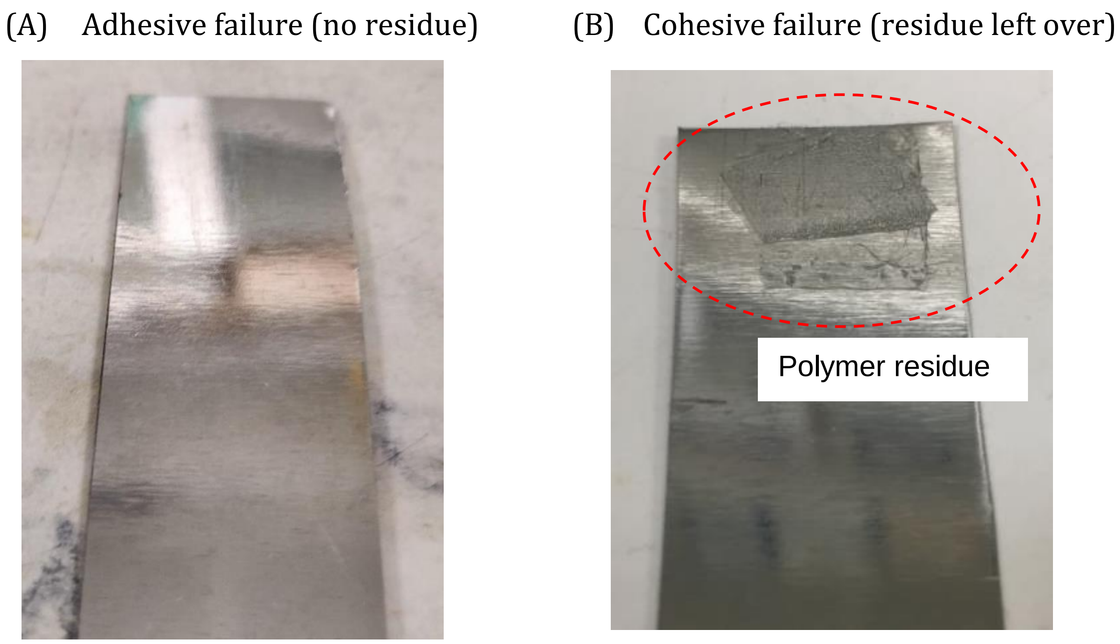

Figure S50. Stainless steel substrate photograph post-peel test show the difference between (A) an adhesive mode of failure with no polymer residue left over and (B) a cohesive mode of failure with visible polymer residue on the substrate.

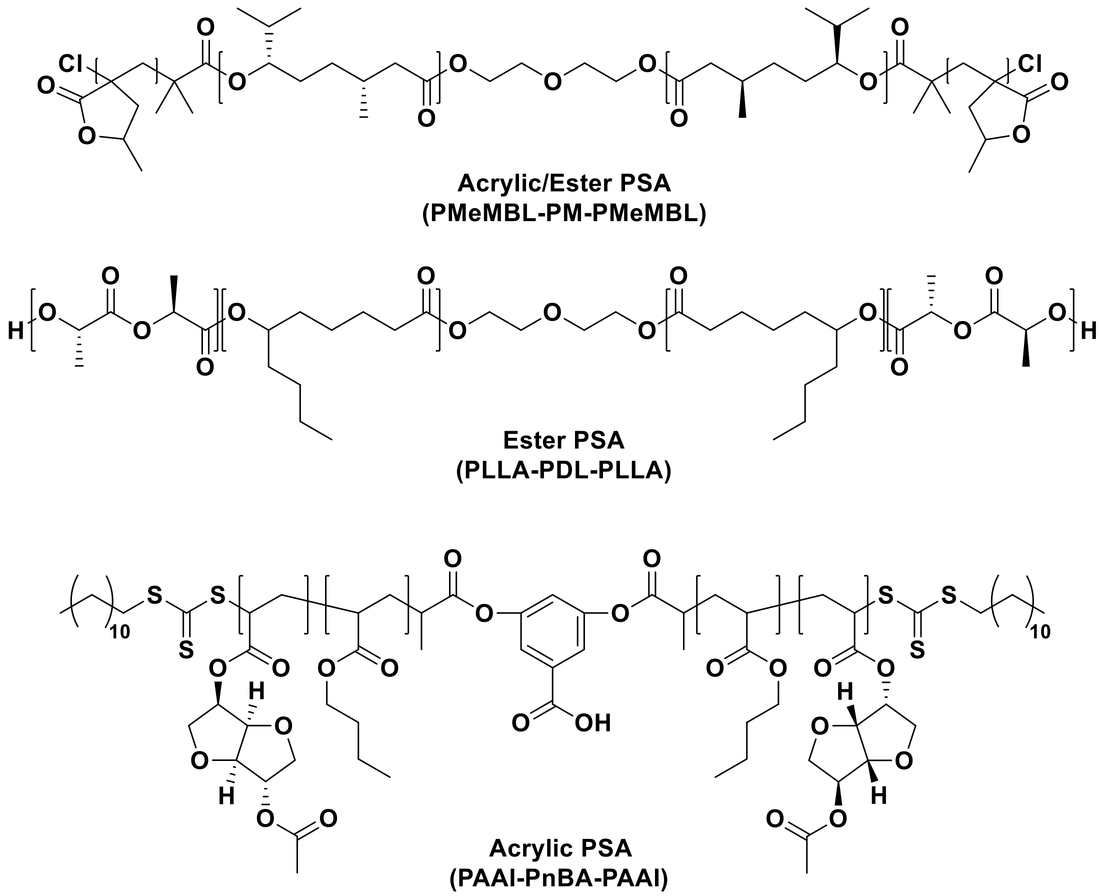

Figure S51. Literature examples of triblock polymer-based PSA

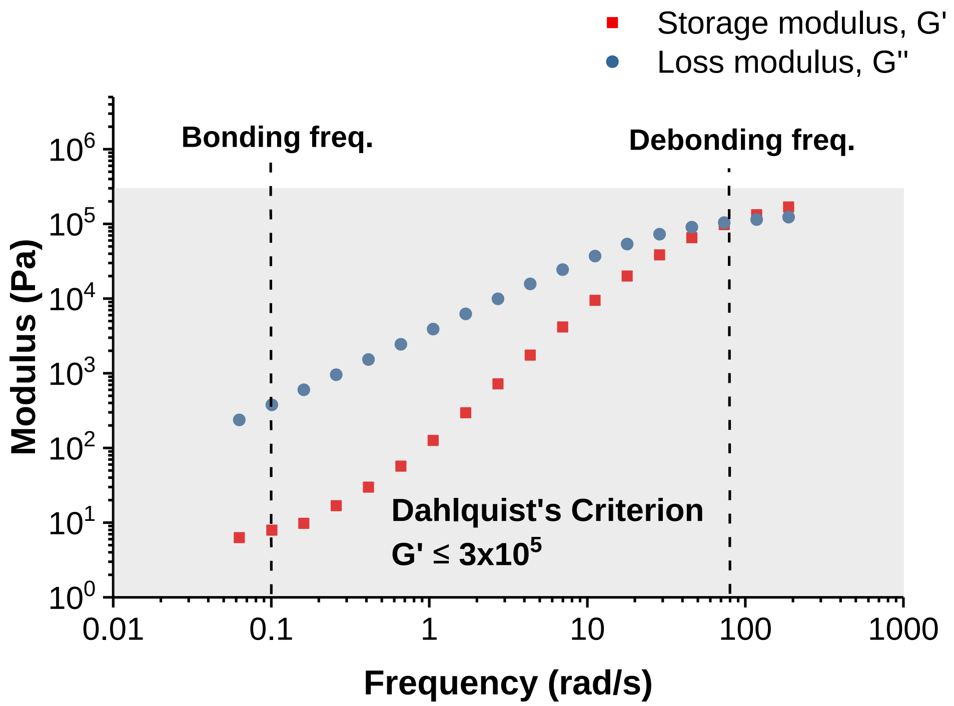

Figure S52. Storage & loss modulus vs. frequency plot for P1.

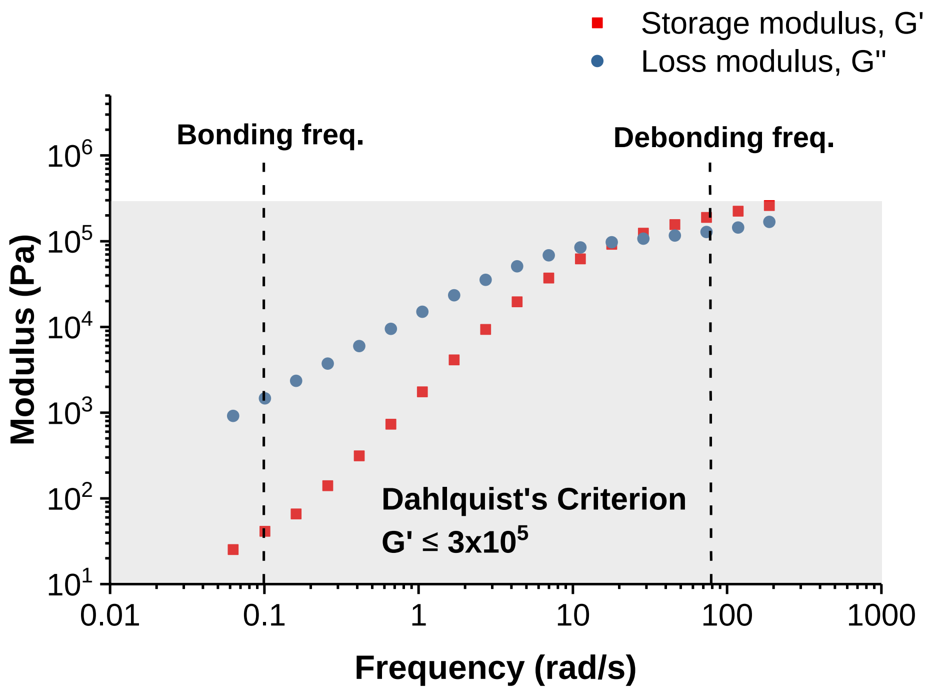

Figure S53. Storage & loss modulus vs. frequency plot for P2

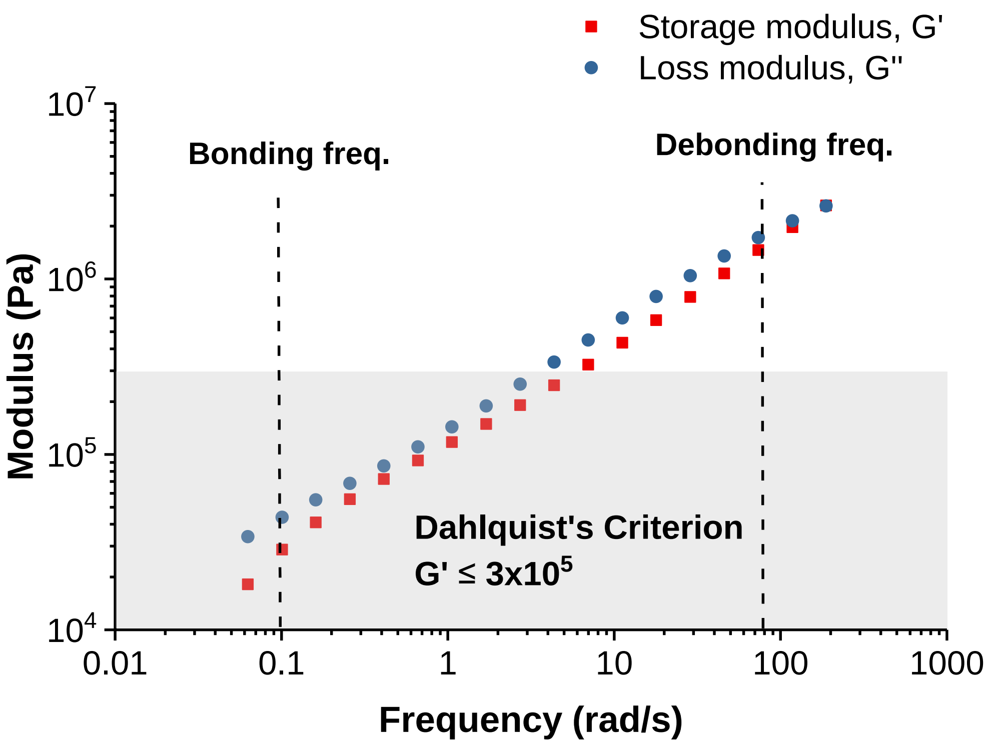

Figure S54. Storage & loss modulus vs. frequency plot for P4

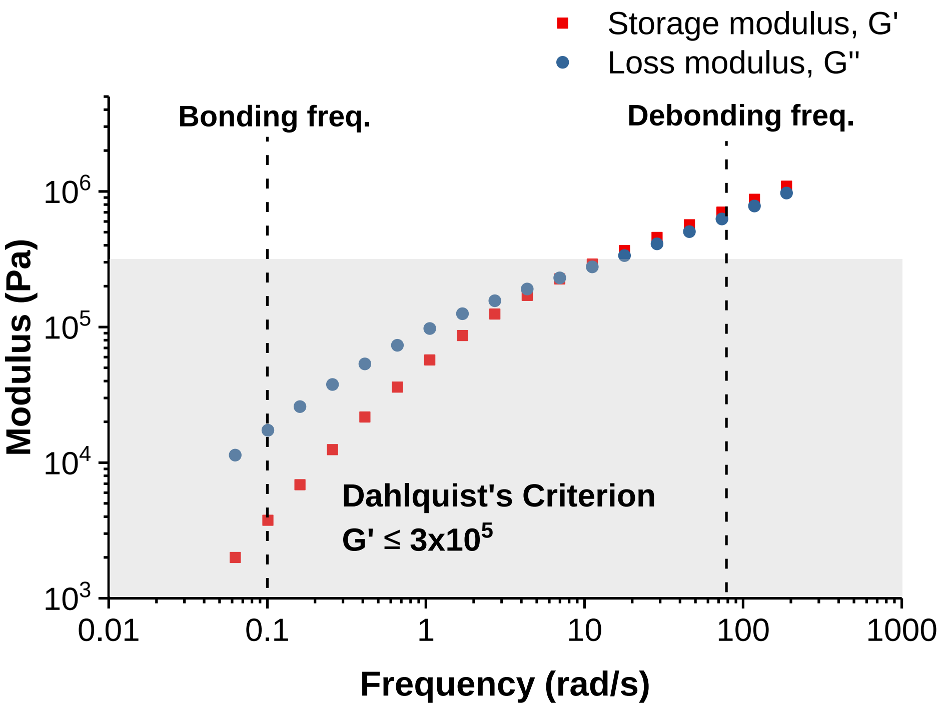

Figure S55. Storage & loss modulus vs. frequency plot for P5

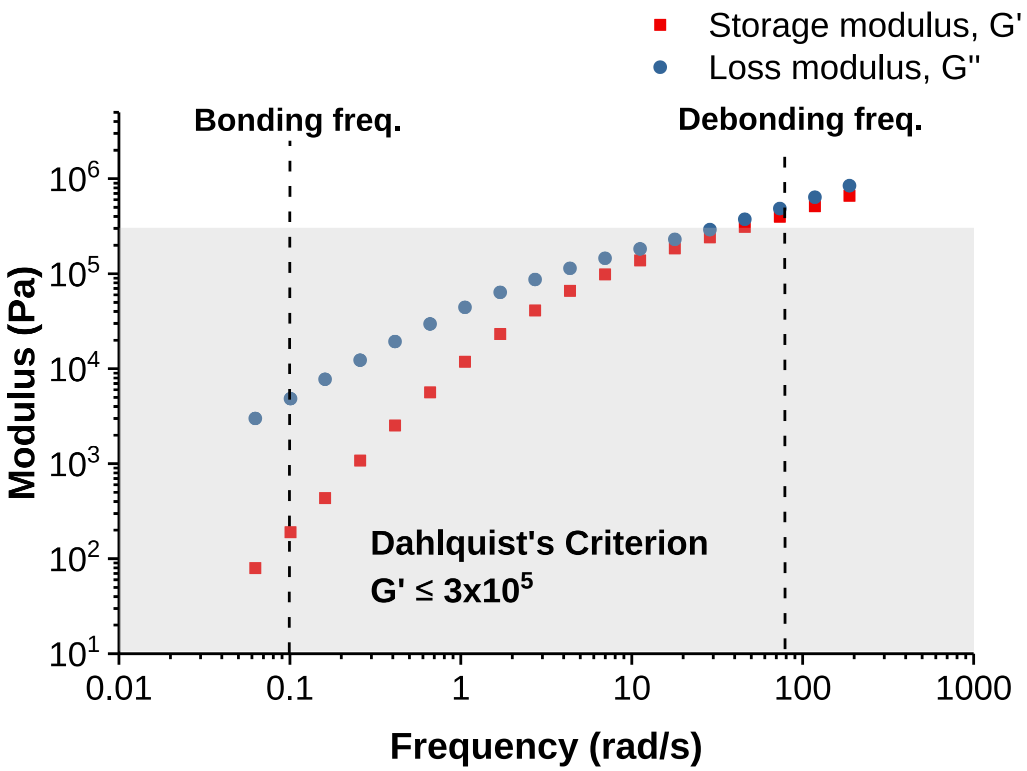

Figure S56. Storage & loss modulus vs. frequency plot for P6

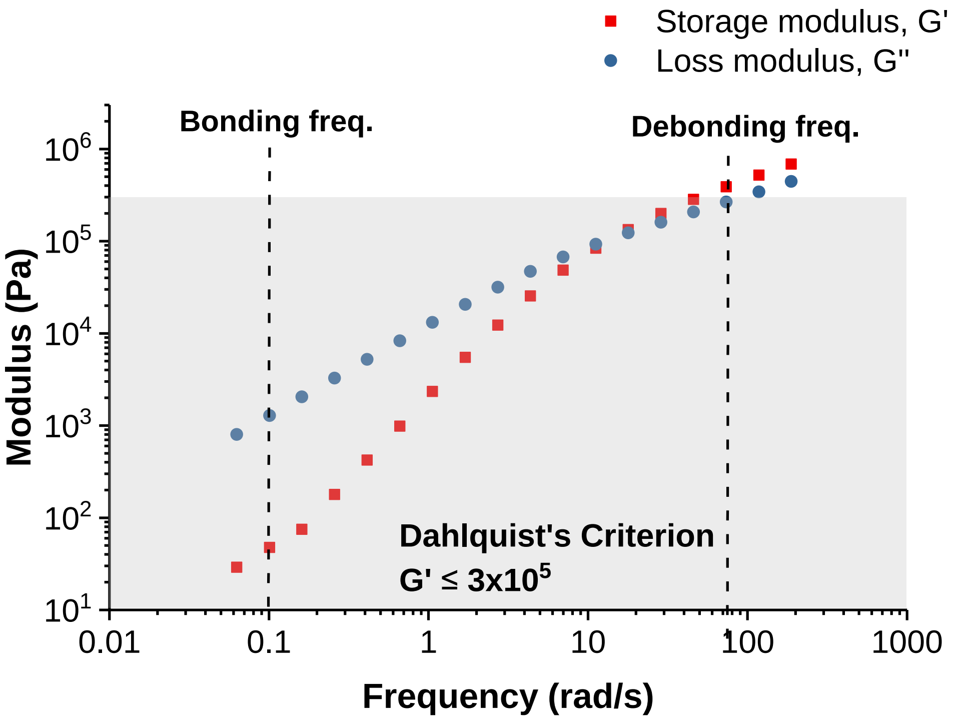

Figure S57. Storage & loss modulus vs. frequency plot for P7.

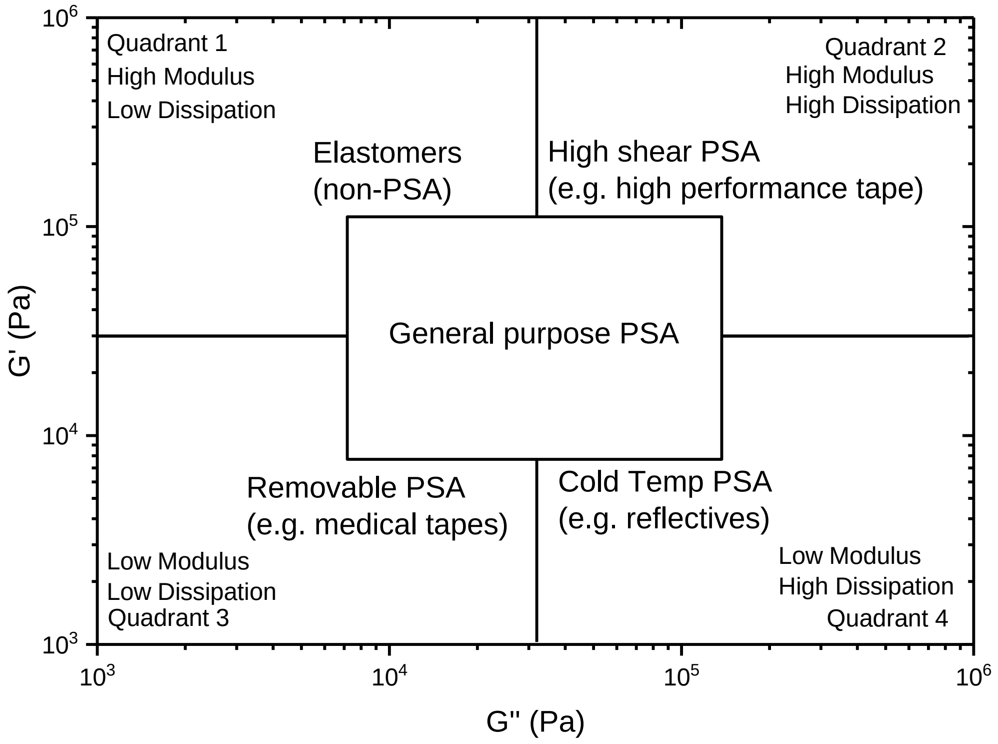

Figure S58. Viscoelastic window for qualitative analysis of PSA's potential usage (as developed by Chang *et al.*)[13]

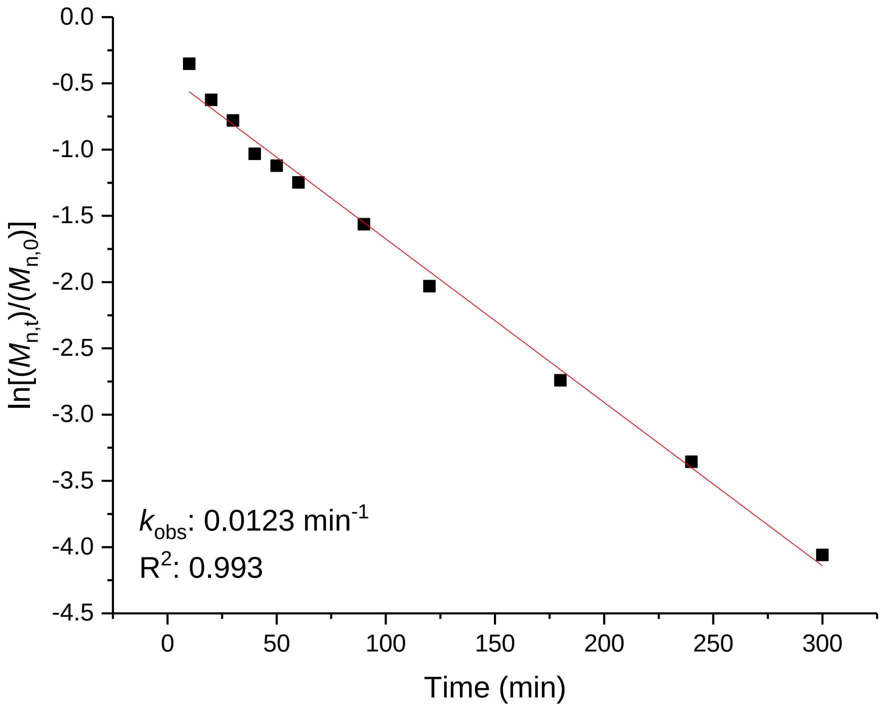

Figure S59.Plot of $Ln(Mn_0/M_n)$ vs. Time for P3 under acid-catalyzed degradation (THF, *p*TSA, 60 °C).

Based on first-order kinetic model: Ln(Mn, t) = Ln(Mn,0)-kdt, where Mn is the number averaged molar mass determined by SEC. kd = degradation rate constant and t = degradation time.

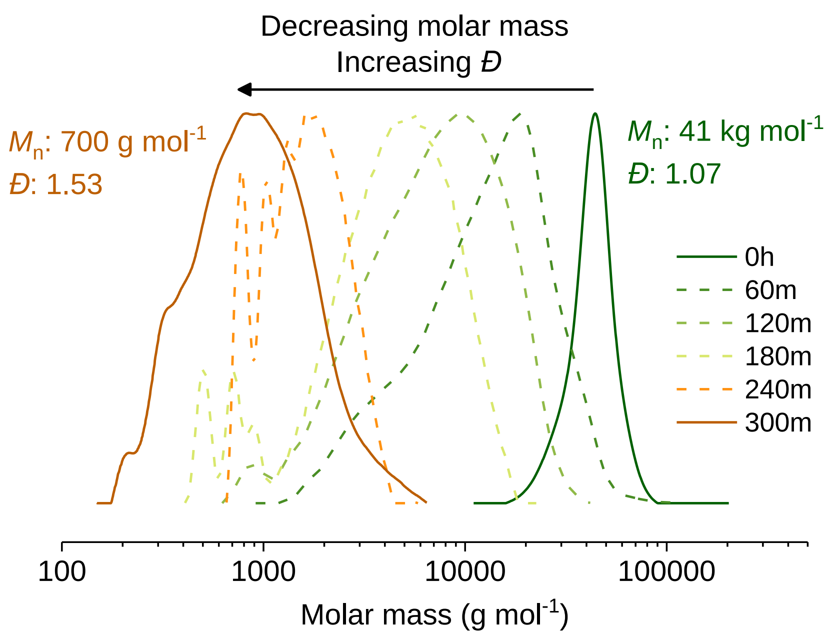

Figure S60. Stacked SEC traces for the acid degradation test on P3 (1M in THF, 60 °C).

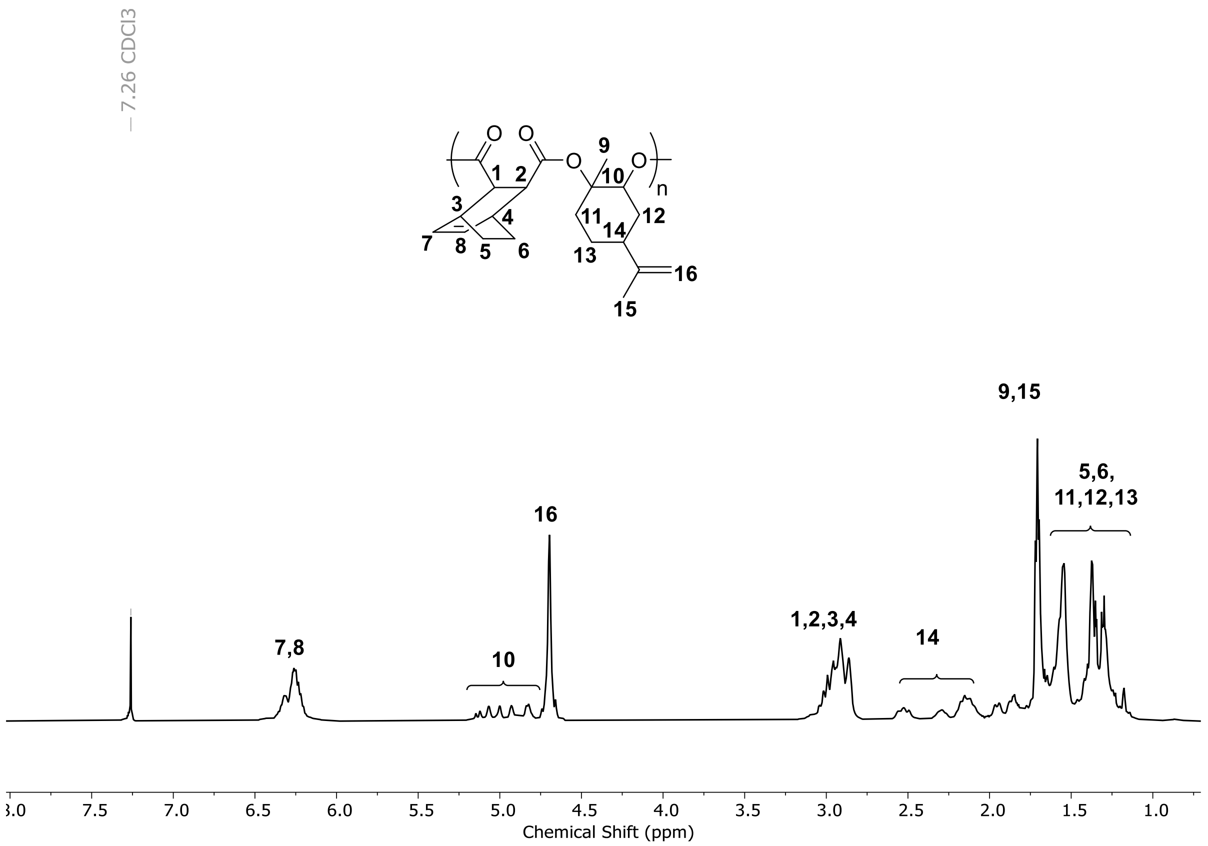

Figure S61. ^{1}H NMR of the polyester from the ROCOP of LO/TCA 2 in $CDCl_3$.

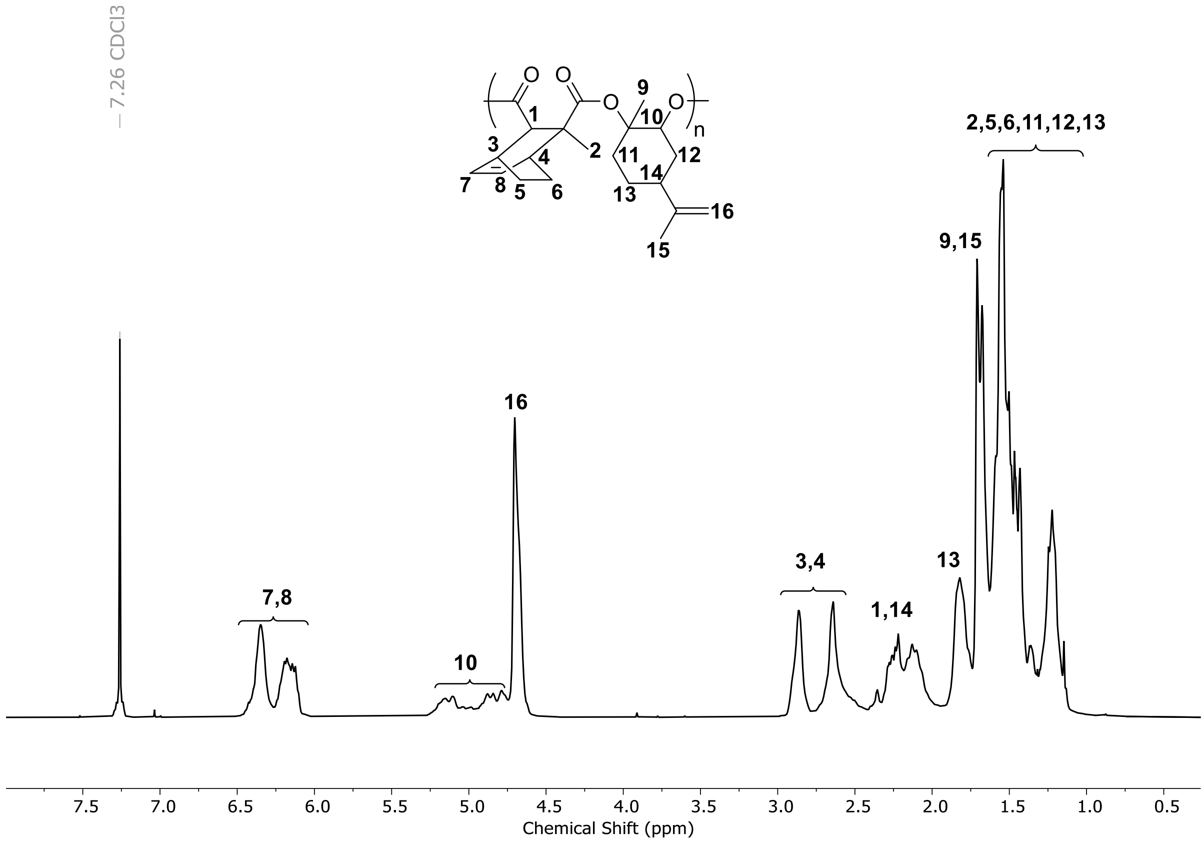

Figure S62. ^{1}H NMR of the polyester from the ROCOP of LO/TCA 3 in $CDCl_3$.

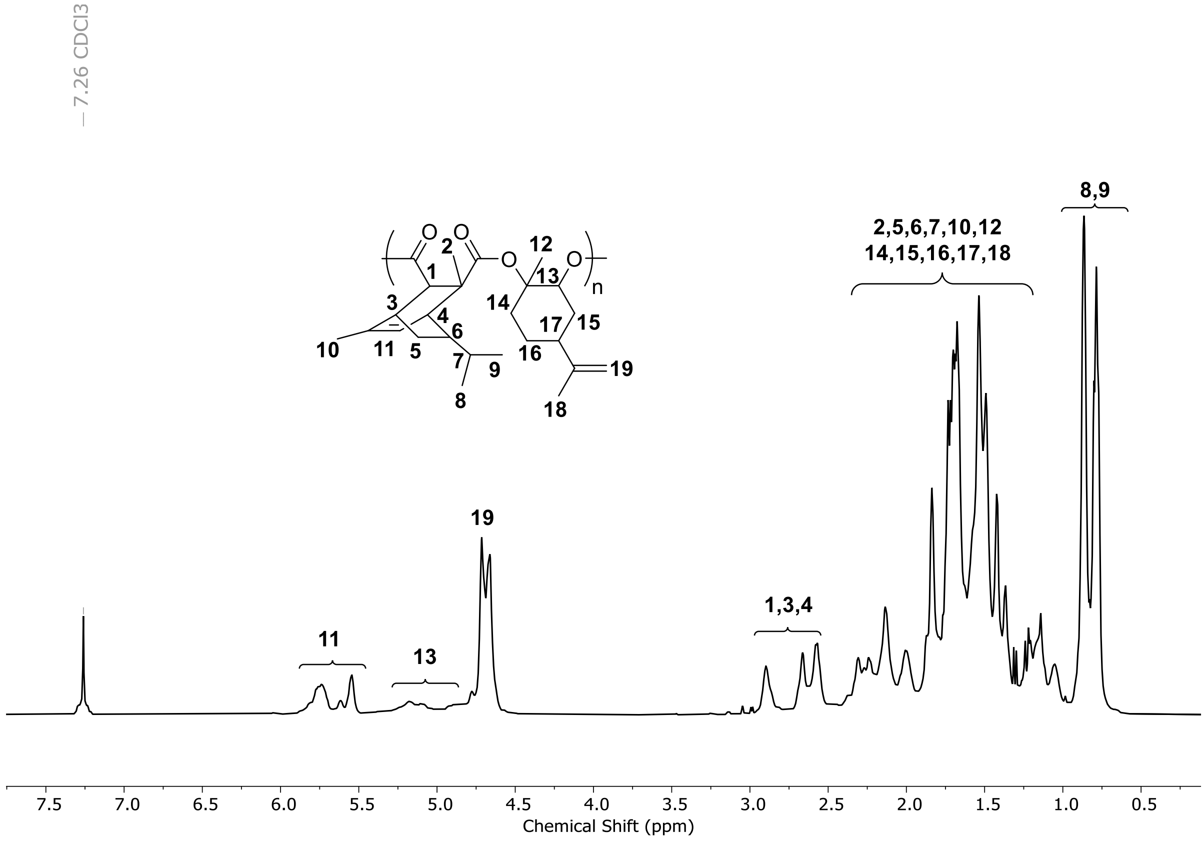

Figure S63. ^{1}H NMR of the polyester from the ROCOP of LO/TCA 4 in $CDCl_3$.

Table S4. Theoretical T_g for fully miscible blocks

Polymer	wt%$_{hard}$[a]	$T_{g, hard}$[b]	wt%$_{soft}$[c]	$T_{g, soft}$[d]	$T_{g, miscible}$[e]
P1	21	93	79	-50	-30
P2	30	93	70	-50	-20
P3	41	93	59	-50	-7
P4	49	93	51	-50	1
P5	41	102	59	-50	-6
P6	41	82	59	-50	-10
P7	40	88	60	-50	-10

[a]wt% of hard block, [b]T_g for hard block, [c]wt% of soft block, [d]T_g for soft block, [e]T_g for the sample if the blocks were miscible (as calculated using the Fox equation: $1/T_{g,miscible} = wt\%_{hard}/T_{g,hard} + wt\%_{soft}/T_{g,soft}$)

Table S5. Adhesive performance comparison against selected examples of bio-based polymers in the literature

Polymer Sample	Nature of the polymer	Contains tackifier?	Polymer cross-linked?	Failure mode*	Peel Adhesion (N/cm)	Ref.
P1	Bio-derived, degradable triblock polyester	No	No	Cohesive	0.1	This work
P2	Bio-derived, degradable triblock polyester	No	No	Cohesive	2.4 ± 0.03	This work
P3	Bio-derived, degradable triblock polyester	No	No	Adhesive	10.8 ± 0.4	This work
P4	Bio-derived, degradable triblock polyester	No	No	Adhesive	4.0 ± 0.5	This work
P5	Bio-derived, degradable triblock polyester	No	No	Adhesive	13.1 ± 0.6	This work
P6	Bio-derived, degradable triblock polyester	No	No	Adhesive	10.5 ± 0.3	This work
P7	Bio-derived, degradable triblock polyester	No	No	Adhesive	8.1 ± 0.6	This work
Aliphatic polyester	Polyester from polycondensation of dimethyl 1,4-cyclohexane dicarboxylate and diethylene glycol	No	No	Cohesive	10.5 ± 0.4	[14]
Polyester based masking tape	Polyester from polycondensation of long chain dimer acids and diols	Yes (20 wt%)	Yes (4 wt%)	Adhesive	2.1 – 3.4	[15]
Polyester-based composite	Polyester from the polycondensation of isosorbide, long chain dimer diol and dimer fatty acid	Yes	Yes	Adhesive	0.5 – 10	[16]
Polyester partly derived from plant oils	Polyester from polycondensation of dimer fatty acids in combination with a dimer fatty diol, butane diol or isosorbide; mixed with epoxy plant oil	No	Yes	Adhesive	0.6 – 6.25	[17]
Poly(β-hydroxyorganoate) based composites	Mixture of various random copolymers of poly(β-hydroxyorganoate)s with unsaturated side chains (in 40-70 wt%)	Yes (10-60 wt%)	Yes	Cohesive	0.1 – 13.6	[18]
Resin of poly(3-hydroxyalkanoates) and plant oil	poly(3-hydroxyalkanoates) produced by Gram-negative bacterium using linseed oil fatty acids	Yes	Yes	Cohesive	0.6 – 3.1	[19]
Epoxidized and dihydroxyl soybean oil	Cross-linked mixture of epoxidized and dihydroxyl polymers derived from soybean oil	Yes	Yes	Mostly Adhesive	0.7 – 2.2	[20]
Acrylated methyl oleate polymers	Acrylate polymers based on fatty acids derived from plant oils	No	Yes	Cohesive	1.3 – 2.9	[21]
Copolymers of epoxidized soybean oil and lactic acid oligomers	Polymer network of copolymers of epoxidized soybean oil and lactic acid	Yes	Yes	Mostly Adhesive	0.2 – 5.8	[22]
Poly(alkyl glycidate carbonate)	Glycidyl carboante polymers from the ROCOP of glycidate/glycidyl epoxides with CO_2	No	No	Cohesive	0.1 – 8.0	[23]

*adhesive and cohesive failure indicates PSA cleanly removed and PSA residue remains, respectively

2. References

[1] M. R. Kember, P. D. Knight, P. T. R. Reung, C. K. Williams, *Angew. Chem. Int. Ed.* **2009**, *48*, 931-933.

[2] M. J. Sanford, L. P. Carrodeguas, N. J. Van Zee, A. W. Kleij, G. W. Coates, *Macromolecules* **2016**, *49*, 6394-6400.

[3] A. Spyros, D. S. Argyropoulos, R. H. Marchessault, *Macromolecules* **1997**, *30*, 327-329.

[4] a) P. K. Saini, G. Fiorani, R. T. Mathers, C. K. Williams, *Chem. - Eur. J.* **2017**, *23*, 4260-4265; b) L. P. Carrodeguas, C. Martin, A. W. Kleij, *Macromolecules* **2017**, *50*, 5337-5345.

[5] S. Kobayashi, H. Uyama, T. Takamoto, *Biomacromolecules* **2000**, *1*, 3-5.

[6] a) C. R. Dias, M. F. Portela, M. GalanFereres, M. A. Banares, M. L. Granados, M. A. Pena, J. L. G. Fierro, *Catal. Lett.* **1997**, *43*, 117-121; b) E. Mahmoud, D. A. Watson, R. F. Lobo, *Green Chem.* **2014**, *16*, 167-175; c) S. Giarola, C. Romain, C. K. Williams, J. P. Hallett, N. Shah, *Chem. Eng. Res. Des.* **2016**, *107*, 181-194.

[7] H. Taheri, C. D. Dickinson, P. A. Jacobson, US 2009/0043114A1, **2009**

[8] a) M. Konishi, US20110130600A1, **2011**; b) T. Kato, I. Ichinose, *J. Chem. Soc., Perkin Trans. 1* **1980**, 1051-1056; c) A. Weisz, A. Mandelbaum, *J. Org. Chem.* **1984**, *49*, 2648-2650; d) R. T. Mathers, M. J. Shreve, E. Meyler, K. Damodaran, D. F. Iwig, D. J. Kelley, *Macromol. Rapid. Comm.* **2011**, *32*, 1338-1342.

[9] B. C. Saha, G. J. Kennedy, N. Qureshi, M. J. Bowman, *Biotechnol Progr* **2017**, *33*, 1059-1067.

[10] a) T. A. Nijhuis, M. Makkee, J. A. Moulijn, B. M. Weckhuysen, *Ind. Eng. Chem. Res.* **2006**, *45*, 3447-3459; b) P. S. Kong, M. K. Aroua, W. M. A. W. Daud, *Renewable and Sustainable Energy Reviews* **2016**, *63*, 533-555.

[11] a) X. Ouyang, S. Ian Zones, A. S. Katz, US9687830B2, **2017**; b) M. Winkler, C. Romain, M. A. R. Meier, C. K. Williams, *Green Chem.* **2015**, *17*, 300-306.

[12] a) S. Y. Lee, H. U. Kim, T. U. Chae, J. S. Cho, J. W. Kim, J. H. Shin, D. I. Kim, Y. S. Ko, W. D. Jang, Y. S. Jang, *Nat Catal* **2019**, *2*, 18-33; b) Davis E.M., C. R., in *Topics in Current Chemistry, Vol. 209*, Springer, Berlin, **2000**.

[13] E. P. Chang, *J. Adhesion* **1991**, *34*, 189-200.

[14] G. I. Ozturk, A. J. Pasquale, T. E. Long, *J. Adhesion* **2010**, *86*, 395-408.

[15] Y. SatomiI, T. Hitoshi, WO2009142272A1, **2011**

[16] R. Vendamme, W. Eevers, WO2012045480Al, **2012**

[17] R. Vendamme, W. Eevers, *Macromolecules* **2013**, *46*, 3395-3405.

[18] D. R. Rutherford, W. J. Hammar, G. N. Babu, US5614576A, **1997**

[19] G. A. M. van der Walle, G. J. H. Buisman, R. A. Weusthuis, G. Eggink, *Int. J. Biol. Macromol.* **1999**, *25*, 123-128.

[20] B. K. Ahn, S. Kraft, D. Wang, X. S. Sun, *Biomacromolecules* **2011**, *12*, 1839-1843.

[21] W. Maaßen, S. Oelmann, D. Peter, W. Oswald, N. Willenbacher, M. A. R. Meier, *Macromolecular Chemistry and Physics* **2015**, *216*, 1609-1618.

[22] Y. H. Li, D. H. Wang, X. S. Sun, *RSC Adv.* **2015**, *5*, 27256-27265.

[23] A. Beharaj, I. Ekladious, M. W. Grinstaff, *Angew. Chem. Int. Ed.* **2019**, *58*, 1407-1411.
